# Global patterns of allometric model parameters prediction

**DOI:** 10.1038/s41598-023-28843-2

**Published:** 2023-01-27

**Authors:** Zixuan Wang, Xingzhao Huang, Fangbing Li, Dongsheng Chen, Xiaoniu Xu

**Affiliations:** 1 School of Forestry and Landscape of Architecture, Anhui Agricultural University, Hefei, 230036 China; 2 Key Laboratory of Tree Breeding and Cultivation, State Forestry Administration, Research Institute of Forestry, Chinese Academy of Forestry, Beijing, 100091 China

**Keywords:** Ecology, Forest ecology, Forestry

## Abstract

Variations in biomass-carbon of forest can substantially impact the prediction of global carbon dynamics. The allometric models currently used to estimate forest biomass face limitations, as model parameters can only be used for the specific species of confirmed sites. Here, we collected allometric models LnW = a + b*Ln(D) (n = 817) and LnW = a + b*Ln(D^2^H) (n = 612) worldwide and selected eight variables (e.g., mean annual temperature (MAT), mean annual precipitation (MAP), altitude, aspect, slope, soil organic carbon (SOC), clay, and soil type) to predict parameters *a* and *b* using Random Forest. LnW = a + b*Ln(D), drove mainly by climate factors, showed the parameter *a* range from − 5.16 to − 0.90 [VaR explained (model evaluation index): 66.21%], whereas parameter *b* ranges from 1.84 to 2.68 (VaR explained: 49.96%). Another model LnW = a + b*Ln(D^2^H), drove mainly by terrain factors, showed the parameter *a* range from − 5.45 to − 1.89 (VaR explained: 69.04%) and parameter *b* ranges from 0.43 to 1.93 (VaR explained: 69.53%). Furthermore, we captured actual biomass data of 249 sample trees at six sites for predicted parameters validation, showing the R^2^ (0.87) for LnW = a + b*Ln(D); R^2^ (0.93) for LnW = a + b*Ln(D^2^H), indicating a better result from LnW = a + b*Ln(D^2^H). Consequently, our results present four global maps of allometric model parameters distribution at 0.5° resolution and provides a framework for the assessment of forest biomass by validation.

## Introduction

Carbon assessment, capture and storage are critical components of the global carbon budget that can assist with limiting global temperature increases within 2 °C compared with pre-industrial^1, 2^. Forest play a vital role toward this end as they comprise the dominant terrestrial ecosystem. Specifically, it occupies 30% of the global land area and containing about half the carbon stored in terrestrial ecosystems especially in the form of aboveground biomass (AGB)^3, 4^. Hence, it’s essential for accurate prediction of global forest biomass, which has strong impacts on the global carbon dynamics and its feedback with global warming^5^.

The accurate and timely estimation of forest AGB at various spatial and global scales has been challenging for ecologists for half a century^6^. There are several predictive strategies, including allometric models, widely used with the highest accuracy among all methods. This method is based on forest inventories and allometric relationships between the tree biomass and its trunk diameter, tree height as well as other variables, always built in the form of W = a*D^b^ or W = a*(D^2^H)^b^, where W is the aboveground biomass (kg), D is the tree diameter at breast height (cm), H is the tree height (cm), and *a* and *b* are model parameters^7–9^.

Numerous studies have been conducted on the model in the form of W = a*D^b^ about calculating the value and significance of parameters. The parameter *b* has clear biological characteristics, which is the ratio of specific rates between biomass and diameter^10^. Moreover, previous studies believed that parameter *b* is referred to as the “constant differential growth rate” was equal to 2.67, which has not been definitively verified^11^. In contrast to *b*, the interpretation of parameter *a* of W = a*D^b^ is indistinct. Recently, a study confirmed that tree height also needs to be considered because diameter (D) alone is often insufficient to predict biomass, especially in tropic forests^9^. And the model W = a*(D^2^H)^b^ was proposed as frequently applied to improve the estimation accuracy, which predicts biomass as a function of height and diameter. Currently, these two models are widely used to estimate AGB.

Allometric model is developed for specific species at confirmed locations with unique parameters varying by site and model so that it faces a shortage of localized parameters. In general, parameters are obtained by harvesting and weighing trees to form allometric models via the correlation of tree structure variables such as diameter, height, or other dendrometric variables to the tree biomass using mathematical functions^8, 12^. However, this traditional method is limited by study sites and species which is laborious and time consuming. For example, not all forest regions can be accessed to build allometric models through harvesting and weighing^13^. Besides, there is also a site limitation for model parameters and the estimated accuracy decreases when the parameters are applied to sites outside the geographical locations where the models were originally development^14^. Thus, a systematic analysis of the geographical distribution patterns of parameters under the influence of multiple factors is vital for the application of predicting tree aboveground biomass worldwide.

To address above limitations, we committed to collecting allometric models for various tree species and make predictions by random forest modeling approaches, a stronger predictive model^15^, to answer what is the parameter distribution of terrestrial forests on a global scale? And the main goal in this study was to develop predictive parameter patterns on a large scale. Finally, we would validate the predictive model to demonstrate whether it is available to provide a technical framework for accurately estimating terrestrial forest aboveground biomass storage.

## Materials and methods

### Data collection

Peer-reviewed articles published up to Dec 31, 2021 were searched through the Web of Science (http://webofknowledge.com), Google scholar (http://scholar.google.com), and the China National Knowledge Infrastructure (CNKI, http://www.cnki.net). Here we employed a combination of the following search terms: “(tree biomass OR aboveground biomass OR plant biomass OR plant productivity) and (allometric biomass equation OR allometric model OR productivity model OR biomass equation OR biomass model)”. To avoid potential selection bias and duplicates, we conducted a cross-check between the references of relevant articles, which resulted in the selection of 729 relevant articles from the thousands of the appearing articles initially. Subsequently, eligible articles were selected using the following criteria: (1) Allometric models built for specific species with confirmed locations without disturbances were selected, generalized species, large-scales (e.g., province or nation), as well as recently disturbed tree models were excluded. (2) The method employed to develop the model was destructive harvesting and weighing, with at least twenty sample trees, were selected; articles were excluded that did not include measurements and used less than twenty sample trees. (3) The model forms were W = a*D^b^ and LnW = a + b*Ln(D) or W = a*(D^2^H)^b^ and LnW = a + b*Ln(D^2^H), where W is the aboveground biomass, and D is the diameter at breast height, H is the tree height, were selected. Consequently, we excluded articles with other variables and other forms of models. Finally, 426 articles remained from the original 729 (Supplementary Fig. S1).

We then distilled data from the articles for the following variables: (1) Allometric models, in the form of W = a*D^b^ and LnW = a + b*Ln(D), W = a*(D^2^H)^b^ and LnW = a + b*Ln(D^2^H) including the parameters *a*, *b* in the D range and H range. (2) Tree species corresponding to the models, including families, genera, and species. (3) Location data, including longitude, latitude, and study sites. (4) Climate data, including mean annual temperature (MAT, °C) and mean annual precipitation (MAP, mm) of the tree species location. (5) Terrain data, including slope and aspect. (6) Soil data, including soil organic carbon (SOC), clay, and soil type.

Since not all articles provided the location, climate, soil, and terrain data of the studies, we estimated the missing data as follows, (1) we supplemented the longitude and latitude with the study location using Google Earth. (2) We extracted the missing climate data by using geographic coordinates from WorldClim version 2.0 (http://worldclim.org/current)^16^. (3) We obtained the shuttle radar topographic mission DEM data with 30 m resolution from NASA, and used SAGA-GIS software to derive various terrain data from the DEM such as altitude, slope, and aspect^17, 18^. (4) The missing soil data was derived from the Regridded Harmonized World Soil Database v1.2^19^. In particular, we established the soil type according to Soil Taxonomy to increase the accuracy of the analysis and prediction. Furthermore, if the experiments were performed at multiple sites in one study, they were treated as independent observations. In light of above criteria, 817 allometric models in the form of W = a*D^b^ or LnW = a + b*Ln(D) and 612 allometric models in the form of W = a*(D^2^H)^b^ or LnW = a + b*Ln(D^2^H) were collected from the 426 articles.

### Allometric model

The relationship between the diameter and aboveground biomass was in the form of the power function^20^:

1 $$\begin{array}{c}Wi=a\times D{i}^{b},\end{array}$$

where *Wi* is the dry mass of the ith tree (kg), *Di* is diameter at breast height (cm), and *a* and *b* are the parameters of the model.

2 $$Wi=a\times (D{i}^{2}Hi{)}^{b},$$

where *Wi* is the dry mass of the ith tree (kg), *Di* is diameter at breast height (cm), *Hi* is the tree height (cm), and *a* and *b* are the parameters of the model.

However, a heteroscedasticity exists when directly fitting the tree biomass. The logarithmic transformation of Eq. (1) or Eq. (2), is convenient to facilitate model fitting and deal with heterocedasticity^21^. The logarithmic transformation allometric model:

3 $$\begin{array}{c}Ln\left(Wi\right)=a+b\times Ln\left(Di\right),\end{array}$$

was used in this function, where *a* (Eq. 3) represents *Ln*(*a*) (Eq. 1), and *b* (Eq. 3) is the same as *b* (Eq. 1), respectively.

4 $$\begin{array}{c}Ln\left(Wi\right)=a+b\times Ln\left(D{i}^{2}H\right),\end{array}$$

was used in this function, where *a* (Eq. 4) represents *Ln*(*a*) (Eq. 2), and *b* (Eq. 4) is the same as *b* (Eq. 2), respectively. To unify the models, we transformed the collected Eqs. (1) to (3) and Eqs. (2) to (4).

### Data analysis

To establish the relationship between variables with parameters *a* and *b* for making a parameter prediction on a global scale, Random Forest (RF) (an example of a machine learning model) was employed, which consists of an ensemble of randomized classification and regression trees (CART)^21^. In short, the RF will generate a number of trees and aggregate these to provide a single prediction. In regression problems the prediction is the average of the individual tree outputs, whereas in classification the trees vote by majority on the correct classification^22, 23^. Generated trees called n_tree_ are based on a bootstrapped 2/3 sample of the original data to decrease correlations by choosing different training sets in the RF modeling process^15^. In addition to this normal bagging function, the best split at each node of the tree was searched only among a randomly selected subset (m_try_) of predictors^24^. The tree growing procedure is performed recursively until the size of the node reaches a minimum, k, which is parameterized by the user. For the rest of the original data, RF provides a believable error estimation using the data called Out-Of-Bag (OOB), which is employed to obtain a running unbiased estimate of the classification error as trees are added to the forest^15^.

### Predictive variable selection

The variables included stand factors such as density, family, and diameters, as well as non-stand factors such as MAT, MAP, and SOC. Considering that the prediction was on a global scale, the first step was to exclude the factors that it was not possible to completely extract. Next, we selected variables through the following^22^: (1) the RF classifier was initially applied using all of the predictor variables, and variable importance was used to rank them based on the mean decrease in accuracy. (2) Removing the least important variables by the variable importance ranking, (3) the training data were then partitioned five-fold for cross-validation and the error rates for each of the five cross-validation partitions were aggregated into a mean error rate, and 20 replicates of the five-fold CV were performed^25^.

By means of the above, eleven variables, including family, genus, species, MAT, MAP, altitude, aspect, SOC, slope, clay, and soil type, were remained to predict parameters. Since the combinations of variables were different, five combinations were performed to make predictions from the eleven variables above. Among the five combinations, each were used by RF to predict and select via the model evaluation index VaR explained and the mean of squared residual (Supplementary Table S1).

### Optimization of Random Forest parameters

RF depends primarily on three parameters that are set by users. (1) n_tree_, the number of trees in the forest. (2) nodesize, the minimum number of data points in each terminal node. (3) m_try_, the number of features tried at each node. To obtain the optimization of RF parameters, we set n_tree_ = 1000, 2000, 3000 and the selection criterion was that n_tree_ was small enough to maximize computational efficiency as well as produced stable OOB error^25^. As for nodesize, we used 3, 5, 7, and 5 as the default for regression RF, given that the m_try_ value always is always one third of the number of variables. Here we also set the m_try_ values (ranging from 2 to 4), which were tested, and we accessed the OOB error rates from 50 replicates for each m_try_ value^25^. The primary tuning parameter above were optimized, as well as each combination of the three RF parameters through a grided search, which were used to predict and set RF parameters according to the predictive effect of each combination (Supplementary Table S2).

All above data analysis were conducted in R 4.0.3^26^. And the output is the spatial pattern of allometric model parameters at 0.5° resolution.

### Predicted parameter validation

Further to assess the accuracy of the predicted parameters, we applied them to estimate the AGB at six sites. And the actual AGB of the sites had been obtained via destructive sampling from 209 plots, which were located in Hubei, Liaoning, Gansu, Hebei and Heilongjiang provinces, and Inner Mongolia autonomous region from 2009 to 2013^27^ (Table 1). First, we selected the sample trees according the dominant, average and inferior tree outside the plot. Then the sample trees were felled as carefully as possible and tree height (H), tree diameter in the breast (DBH) and live crown length were recorded. To divide trees into several sub-samples, including branches, leaves, stem wood and stem bark, all of the branches were removed and leaves were picked. Besides, stem was divided into 1 m sections and bark of the stem was removed. Finally, all sub-samples of aboveground part of trees were oven-dried at 80 °C until a constant weight was reached and the sum of all the sun-samples weight was the actual AGB. Through the above process, 249 actual AGB data were obtained. Meanwhile, the predicted parameters of the models together with the DBH and H estimated the predicted AGB. The actual AGB data of 249 sample trees were compared with the predicted AGB by making fitting curves between them in R to show the availability of predicted parameters according root mean square error (RMSE) and R^2^.

**Table 1:** The basic features of the sampling sites.

site	Site	Province	Location	Biomass (kg)	DBH (cm)	H (m)
S1	Changlinggang Farm	Hubei province	30.48° N 110.02° E	4.68–236.96	5.0–27.0	5.0–24.0
S2	Tianshui city	Gansu province	34.09° N 105.52° E	1.90–260.66	3.0–28.0	4.1–22.4
S3	Weichang county	Hebei province	41.43° N 118.70° E	3.66–237.66	4.2–22.8	4.5–17.6
S4	Dagujia Farm	Liaoning province	42.21° N 124.52° E	4.49–374.89	5.7–28.2	7.1–25.8
S5	Mengjiagang Farm	Heilongjiang province	46.32° N 129.10° E	2.89–193.95	3.4–23.1	3.7–20.8
S6	Wuerqihan Forestry bureau	Inner Mongolia province	49.34° N 121.25° E	1.63–286.19	3.1–26.0	3.6–21.1

The experimental research and field studies on plants in this study, including the collection of plant material, complied with the relevant institutional, national, and international guidelines and legislation. And we ensured that we have permission for the plant sampling, all of the steps were allowed in our study for the plant research. In addition, plant identification in this study was conducted by X.Z according to World Plants (https://www.worldplants.de) in the herbarium of School of Forestry & Landscape of Architecture, Anhui Agricultural University, and the voucher specimen of all plant material has been deposited in a publicly available herbarium.

## Results

### Global occurrence of allometric models

**Figure 1:**
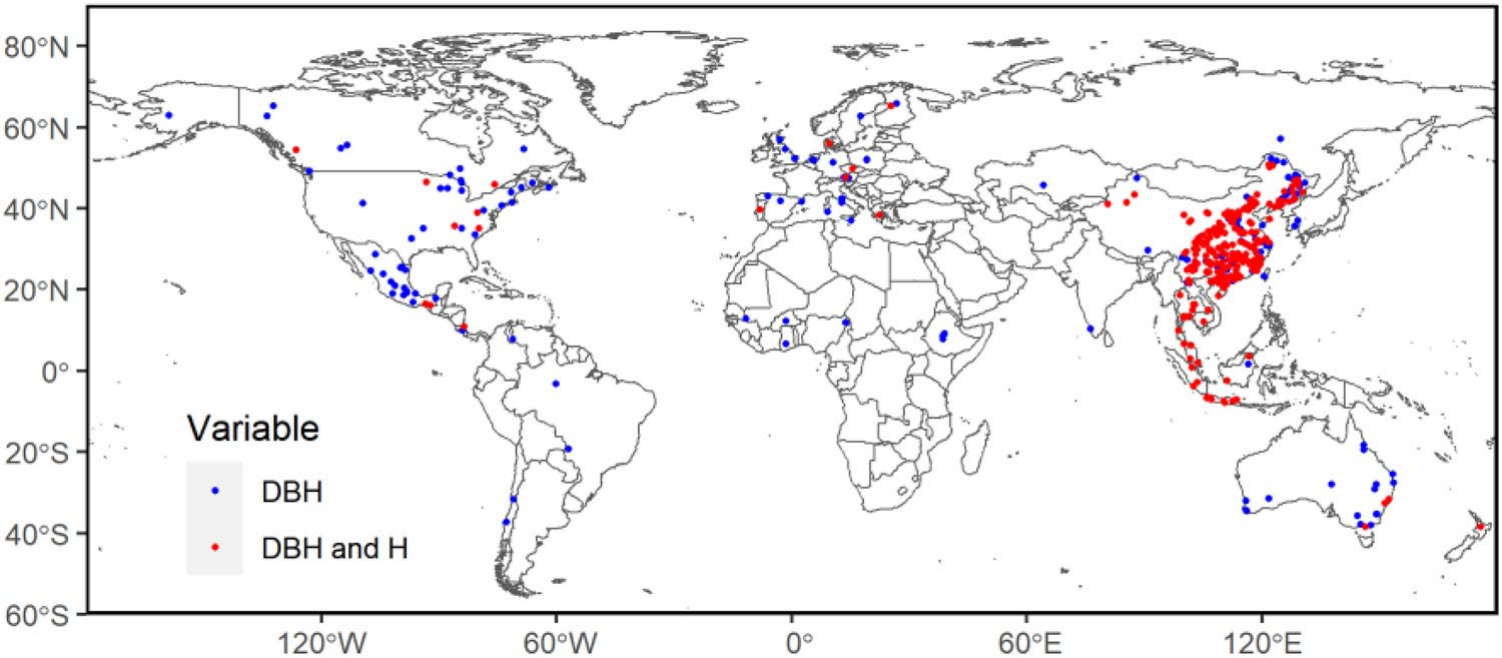
Geographical distribution of the collected allometric models. Blue circles represent the distribution of sites for the DBH (tree diameter at the breast height) alone model (LnW = a + b*Ln(D)) parameters; Red circles represent the distribution of sits for the DBH and H (tree height) model (LnW = a + b*Ln(D^2^H)) parameters. The map is created in R 4.0.3 (URL https://www.R-project.org/.).

The dataset of allometric models was with an extensive distribution range (Fig. 1). In terms of latitude, the parameters focus primarily on 70° N to 40° S, whereas for longitude, almost all parts of the land had distributed parameters. From another perspective, parameters were scattered across all of the continents. The parameters concentrated on the East and West Coasts of the Americas. In Europe, Northern and Central Europe were the main distribution sites, and only the Western and Southern Australia had parameter distribution, including some islands. The study sites in Asia were mainly distributed in the Southeast, especially the DBH and H models. And the parameters were on both sides of the equator in Africa. Overall, the model parameters we selected included sites with MAT of – 10–30 °C, MAP of 100–3600 mm, and altitude of 0–2500 m. Consequently, the allometric model parameters were distributed across a range of geographical, climatic, and forest areas.

### The critical variables of predicted allometric model parameters

**Figure 2:**
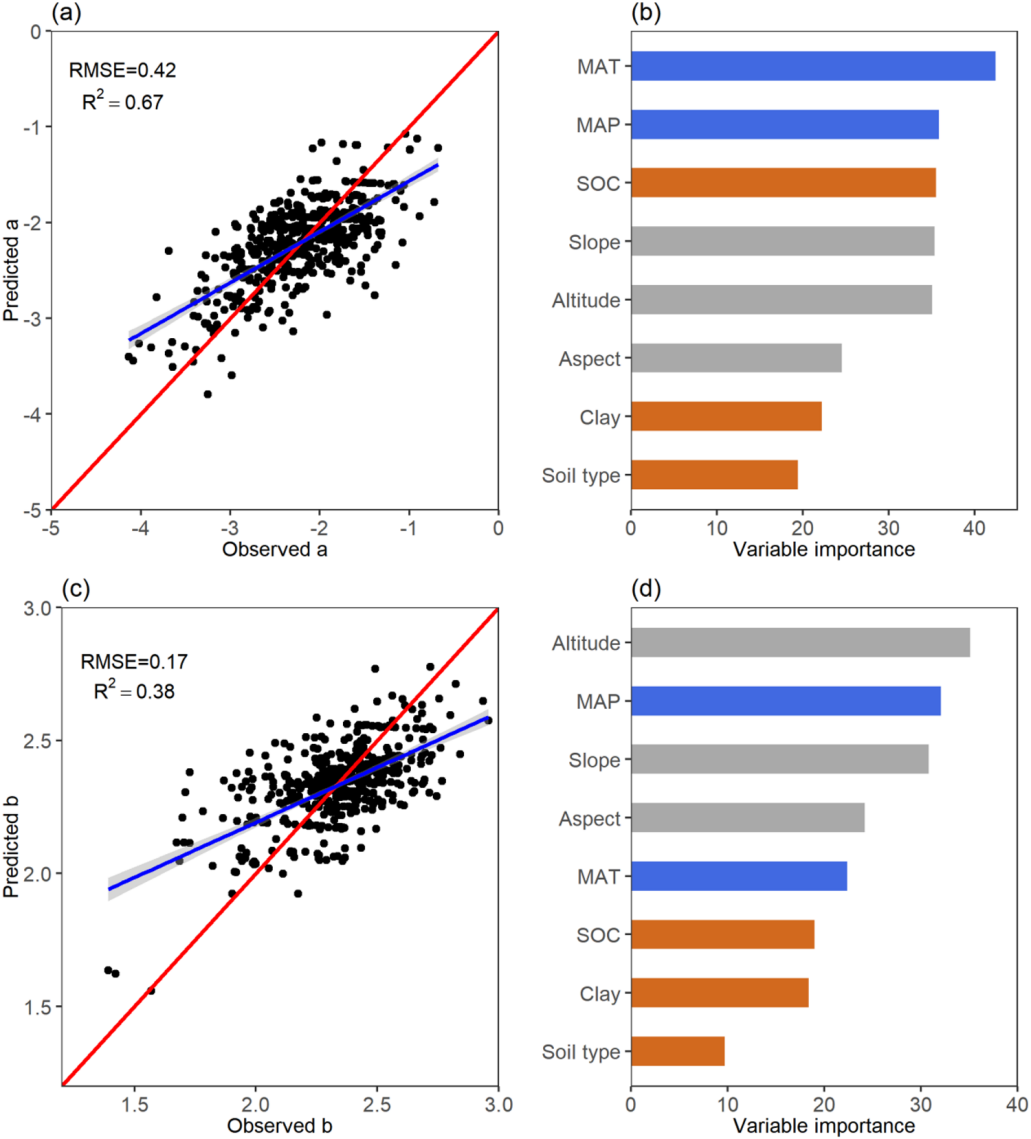
Model performance and variable importance of allometric model (LnW = a + b*Ln(D)) parameters predictions. (**a**) Model performance for parameter *a*; (**c**) Model performance for parameter *b*. RMSE indicates root mean square error; R^2^ indicates R squared. (**b**) Variable importance in predicting parameter *a*; (**d**) Variable importance in predicting parameter *b*. Blue bars represent climatic factors; gray bars represent terrain factors; brown bars represent edaphic factors.

**Figure 3:**
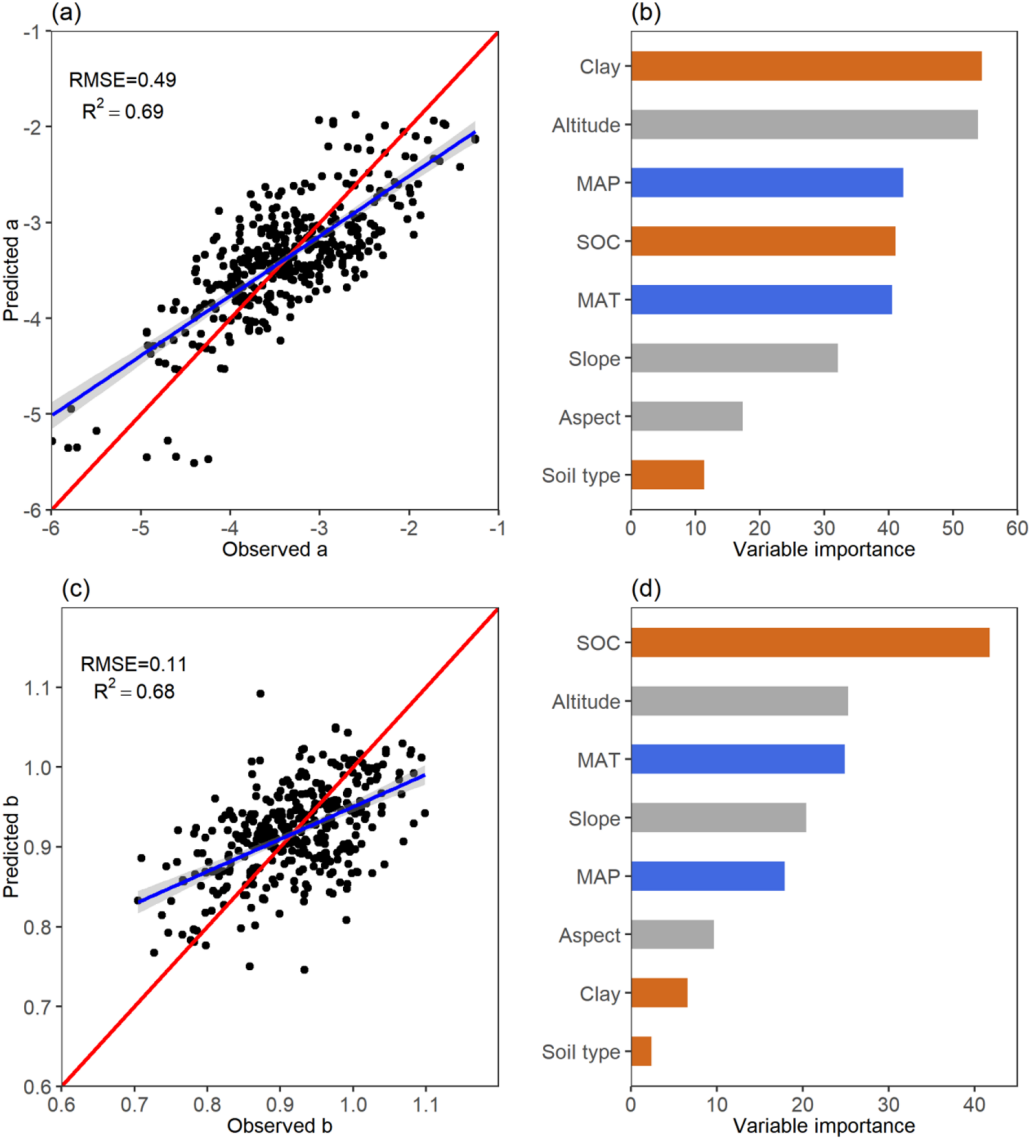
Model performance and variable importance of allometric model (LnW = a + b*Ln(D^2^H)) parameters predictions. (**a**) Model performance for parameter *a*; (**c**) Model performance for parameter *b*. RMSE indicates root mean square error; R^2^ indicates R squared. (**b**) Variable importance in predicting parameter *a*; (**d**) Variable importance in predicting parameter *b*. Blue bars represent climatic factors; gray bars represent terrain factors; brown bars represent edaphic factors.

Five combinations of variables were employed to predict parameters in RF. The best predictive effect combination including all eleven variables. However, it was difficult to obtain current global dataset of tree species so that we had to select MAT, MAP, altitude, aspect, slope, SOC, clay, and soil type as a group to predict the parameters (Supplementary Table S1). As for LnW = a + b*Ln(D), the model VaR explained is 66.21% for parameter *a* and 49.96% for parameter *b* by setting n_tree_ = 3000, m_try_ = 3 and nodesize = 3, which performed well in explaining variability and with reasonable uncertainty in both parameter *a* (R^2^ = 0.67, RMSE = 0.42) as well as parameter *b* (R^2^ = 0.38, RMSE = 0.17) (Fig. 2a,c; Supplementary Table S2). Similarly, LnW = a + b*Ln(D^2^H) had a strong model with 69.04% and 69.53% VaR in parameter *a* and parameter *b*, respectively. It also performed well in explaining variability and with reasonable uncertainty in both parameter *a* (R^2^ = 0.69, RMSE = 0.49) and parameter *b* (R^2^ = 0.68, RMSE = 0.11) (Fig. 3a,c).

The results showed that parameters of the LnW = a + b*Ln(D) were more strongly affected by the climatic factors, but the LnW = a + b*Ln(D^2^H) were mainly drove by terrain factors. For LnW = a + b*Ln(D), the climatic, terrain, and edaphic variables played an important role in the prediction of parameters *a* and *b*. Among these variables, MAT, MAP, and SOC primarily drove the variations of parameter *a*, whereas altitude, MAP, and slope mainly drove the changes of parameter *b* (Fig. 2b,d). The MAT had a positive effect on parameter *a*. For LnW = a + b*Ln(D^2^H), clay and altitude are the main factors for parameter *a* prediction, followed by MAP, SOC, slope and MAT. And SOC played a vital role in predicting parameter *b* (Fig. 3b,d)*.*

### Global pattern of allometric model parameters for terrestrial forest

**Figure 4:**
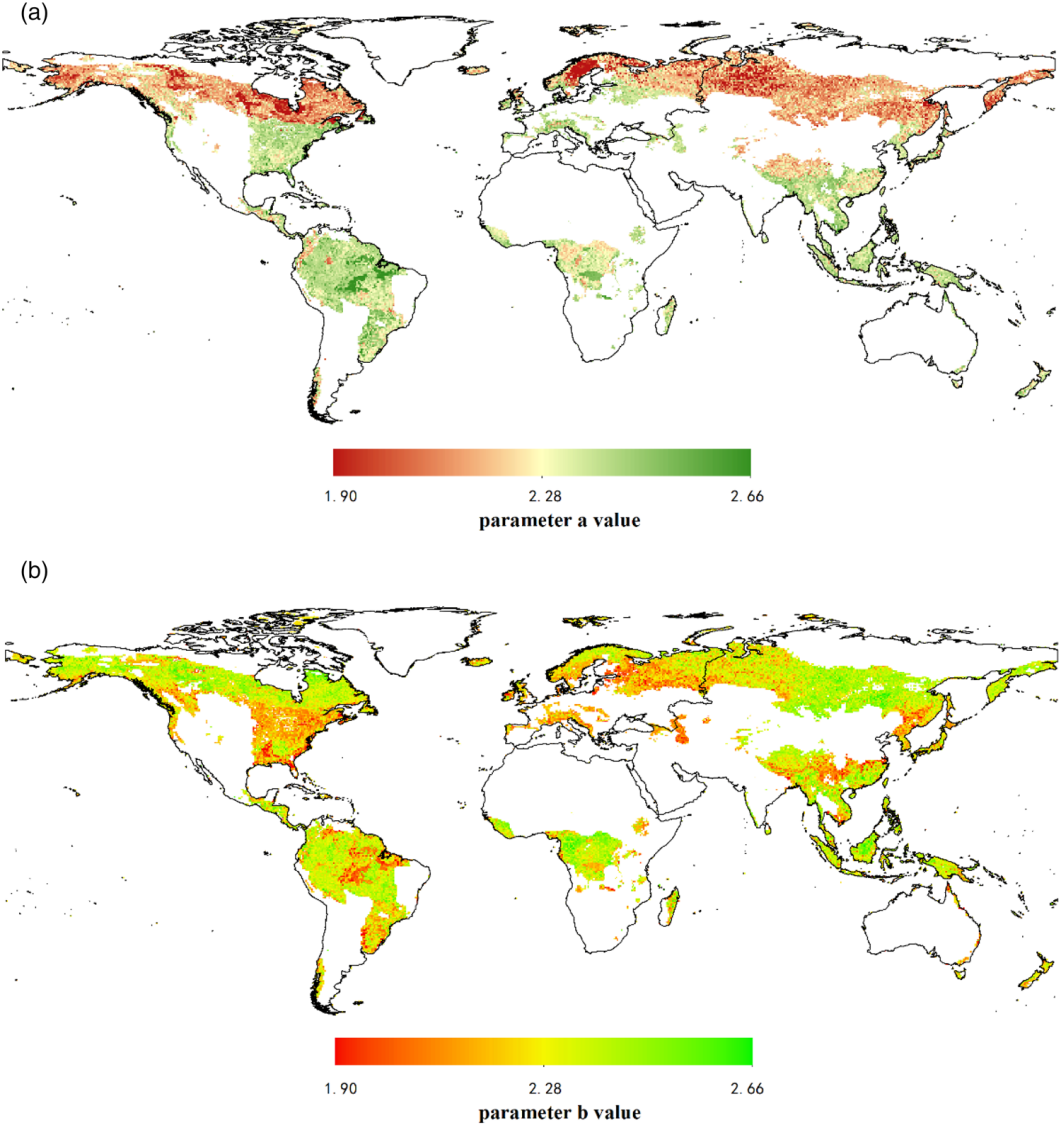
Global pattern of allometric model (LnW = a + b*Ln(D)) parameters prediction map. (**a**) Parameter *a* value. (**b**) Parameter *b* value. The maps are created in R 4.0.3 (URL https://www.R-project.org/) and QGIS 3.16.0 (URL https://qgis.org).

The allometric model parameters had obvious spatial differences in forest ecosystems, especially parameter *a*. The value of parameter *a* ranged from − 5.16 to − 0.90 with obvious latitude patterns in LnW = a + b*Ln(D) (Fig. 4a). Specially, parameter *a* had a lower value in cold temperate zones, as well as cold zones, contrary to higher values in the subtropics and tropics. Particularly in South America, parameter *a* had the highest value due to lying in large regions of tropical rainforest. In contrast, the parameter *b* was not regular in latitude with the value of 1.84 ~ 2.68 (Fig. 4b).

**Figure 5:**
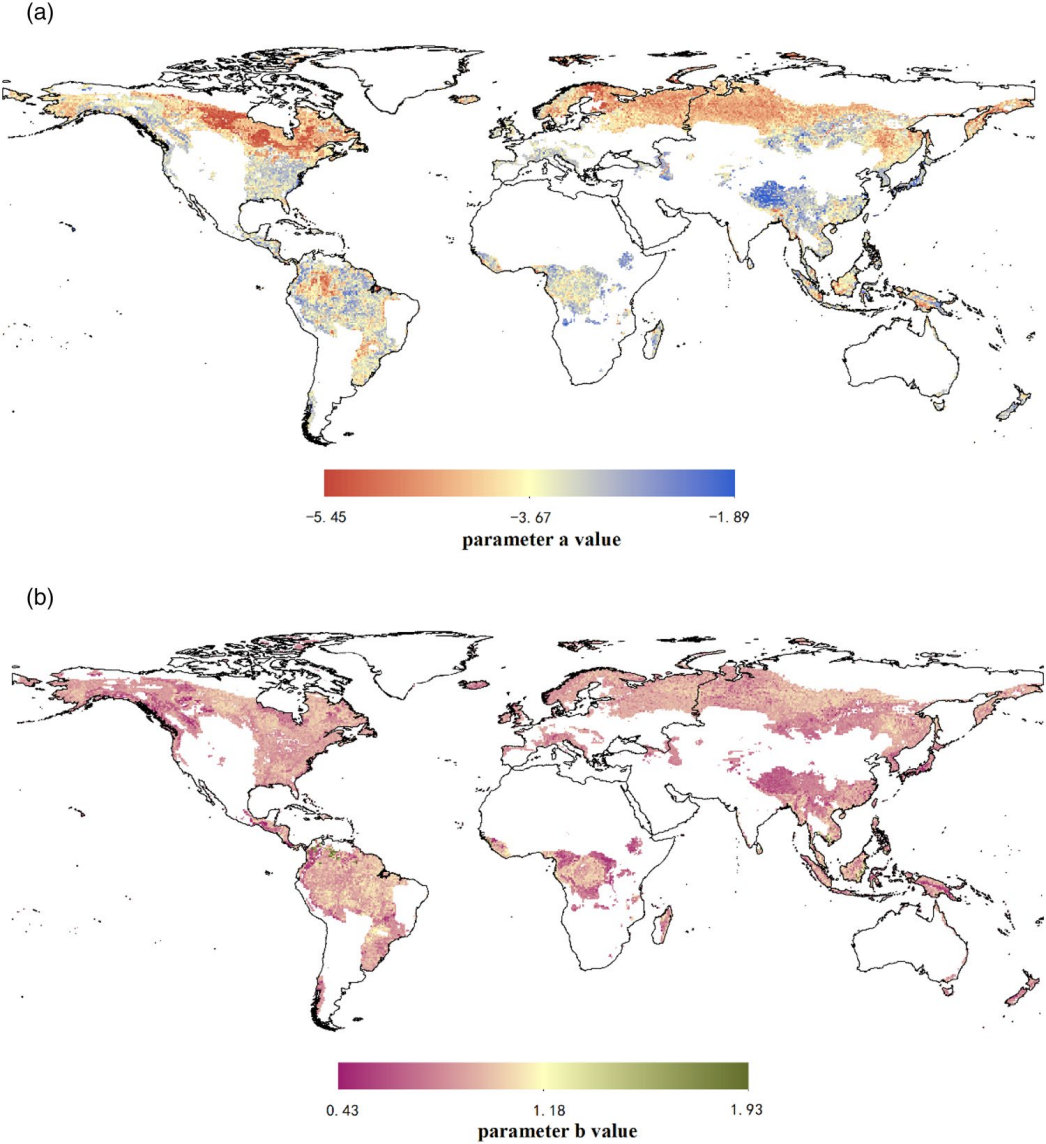
Global pattern of allometric model (LnW = a + b*Ln(D^2^H)) parameters prediction map. (**a**) Parameter *a* value. (**b**) Parameter *b* value. The maps are created in R 4.0.3 (URL https://www.R-project.org/) and QGIS 3.16.0 (URL https://qgis.org).

For LnW = a + b*Ln(D^2^H), the value of parameter *a* ranged from − 5.45 to − 1.89 (Fig. 5a). The low value of parameter *a* mainly focused on high latitude and the subtropics and tropics were distributed with high parameter *a* value. The value of parameter *b* was 0.43–1.93, with a more uniform distributed globally (Fig. 5b).

### Validation effects

**Figure 6:**
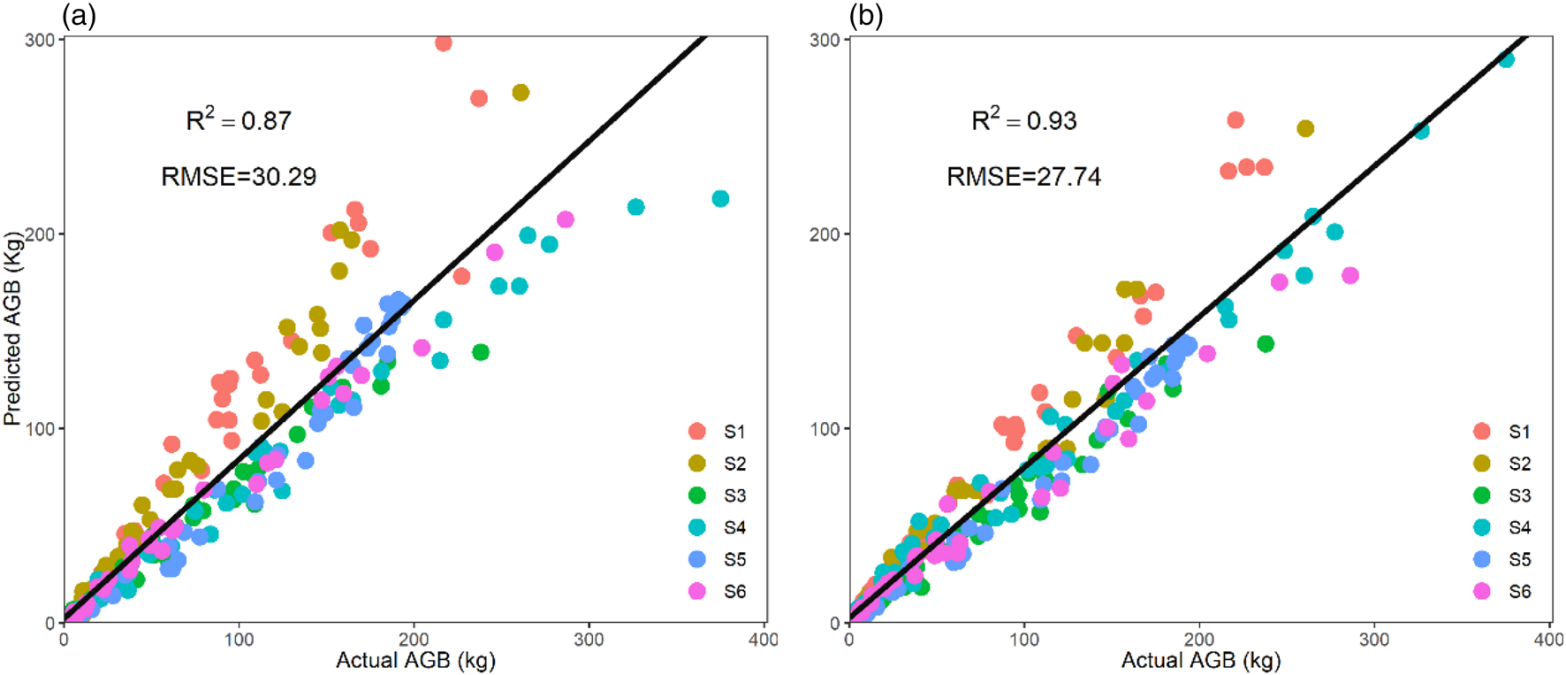
The validation effect of the actual aboveground biomass and the predicted aboveground biomass. (**a**) The predicted biomass estimated by model LnW = a + b*Ln(D). (**b**) The predicted biomass estimated by model LnW = a + b*Ln(D^2^H). S1–S6 represented the six sampling sites for 249 actual biomass data. RMSE indicates root mean square error; R^2^ indicates R squared.

The predicted parameters in the two models can be applied into the biomass estimation well, especially the LnW = a + b*Ln(D^2^H) (Fig. 6). By fitting the actual AGB and predicted AGB at six sampling sites, the first model LnW = a + b*Ln(D) had a good simulation effect (R^2^ = 0.87, *p* < 0.001). And the model LnW = a + b*Ln(D^2^H) is better than the first (R^2^ = 0.93, *p* < 0.001). The results indicated that the predicted parameters in our study can be applied to the actual biomass estimation.

## Discussion

Our analysis represented the global patterns of two allometric models parameters distribution predicted by various environmental factors. And we also applied the predicted parameters of the models to estimate biomass at six sampling sites to ensure model availability by validating with 249 actual biomass data. The results overcame the limitation that parameters can only be used for confirmed sites, which provides reference for estimating forest biomass on a global scale.

For the first model LnW = a + b*Ln(D), the predicted value of parameter *a* ranged from − 5.16 to − 0.90, and regularly decreased with increasing latitude. Parameter *a* had a lower value ranged from − 5.16 to − 3.03 at high latitudes of cold and cold temperate zones, but a higher value ranged from − 2.30 to − 0.90 in subtropical and tropical regions. This pattern was consistent with the results of correlation analysis, which indicated that MAT had a positive effect on parameter *a*. In other words, the positive role caused by MAT to parameter *a* matched with the negative role caused by latitude (Supplementary Fig. S2). The predicted value of parameter *b* varied significantly globally and ranged from 1.84 to 2.68. Researches previously believed that the parameter *b* value of the allometric relationship LnW = a + b*Ln(D) was typically not invariant and predicted *b* = 8/3, which was tested to probe the fractal theory of previous work to use in upcoming non-destructive allometric estimations^11^. However, our study revealed that *b* was not stable as before. Compared with Zianis et al.^28^ that the average *b* value was 2.37 [confidence interval (CI) 2.34, 2.40] for global forests from 279 models, our results showed the predicted value of parameter *b* was 2.39 (CI 2.27, 2.41) for terrestrial forests. Furthermore, we found the predicted value of parameter *b* [2.38 (CI 2.25, 2.41)] was similar to Návar^29^ [2.38 (CI 2.28, 2.48)] in America based on 78 models (Supplementary Table S3). Therefore, parameter *b* should not be regarded as a fixed value, otherwise the biomass would be overestimated.

For the second model LnW = a + b*Ln(D^2^H), the predicted value of parameter *a* was − 5.45 to − 1.89, and also regularly decreased with latitude (Supplementary Fig. S3). The relative high value of parameter *a* was located in subtropics and tropics because of the MAT. But parameter *b* distributed evenly on a global scale. The global distributions of parameters are with spatial difference due to the environment factors; however, it seems that parameter *a* is more sensitive to the environment. One reason is that parameter *b* varies less according to the intrinsic of allometric model compared with parameter *a*^30^. Moreover, the diameter range was concentrated between 5 and 50 cm in our dataset, which reduced the variations of parameter *b* during the fitting of the allometric model. In contrast, parameter *a* means that the biomass that is measured by the harvesting method when the stand canopy is closed, was rarely affected by diameter^31^. It also might be attributed to the effect of non-environmental factors on parameter *b*, such as stand characteristics (e.g., species composition, stand density, growth strategy), management practices, management objectives, that were not selected in this study^8, 32^.

Both of the models (LnW = a + b*Ln(D), LnW = a + b*Ln(D^2^H)) played well because of the high VaR explained and the models show splendid explanations for variability and with reasonable uncertainty in parameter *a* as well as parameter *b*. As for the drivers of the parameters in the two models, the parameters of the LnW = a + b*Ln(D) is mainly drove by climatic factors. Whereas another model parameters drove by terrain factors, which suggested soil properties seem to be significant factor for tree height growth. And no matter what the models, parameter *a* showed the trend with latitude due to the environment and parameter *b* with a more evenly distributed globally. Recent studies emphasized that the second model, including D and H, should be frequently applied, particularly in tropics^9^. And our results revealed LnW = a + b*Ln(D^2^H) was likely to make better predictions worldwide as the validation effect has proved the model had a higher degree accuracy than LnW = a + b*Ln(D).

The variables in allometric models had many arguments. Some studies believed that DBH has been found to be the best predictor of AGB without much improvement from height as an additional parameter^33^. But Chave et al.^34^ tested that if total tree height is available, allometric models usually yield less biased estimates. In general, tree height has often been ignored because measuring tree height accurately is difficult in closed-canopy forests^35, 36^. From our results, we may take both DBH and H into allometric model parameters with the development of forest management and measuring technique in the future.

The allometric model parameters were obtained by destructively harvesting and measuring which is laborious and time consuming, and can only be used in small samples, challenging to implement at a national level^7^. For this study, we cost-effectively integrated publicly available data into a global allometric model parameter framework that estimated forest biomass over large spatial scales. Our analysis demonstrated that the global patterns of two allometric models parameters distribution; then we verified their effectiveness in estimating forest biomass by applied predicted parameters of two models in six sampling sites. The validation effect demonstrated both of the two models can be used to estimate biomass with a high accuracy but better from LnW = a + b*Ln(D^2^H) so that it will make contribution to direct production and management as well as policy formulation.

The prediction of parameters in this study can provide some applications. Firstly, further forest biomass estimation must combine forest resource investigation data (particularly DBH and H) with parameters to obtain accurate forest biomass values. Secondly, remote sensing and LiDAR are commonly used to estimate forest biomass for large regions. The results of this study may be employed as a correction of estimation results to improve the accuracy of global forest biomass calculations. Finally, as forest ecosystems function as critical carbon sinks, tree biomass is paramount for controlling carbon emissions and carbon neutrality. This allometric model application will accurately and timely estimate forest biomass and the substantial effects of this pattern will provide guidance for forest-based carbon management strategies^37, 38^.

In addition to above applications, the potential uncertainties remain in estimating parameters. First, the model predictions are more applicable for small or medium diameter trees to estimate forest biomass not for large diameter trees, especially for the diameter range and height range from the collected data which is limited by the references selected. Because the traditional method always chooses medium or small diameter trees to harvest and weigh to build allometric models^33^. Then, we only pay attention to the harvest method to build models and excluded other methods such as “crown mapping” and terrestrial laser scanning, causing a limited coverage^33, 39^, and some input variables that we failed to obtain from articles were estimated which would make difference in prediction globally. Finally, when compared with other studies involving in forest and plants phenomena predictions by RF^15^, this study achieved a qualified accuracy but not so good as previous. It can be explained by the optimized hyperparameters of the RF using crossing validation and OOB error or the various model evaluation index, which should be explored in the future studies to improve machine learning accuracy.

We used Random Forest to predict two allometric models (LnW = a + b*Ln(D) and LnW = a + b*Ln(D^2^H)) parameters distribution globally. The main results were the global pattern of parameters distribution for two models that parameter *a* was regularly decreased with increasing latitude, but parameter *b* distributed evenly on a global scale both the models. Moreover, by validation of actual biomass, we found that both models had the high accuracy and availability of predicted parameters in estimating biomass but better in LnW = a + b*Ln(D^2^H)). Consequently, both DBH and H should be taken into consideration to estimate biomass by allometric model in the future. Overall, we put forward to a new perspective, Non-Destructive Sampling, in allometric model parameters as well as an available method for estimating forest biomass on a global scale.

## Supplementary Information

Supplementary Information.

## Data Availability

Data used in this study can be found on line in the Supporting Information section at the end of the article.
