# Supplementary material for: Global patterns of allometric model parameters prediction
Source: Sci Rep. 2023 Jan 27;13:1550. doi: 10.1038/s41598-023-28843-2 (PMC9883259; doi:10.1038/s41598-023-28843-2)
Supplement: Supplementary file 1 — Supplementary Information. [file 41598_2023_28843_MOESM1_ESM.zip › Supplement materials/Dataset references.docx]

**Global patterns of allometric model parameters prediction**

**Text 1.** Lists of references used to build the global database of allometric model (Variable: DBH) parameters.

**Previous published syntheses referred to build the global database**

1 Ai, X.R., and Shen, Z.K. Growth and biomass of Larix kaempferi plantation, Journal of Hubei Institute for Nationalities (Natural Sciences), 19(2), 20-22, 2001 (in Chinese).

2 Ai, X.R., and Zhou, G.L. The biomass of Chinese fir plantation in the north boundary of middle subtropical zone, Hubei Forestry Science and Technology, (2), 17-20, 1996 (in Chinese).

3 Ai, X.R., Shen, Z.K., and Yi, Y.M. Effect of stand density on the biomass of Pinus massoniana plantation, Hubei Forestry Science and Technology, (3), 16-18, 1998 (in Chinese).

4 Ai, X.R., Yao, L., Yi, Y.M., and Shen, Z.K. Carbon storage of Cryptomeria fortunei plantation in Enshi Autonomous Prefecture, Journal of Hubei Institute for Nationalities (Natural Sciences), 19(2), 20-22, 2011 (in Chinese).

5 Bai, Y.Q., and Zhan, H.Z. The biomass of Larix gmelinii plantation, Forest Investigation Design, (1), 21-25, 1980 (in Chinese).

6 Bao, X.C., Chen, L.Z., Chen, Q.L., Ren, J.K., Hu, Y.H., and Li, Y. The biomass of planted oriental oak (Quercus variabilis) forest, Acta Phytoecologica et Geobotanica Sinica, 8(4), 313-320, 1984 (in Chinese).

7 Cao, J.X., Wang, X.P., Tian, Y., Wen, Z.Y., and Zha, T.S. Pattern of carbon allocation across three different stages of stand development of a Chinese pine (Pinus tabuliformis) forest, Ecological Research, 27, 883-892, 2012.

8 Chai, B.F., Zhang, J.T., Qiu, Y., and Zheng, F.Y. Aboveground biomass and productivity of Larix principis-rupprechtii artificial forest in the west of Shanxi Province, Henan Science, 17(S), 68-71, 1999 (in Chinese).

9 Chen, C.G. Biomass equations of Korean pine plantation, Forest Investigation Design, (2), 19-23, 1981 (in Chinese).

10 Chen, C.G. Biomass and productivity of tree layers in Pinus armandii forests, Qinling Mountains, Journal of Northwestern College of Forestry, (1), 1-18, 1984 (in Chinese).

11 Chen, Q.C. Study on the Primary Productivity of an Evergreen Broadleaved Forest Ecosystem, Hangzhou University Press, Hangzhou, China, 1993 (in Chinese).

12 Chen, W.R. Study on the dynamics of aboveground net productivity of Alniphyllum fortunei plantation, Journal of Fujian Forestry Science and Technology, 27(3), 31-34, 74, 2000 (in Chinese).

13 Chen, X.G. The biomass and allometric equation of a 20-years-old Cunninghamia lanceolata plantation, Protection Forest Science and Technology, (4), 28-29, 40, 2007 (in Chinese).

14 Chen, C.G., and Guo, X.F. The biomass of broadleaved Korean pine forest, Forest Investigation Design, (2), 10-19, 6, 1984 (in Chinese).

15 Chen, C.G., and Peng, H. Standing crops and productivity of the major forest types at Huoditang Forest Region of Qinling Mountains, Journal of Northwest Forestry College, 11(S), 92-102, 1996 (in Chinese).

16 Chen, C.G., and Zhu, J.F. Manual on Biomass Equations of Major Tree Species in Northeast China, China Forestry Publishing House, Beijing, China, 1989 (in Chinese).

17 Chen, C.G., Gong, L.Q., Peng, H., and Liu, X.Z. Biomass and productivity of Quercus aliena var. acuteserrata forests in Qinling Mountains, Journal of Northwest Forestry College, 11(S), 103-114, 1996 (in Chinese).

18 Chen, L.Z., Ren, J.K., Bao, X.C., Chen, Q.L., Hu, Y.H., Miao, Y.G., and Li, Y. Community characteristics and biomass of Chinese pine plantation in Xishan Region, Beijing, Acta Phytoecologica et Geobotanica Sinica, 8(3), 173-181, 1984b (in Chinese).

19 Chen, L.Z., Chen, Q.L., Bao, X.C., Ren, J.K., Miao, Y.G., and Hu, Y.H. Study on Chinese arborvitae (Platycladus orientalis) forest and its biomass in Beijing, Acta Phytoecologica et Geobotanica Sinica, 10(1), 17-25, 1986a (in Chinese).

20 Chen, X.Y., Peng, Y.Y., and Kang, W.X. Study on biomass and productivity of a subtropical evergreen broadleaved forest dominated by Engelhardtia roxburghiana and Schima superba, in: Long-term Located Research on Forest Ecosystem, Liu, X.Z., Kang, W.X., Chen, X.Y., Wen, S.Z., eds., China Forestry Publishing House, Beijing, China, 68-72, 1993a (in Chinese).

21 Chen, Z.H., Zhang, H.D., Wang, B.S., and Zhang, Z.Q. The biomass and its allocation of an evergreen broadleaved forest in Heishiding, Guangdong, Acta Phytoecologica et Geobotanica Sinica, 17(4), 289-298, 1993b (in Chinese).

22 Cheng, T.R., Ma, Q.Y., Feng, Z.K., and Luo, X. Study on forest biomass in Xiaolong Mountains, Gansu Province, Journal of Beijing Forestry University, 29(1), 31-36, 2007 (in Chinese).

23 Cheng, Y., Hong, W., Wu, C.Z., and Qi, X.H. Aboveground biomass and its productivity of Schima superba population, Chinese Journal of Applied and Environmental Biology, 15(3), 318-322, 2009 (in Chinese).

24 Cui, W., Mu, C.C., Lu, H.C., Bao, X., and Wang, B. Effects of draining for forestation on carbon storage of wetland ecosystem in Daxing’an Mountains of northeastern China, Journal of Beijing Forestry University, 35(5), 28-36, 2013 (in Chinese).

25 Dai, H.J., He, H.J., Zhao, X.H., Zhang, C.Y., Wang, J.S., and Yang, S. Biomass allocation patterns and allometric models of two dominant tree species in broadleaved and Korean pine mixed forest, Chinese Journal of Applied and Environmental Biology, 19(4), 718-722, 2013 (in Chinese).

26 Dang, C.L., and Wu, Z.L. Study on the biomass of Pinus yunnanensis forest. Acta Botanica Yunnanica, 13(1), 59-64, 1991 (in Chinese).

27 Dang, C.L., and Wu, Z.L. Study on the biomass of a monsoon evergreen broadleaved forest dominated by Castanopsis echidnocarpa, Journal of Yunnan University (Natural Sciences), 14(2), 95-107, 1992 (in Chinese).

28 Dang, C.L., and Wu, Z.L. Study on the biomass of Castanopsis orthacantha community, Journal of Yunnan University (Natural Sciences), 16(3), 195-199, 1994 (in Chinese).

29 Dang, C.L., Yu, H., and Li, Y. Study on the relationship between ecological factors and overstory biomass in Pinus yunnanensis forest, Journal of Yunnan University (Natural Sciences), 14(2), 146-151, 1992 (in Chinese).

30 Dang, C.L., Wu, Z.L., and Zhang, Q. Study on the biomass of the ravine tropical rain forest in Xishuangbanna, Acta Botanica Yunnanica, (S8), 123-128, 1997 (in Chinese).

31 Ding, Z.F., Jiang, C.W., Wang, X.J., and Wu, Z.M. Forest biomass and productivity of main evergreen communities in Xiaokeng Watershed, Anhui Province, Journal of Nanjing Forestry University (Natural Science Edition), 33(2), 129-133, 2009 (in Chinese).

32 Ding, B.Y., and Sun, J.H. Study on biological productivity and nutrient cycling of artificial Korean pine forest ecosystem, Journal of Northeast Forestry University, 17(S), 1-98, 1989 (in Chinese).

33 Dong, L.H., Li, F.R., and Jia, W.W. Development of tree biomass model for Pinus koraiensis plantation, Journal of Beijing Forestry University, 34(6), 16-22, 2012 (in Chinese).

34 Dong, L.H., Li, F.R., and Jia, W.W. Effects of tree competition on the biomass and biomass models of Pinus koraiensis plantation, Journal of Beijing Forestry University, 35(6), 15-22, 2013 (in Chinese).

35 Du, H., Song, T.Q., Zeng, F.P., Wen, Y.G., and Peng, W.X. Biomass and its allocation in Pinus massoniana plantation at different stand ages in eastern Guangxi Province, Acta Botanica Boreali-Occidentalia Sinica, 33(2), 394-400, 2013 (in Chinese).

36 Fang, W., and Wang, G.Q. Biomass and productivity of farmland shelterbelt network, Forest Science and Technology, (6), 11-13, 1989.

37 Fan, S.H., Liu, G.L., Zhang, Q., Feng, H.X., Zong, Y.C., and Ren, H.Q. Biomass and productivity of Populus xiaohei plantation on sandy land in north China, Forest Research, 23(1), 71-76, 2010 (in Chinese).

38 Gao, C.J., Tang, G.Y., Sun, Y.Y., Zhang, C.H., Xie, Q.H., and Li, K. Biomass and allocation of young Azadirachta indica and Acacia auriculiformis for different restoration patterns in dry-hot valley, Journal of Zhejiang A&F University, 29(4), 482-490, 2012 (in Chinese).

39 Gao, H.Y., Zhou, G.Y., Zhou, Z.P., Zhao, H.B., and Qiu, Z.J. Aboveground biomass of 27-year-old Cunninghamia lanceolata plantation in Tianjingshan Forest Farm, Guangdong Province, Guangdong Forestry Science and Technology, 29(4), 1-6, 2013 (in Chinese).

40 Gao, H.Z., You, L.Q., and Wang, C. Individual biomass and productivity of Pinus tabuliformis plantation in Yanshan Mountain, Journal of Hebei Forestry Science and Technology, (4), 7-9, 2009 (in Chinese).

41 Guan, H.S., and Liu, Y.L. Study on the biomass of poplar plantations in the middle region of “One River and Two Streams” Watershed, Tibet, Forest Science and Technology, (9), 20-22, 32, 1993 (in Chinese).

42 Guo, L.Q., and Xiao, Y. The biomass table of Larix principis-rupprechtii natural forest, Forest Resources Management, (5), 36-39, 1989 (in Chinese).

43 Guo, X.Y., Cai, T., Duan, X.W., Han, Y.J., Huang, D., and Da, L.J. Carbon storage and distribution pattern in main economic fruit forest ecosystems in Shanghai, East China, Chinese Journal of Ecology, 32(11), 2881-2885, 2013 (in Chinese).

44 He, F., Wang, Y.Q., Tan, X.F., and Wang, C.N. The biomass and nutrient cycle in Vernicia fordii plantations, Non-wood Forest Research, 8(2), 6-20, 1990 (in Chinese).

45 Hong, Y.C., Xu, W.Q., Ye, G.F., and Zhang, L.H. Model for estimating biomass of Casuarina equisetifolia planation in coastal region of the southeastern China, Journal of Zhejiang Forestry Science and Technology, 30(4), 66-69, 2010 (in Chinese).

46 Hu, J.R., Huang, R.K., Zeng, H.D., Li, P.Y., and Wang, Z.X. Individual biomass model of mature Pinus massoniana plantation, Forestry Prospect and Design, (1), 9-12, 2011 (in Chinese).

47 Huang, D.C. The biomass of Cryptomeria fortunei plantation, Journal of Southwest Forestry College, (1), 23-28, 1986 (in Chinese).

48 Huang, C.B., and Liang, H.W. Growth rhythm and biomass of young Pinus massoniana plantation in the southeast Guangxi, Journal of Guangxi Academy of Sciences, 14(1), 22-27, 1998 (in Chinese).

49 Huang, Z.Z., and Bi, J. Study on the biomass of Robinia pseudoacacia stands in Taihang Mountains, Journal of Hebei Forestry Science and Technology, (2), 48-52, 1992 (in Chinese).

50 Huang, C.H., Zhou, G.Y., Zhao, H.B., Zhou, Z.P., and Qiu, Z.J. Root system biomass of mature Cunninghamia lanceolata plantation in Tianjingshan Forest Farm, Guangdong Province, Journal of Central South University of Forestry and Technology, 33(9), 80-86, 2013 (in Chinese).

51 Huang, S.S., Han, H.R., and Ma, Q.Y. Study on the biomass of broadleaved tree species in Taiyue Forest Region, in: Forum of Forest Ecology, Volume 1, Zhu, Z.H., Luo, J.C., eds., China Agricultural Science and Technology Press, Beijing, China, 96-102, 1999 (in Chinese).

52 Huang, T., Zhong, Q.P., and Peng, X.Y. Study on the biomass and productivity of Liriodendron chinense plantation, Jiangxi Forestry Science and Technology, (5), 4-9, 2000 (in Chinese).

53 Huang, Y.Q., Chen, S.Y., and Wu, X.F. Models for estimating biomass of Eucalyptus urophylla plantation, Journal of Anhui Agricultural University, 28(1), 44-48, 2001 (in Chinese).

54 Hui, G.Y., Tong, S.Z., Liu, J.F., and Luo, Y.W. Effects of afforestation density on the biomass of young Cunninghamia lanceolata plantation, Forest Research, 1(4), 413-417, 1988 (in Chinese).

55 Huo, C.F., You, W.Z., Zhang, H.D., Yan, T.W., Wei, W.J., Zhao, G., Guo, J.S., and Xing, Z.K. Biomass and net primary productivity of Quercus mongolica natural secondary forest in Bingla Mountain, Liaoning Province, Journal of Liaoning Forestry Science and Technology, (4), 4-6, 11, 2011 (in Chinese).

56 Ji, Y.H., Zhang, J.L., and Kang, L.X. A study on biomass equations for Metasequoia glyptostroboides shelterbelt in the coastal agroforestry, Journal of Jiangsu Forestry Science and Technology, 24(2), 1-5, 1997 (in Chinese).

57 Jia, K.X., Zheng, Z., and Zhang, Y.P. Changes in the aboveground biomass of rubber plantations along an elevation gradient in Xishuangbanna, Chinese Journal of Ecology, 25(9), 1028-1032, 2006 (in Chinese).

58 Jia, Y.L., Xu, Z.Q., Ji, X.L., Xu, X.H., and Huang, X.R. Biological carbon storage of a plantation and natural secondary forest in the north region of Yanshan Mountain, Journal of Natural Resources, 27(7), 1241-1251, 2012 (in Chinese).

59 Jiang, Z.L., and Zhao, S. Study on the biomass of Loblolly pine plantation, in: Proceedings of forest ecosystems on Xiashu Ecological Station, Jiang, Z.L., eds., China Forestry Publishing House, Beijing, China, 10-15, 1992 (in Chinese).

60 Jiang, J.P., Yang, X., and Li, R.X. Net productivity and organic matter return of Paulownia plantation ecosystem, Acta Agriculturae Universitatis Henanensis, 23(4), 327-337, 1989 (in Chinese).

61 Jiang, T., Zhao, M., Zhang, S.Z., Yuan, M.L., and Huang, X.R.: Individual biomass and its allocation pattern of Pinus tabuliformis in Hebei, Hebei Journal of Forestry and Orchard Research, 27(3), 239-244, 2012 (in Chinese).

62 Jiang, Z.H., Fan, S.H., Feng, H.X., Zhang, Q., Liu, G.L., and Zong, Y.C. Biomass and distribution pattern of Populus xiaohei plantation in sandy land of north China, Scientia Silvae Sinicae, 43(11), 15-20, 2007 (in Chinese).

63 Jie, J.L., Zhan, Y.S., Huang, W.C., Long, W., Luo, Y.C., Hu, H.Y., and Xie, Z.R. Study on the biomass of Pinus elliottii stand near Jinggangshan Line of Jing-Jiu Railway, Jiangxi Forestry Science and Technology, (2), 17-20, 2002 (in Chinese).

64 Jin, A.L. Study on the biomass of main tree species in Bayingzhuang Forest Farm, Hebei Province, Thesis for Master’s Degree, Beijing Forestry University, Beijing, China, 53pp., 2012 (in Chinese).

65 Jin, A.L., Rao, L.Y., Li, J., and Zhang, T. The biomass of Larix principis-rupprechtii plantation, Guangdong Agricultural Sciences, (12), 165-168, 2012 (in Chinese).

66 Jin, Z.Y., Jia, W.W., and Liu, W. Biomass model for Larix olgensis plantation, Bulletin of Botanical Research, 30(6), 747-752, 2010 (in Chinese).

67 Kong, F.B., and Fang, H. Comparative study on the biomass of Pinus taeda plantations with different densities and ages, Forestry Science and Technology, 28(3), 6-9, 2003 (in Chinese).

68 Li, B.T. Preliminary study on biomass investigation method of Chinese fir plantation, Forest Resources Management, (6), 57-60, 1988 (in Chinese).

69 Li, S.L., and Hou, J.Z. The biomass of Populus tomentosa clonal plantation, Acta Agriculturae Universitatis Henanensis, 29(2), 134-140, 1995 (in Chinese).

70 Li, B.B., Jian, W.H., Qin, Y., Zhang, Y.Z., and Wang, Z.B. Relationship between stand density and the biomass of young Larix principis-rupprechtii plantation, Hebei Journal of Forestry and Orchard Research, 24(3), 244-247, 2009a (in Chinese).

71 Li, H., Li, C.Y., Zha, T.S., Liu, J.L., Jia, X., Wang, X.P., Chen, W.J., and He, G.M. Patterns of biomass allocation in an age-sequence of secondary Pinus bungeana forests in China, The Forestry Chronicle, 90(2), 169-176, 2014.

72 Li, D.L., Jiang, P., and Wang, Y.F. Individual biomass and productivity of Larix principis-rupprechtii plantation in Yanshan Mountain, Hebei Journal of Forestry and Orchard Research, 26(4), 334-339, 2011a (in Chinese).

73 Li, G., Li, Y.G., Liu, M.Z., and Jiang, G.M. Vegetation biomass and net primary production of sparse forest grassland in Hunshandake Sandland, Science and Technology Review, 29(25), 30-37, 2011b (in Chinese).

74 Li, J., Li, H.M., Chen, G.X., and Shi, J.X. A study on the biomass of young Rhus chinensis and R. punjabensis plantation, Journal of Jishou University (Natural Science Edition), 21(1), 1-5, 2000 (in Chinese).

75 Li, J.H., Li, C.J., and Peng, S.K. Study on the biomass expansion factor of poplar plantation, Journal of Nanjing Forestry University (Natural Sciences Edition), 31(4), 37-40, 2007a (in Chinese).

76 Li, N., Xu, W.B., Lai, J.S., Yang, B., Lin, D.M., and Ma, K.P. The coarse root biomass of eight common tree species in subtropical evergreen forest, Chinese Science Bulletin, 58(4), 329-335, 2013 (in Chinese).

77 Li, W.B., Bao, W.K., He, B.H., Wu, W.Y., and Li, F.L. Biomass distribution and its influencing factors of Pinus tabuliformis plantations in the Dagou Valley of the upper Minjiang River, Journal of Mountain Science, 25(2), 236-244, 2007c (in Chinese).

78 Li, X.R., Liu, Q.J., Chen, Y.R., Hu, L.L., and Yang, F.T. Aboveground biomass of three coniferous plantations in Qianyanzhou Research Station, Chinese Journal of Applied Ecology, 17(8), 1382-1388, 2006b (in Chinese).

79 Li, Y., Zhang, J.G., Duan, A.G., and Xiang, C.W. Selection of biomass estimation models for Chinese fir plantation, Chinese Journal of Applied Ecology, 21(12), 3036-3046, 2010a (in Chinese).

80 Li, Y.S., Shi, M.H., and Kang, G.L. The biomass and water-holding capacity of young Pinus tabuliformis plantation, Shanxi Forestry Science and Technology, (4), 35-38, 1994 (in Chinese).

81 Liang, J.P., Zhang, B.X., Yang, H.B., Gu, Y.B., and Zhang, Y.L.: Study on tree biomass of Pinus tabuliformis plantation, Journal of Shanxi Agricultural University, 20(4), 339-341, 2000 (in Chinese).

82 Liang, K.N., Zhou, W.L., and Li, Y.Q. Study on 6-year-old fertilizer trial of Eucalyptus urophylla plantation, Forest Research, 15(6), 644-653, 2002 (in Chinese).

83 Liang, K.N., Zhou, W.L., and Li, Y.Q. Effects of fertilization on biomass and nutrient contents of young Eucalyptus urophylla cl. MLA plantation, Forest Research, 17(3), 327-333, 2004 (in Chinese).

84 Liang, N., Wang, W.B., and Tian, K. Biomass distribution characteristics of 4- and 13-year-old Betula alnoides plantations, Journal of West China Forestry Science, 35(4), 188-192, 2006 (in Chinese).

85 Liao, H.Z., Zheng, Y.M., Zhang, C.N., and Chen, D.Y. Study on the biomass of Cinnamomum camphora plantation, Forest Science and Technology, (9), 15-18, 1986 (in Chinese).

86 Lin, K.M., Zheng, Y.S., Huang, Z.Q., and Wu, Z.X. Study on the models for biomass of young Chinese fir and Masson pine plantation, Journal of Fujian College of Forestry, 13(4), 351-356, 1993 (in Chinese).

87 Lin, S.M., Xu, T.G., and Zhou, G.M. Study on the biomass of Chinese fir plantation, Journal of Zhejiang Forestry College, 8(3), 288-294, 1991 (in Chinese).

88 Ling, L. Growth rules of individual biomass of Populus wenxianica forest, Protection Forest Science and Technology, (3), 9-11, 2011 (in Chinese).

89 Liu, B. Study on individual biomass and allocation pattern of Pinus tabuliformis natural forest in Helan Mountains, China, Thesis for Master’s Degree, Northwest Agricultural and Forestry University, Yangling, China, 46pp., 2010 (in Chinese).

90 Liu, W.Y. Study on the biomass and productivity of Acacia dealbata plantation in the protected district of water sources in north Kunming, Guihaia, 15(4), 327-334, 1995b (in Chinese).

91 Liu, Z.G., and Ma, Q.Y. An approach to methods for estimating biomass of Larix principis-rupprechtii artificial forests, Journal of Beijing Forestry University, 14(S), 105-113, 1992 (in Chinese).

92 Liu, S.R., Chai, Y.X., Cai, T.J., and Peng, C.H. Study on the biomass and net primary productivity of Dahurian larch plantation, Journal of Northeast Forestry University, 18(2), 40-45, 1990 (in Chinese).

93 Liu, Y.B., Zhang, Y.L., Zhao, T.S., Li, Z.L., Shi, Z.X., Li, Z.A., Zhang, Y.L., Meng, X.T., Hao, Z.Y., Mao, X.Q., Zhang, Y.S., and Dong, C.Y. Biomass and productivity of the agroforestry ecosystems, Journal of Henan Agricultural College, (1), 13-20, 1984 (in Chinese).

94 Liu, Y.C., Jiang, Y.B., Chen, H.W., and Li, J. Regression equations for individual tree of Betula alnoides plantation, Journal of Fujian Forestry Science and Technology, 35(2), 42-46, 2008 (in Chinese).

95 Liu, Z.Q., Chen, G.H., Meng, Y.Q., Li, J.G., and Liu, M.R. Biomass and nutrient storage of Larix principis-rupprechtii plantation, Forest Research, 8(1), 88-93, 1995b (in Chinese).

96 Long, S.M., and Wang, Y. Biomass and economic benefits of Casuarina equisetifolia plantation, Central South Forest Inventory and Planning, (2), 25-34, 1985 (in Chinese).

97 Lu, Q., Li, Z.J., and Li, X.D. The models of biological productivity of Castanopsis fargesii forest, Journal of Guangxi Agricultural College, 9(3), 55-64, 1990 (in Chinese).

98 Luan, K.Z., and Liu, Z.G. Biomass estimation models for Pinus sylvestris var. mongolica plantation, Forestry Science and Technology, 37(3), 35-38, 2012 (in Chinese).

99 Luo, Y.J., Zhang, X.Q., Wang, X.K., Zhu, J.H., Zhang, Z.J., Sun, G.S., and Gao, F. Biomass and its distribution patterns of Larix principis-rupprechtii plantations in northern China, Journal of Beijing Forestry University, 31(1), 13-18, 2009 (in Chinese).

100 Lyu, X.T., Tang, J.W., He, Y.C., Duan, W.G., Song, J.P., Xu, H.L., and Zhu, S.Z. Biomass and its allocation in tropical seasonal rain forest in Xishuangbanna, southwest China, Chinese Journal of Plant Ecology, 31(1), 11-22, 2007.

101 Ma, Q.Y. A study on the biomass and primary productivity of Chinese pine (Pinus tabuliformis Carr.) forests, PhD dissertation, Beijing Forestry University, Beijing, China, 178pp., 1988 (in Chinese).

102 Ma, Q.Y. A study on the biomass of Chinese pine forests, Journal of Beijing Forestry University, 11(4), 1-10, 1989 (in Chinese).

103 Ma, X.X., and Li, W.J. Biomass table of Korean pine natural forest in northern Changbai Mountains, Forest Investigation Design, (3), 74-75, 2008 (in Chinese).

104 Mei, L. Fine root turnover and carbon allocation in Manchurian ash and Dahurian larch plantations, PhD Dissertation, Northeast Forestry University, Harbin, China, 102pp., 2006 (in Chinese).

105 Mei, L., Zhang, Z.W., Gu, J.C., Quan, X.K., Yang, L.J., and Huang, D. Carbon and nitrogen storages and allocation in tree layers of Fraxinus mandshurica and Larix gmelinii plantations, Chinese Journal of Applied Ecology, 20(8), 1791-1796, 2009 (in Chinese).

106 Meng, L., Cheng, J.M., Yang, X.M., Han, J.J., Fan, W.J., and Hu, X.J. Carbon storage and density of Pinus tabuliformis plantation in Ziwuling Forest Region of the Loess Plateau, Bulletin of Soil and Water Conservation, 30(2), 133-137, 2010 (in Chinese).

107 Mo, D.X. Study on carbon and nitrogen pattern and ecosystem biomass of Cryptomeria fortunei plantation of different density in southeastern Guangxi Province, Thesis for Master’s Degree, Guangxi University, Nanjing, China, 52pp., 2013 (in Chinese).

108 Qin, S.J., Li, K., Mo, D.X., and Wu, Q.B. Biomass regression model of Cryptomeria fortunei plantation in southeast Guangxi, Journal of Southern Agriculture, 44(2), 261-265, 2013 (in Chinese).

109 Mu, T.M. Study on the biomass of Picea crassifolia forest in Helan Mountains, Inner Mongolia Forestry Science and Technology, (1), 34-45, 1981 (in Chinese).

110 Mu, C.C., Wan, S.C., Su, P., Song, H.W., and Zun, Z.H. Biomass distribution patterns of Alnus hirsuta and Betula platyphylla-swamp ecotone communities in Changbai Mountains, Chinese Journal of Applied Ecology, 15(12), 2211-2216, 2004 (in Chinese).

111 Mu, C.C., Wang, B., Lu, H.C., Bao, X., and Cui, W. Carbon storage of natural wetland ecosystem in Daxing’anling of China, Acta Ecologica Sinica, 33(16), 4956-4965, 2013 (in Chinese).

112 Pan, K.W., and Liu, Z.G. The biomass of 10-year-old Cercidiphyllum japonicum plantation, Chinese Journal of Applied and Environmental Biology, 5(2), 121-130, 1999 (in Chinese).

113 Pan, F.J., Zhang, Z.F., Huang, Y.Q., and Mo, L. Aboveground biomass of Cyclobalanopsis glauca analyzed by tree-ring method in karst region, Guihaia, 32(4), 464-467, 2012 (in Chinese).

114 Pang, J.P. Carbon storage and its allocation of rubber plantation in Xishuangbanna, southwest China, Thesis for Master’s Degree, Xishuangbanna Tropical Botanical Garden, Chinese Academy of Sciences, Xishuangbanna, China, 54pp., 2009 (in Chinese).

115 Tang, J.W., Pang, J.P., Chen, M.Y., Guo, X.M., and Zeng, R. Biomass and its estimation model of rubber plantations in Xishuangbanna, southwest China, Chinese Journal of Ecology, 28(10), 1942-1948, 2009 (in Chinese).

116 Peng, H.C. Biomass measurement of Eucommia ulmoides plantation, Nonwood Forest Research, 10(2), 28-33, 1992 (in Chinese).

117 Qi, J.F., and Tang, J.W. Biomass and its allocation pattern of limestone monsoon rain forest in Xishuangbanna, Chinese Journal of Ecology, 27(2), 167-177, 2008 (in Chinese).

118 Qi, G., Wang, Q.L., Wang, X.C., Qi, L., Wang, Q.W., Ye, Y.J., and Dai, L.M. Vegetation carbon storage in Larix gmelinii plantations in Daxing’an Mountains, Chinese Journal of Applied Ecology, 22(2), 273-279, 2011.

119 Qian, G.Q. Dynamics of the net primary productivity of Liquidambar formosana plantation, Acta Agriculturae Universitatis Jiangxiensis, 22(3), 399-404, 2000 (in Chinese).

120 Qin, Z.G. Community features and the biomass of Alnus cremastogyne fuelwood forest, Journal of Sichuan Forestry Science and Technology, 13(1), 24-28, 33, 1992 (in Chinese).

121 Qin, W.M., Qiu, B.F., Qin, J., Xu, T.B., and Qin, D.W. The biomass and growth rhythm of Paramichelia baillonii plantation, Journal of Fujian College of Forestry, 31(2), 110-114, 2011c (in Chinese).

122 Qiu, Y., Zhang, J.T., Chai, B.F., and Zheng, F.Y. The aboveground biomass and productivity of Pinus tabuliformis planted forest in the west of Shanxi Province, Henan Science, 17(S), 72-76, 79, 1999 (in Chinese).

123 Shen, Z.K., Lu, S.P., and Ai, X.R. The biomass and productivity of Larix kaempferi plantation, Journal of Hubei Institute for Nationalities (Natural Science Edition), 23(3), 289-292, 2005 (in Chinese).

124 Shi, Y.L. Study on the ecosystem biomass of the artificial Cunninghamia lanceolata forest in Changling (Wanli District), Nanchang City, Acta Agriculturae Universitatis Jiangxiensis, 11(4), 32-45, 1989 (in Chinese).

125 Shi, Z., Liu, J., Lan, X., Liang, D., Lu, M.B., and Deng, M.Y. Individual biomass regression models of Pinus massoniana plantation in Tianlin County, Guangxi Province, Guangxi Forestry Science, 38(3), 167-170, 2009 (in Chinese).

126 Su, Y., Qin, W.M., Huang, S.D., Duan, W.W., Wei, Z.M. The biomass and productivity of Parashorea chinensis plantation in the southwest Guangxi, Practical Forestry Technology, (9), 16-19, 2011 (in Chinese).

127 Sun, B.G., Chen, F., Wang, J.M., Chen, X.M., Yang, Z.X., Cai, X.Y., and Li, B. Biomass distribution pattern of Pinus yunnanensis with different diameter classes, Forest Research, 25(1), 71-76, 2012a (in Chinese)

128 Sun, D., Ruan, H.H., and Ye, J.Z. The biomass structure of the secondary natural oakery in Kongqing Hill, in: Proceedings of forest ecosystems on Xiashu Ecological Station, Jiang, Z.L., eds., China Forestry Publishing House, Beijing, China, 16-22, 1992 (in Chinese).

129 Sun, N., Li, Y.Z., and Zhang, Y.C. Influence of afforestation density on the biomass of hybrid larch, Forestry Science and Technology, 37(4), 14-16, 2012b (in Chinese).

130 Sun, Q.X., Yu, F.A., and Peng, Z.H. The biomass of poplar plantation in the beach land of Yangtze River, Forest Science and Technology, (3), 4-6, 1998 (in Chinese).

131 Wu, Z.M., Sun, Q.X., and Duan, W.X. Relationship between flooded situation and poplar growth on beach land of Yangtze river in Anhui, Chinese Journal of Applied Ecology, 11(1), 25-29, 2000 (in Chinese).

132 Tian, D.L., Pan, W.C., Lei, Z.X., Long, E.H., and Cai, B.Y. A preliminary study on the biomass and density effects of Pinus massoniana plantation, Journal of Central South Forestry Institute, 2(1), 41-50, 1982 (in Chinese).

133 Wan, S.C. The biomass allocation patterns of constructive tree species in forest-swamp ecotone, Journal of Mudanjiang University, 22(8), 124-127, 2013 (in Chinese).

134 Wan, M., Tian, D.L., Fan, W., and Li, Q.Y. Biomass production and carbon sequestration in poplar-crop agroforestry ecosystems in eastern Henan Plain, Scientia Silvae Sinicae, 45(8), 27-33, 2009 (in Chinese).

135 Wang, L.M. Biomass determination of stem, branch and needle of Pinus sylvestris var. mongolica natural forest, Journal of Inner Mongolia Forestry College, (2), 63-68, 1986 (in Chinese).

136 Wang, M.B. The biomass of Populus hopeiensis forest, Journal of Shanxi University (Natural Science Edition), 14(1), 103-107, 1991 (in Chinese).

137 Wang, X.Y. Carbon storage distribution of Larix olgensis plantation at different stand ages, PhD Dissertation, Beijing Forestry University, Beijing, China, 96pp., 2011 (in Chinese).

138 Wang, J., and Wen, Z.W. Aboveground biomass distribution and predictive model of secondary Betula luminifera forest in the northwest Guizhou Province, Guizhou Forestry Science and Technology, 39(2), 18-21, 2011 (in Chinese).

139 Wang, Y.M., and Liu, B.Z. Ecological Characteristics of Protection Forests in the Loess Plateau, China Forestry Publishing House, Beijing, China, 42-48, 1994 (in Chinese).

140 Wang, Z.F., and Feng, Z.K. The parameter estimation of tree biomass using the nonlinear least square method, Journal of Jilin Agricultural University, 28(3), 261-264, 2006 (in Chinese).

141 Wang, C., Gao, H.Z., and Zang, Y.Q. Study on the individual biomass of Betula platyphylla natural secondary forest, Forestry Science and Technology, 35(1), 7-9, 13, 2010 (in Chinese).

142 Wang, D.Y., Li, D.Y., and Feng, X.Q. Forest Ecosystems on Warm Temperate Zone, China Forestry Publishing House, Beijing, China, 190-205, 2003b (in Chinese).

143 Wang, J.S., Zhang, C.Y., Fan, X.H., and Zhao, Y.Z. Biomass allocation patterns and allometric models of Abies nephrolepis, Acta Ecologica Sinica, 31(14), 3918-3927, 2011a (in Chinese).

144 Wang, J.Y., Che, K.J., Fu, H.E., Chang, X.X., Song, C.F., and He, H.Y. Study on the biomass of water conservation forest on the north slope of Qilian Mountains, Journal of Fujian College of Forestry, 18(4), 319-323, 1998 (in Chinese).

145 Wang, N., Wang, B.T., Wang, R.J., Cao, X.Y., Wang, W.J., and Chi, L. Biomass allocation patterns and allometric models of Populus davidiana and Pinus tabuliformis in the west Shanxi Province, Bulletin of Soil and Water Conservation, 33(2), 151-155, 159, 2013 (in Chinese).

146 Wang, X.W., Li, X.Y., Guan, X.D., and Bai, G.X. Preliminary study on the biomass of Larix olgensis plantation, Journal of Liaoning Forestry Science and Technology, (6), 30-34, 1993 (in Chinese).

147 Wang, X.Y., Hu, D., and He, J.S. Study on the biomass of Fagus engleriana forest and Quercus aliena var. acuteserrata forest in Shennongjia Region, Journal of Capital Normal University (Natural Science Edition), 28(2), 62-67, 2007a (in Chinese).

148 Wang, Y.T., Ma, Q.Y., Hou, G.W., Kan, Z.G., and Chen, Y. Dynamics of the biomass and productivity of the naturally-regenerated Pinus densata forest in the burned areas of the western Sichuan Province, Forestry Science and Technology, 32(1), 37-40, 2007b (in Chinese).

149 Wang, Z., Han, Y.J., Kang, H.Z., Huang, D., Xue, C.Y., Yin, S., and Liu, C.J. Carbon storage of main water conservation plantations in the upper reaches of Huangpu River, Shanghai, Chinese Journal of Ecology, 31(8), 1930-1035, 2012c (in Chinese).

150 Wei, Y.J. Study on the biomass of Fokienia hodginsii plantations in south Fujian, Journal of Fujian Forestry Science and Technology, 28(3), 21-23, 38, 2001 (in Chinese).

151 Wen, D.Z., Wei, P., Kong, G.H., Zhang, Q.M., and Huang, Z.L. Biomass study of the Castanopsis chinensis + Cryptocarya concinna + Schima superba community in Dinghushan Biosphere Reserve, Acta Ecologica Sinica, 17(5), 497-504, 1997 (in Chinese).

152 Wen, D.Z., Wei, P., Zhang, Q.M., and Kong, G.H. Study on the biomass of three subtropical evergreen broadleaved forests in Dinghushan Biosphere Reserve, Acta Phytoecologica Sinica, 23(S), 11-21, 1999 (in Chinese).

153 Wu, X.C. Study on the productivity and carbon density of poplar-willow natural forest in Ergis River, Xinjiang, PhD Dissertation, Inner Mongolia Agricultural University, Huhhot, China, 109pp., 2009 (in Chinese).

154 Wu, X.C., Zhang, Q.L., Lei, Q.Z., and Bai, Z.Q. Biomass distribution characteristics of natural forests in Ergis River, Xinjiang, Forest Resources Management, (4), 61-67, 2009 (in Chinese).

155 Wu, Z.L., and Dang, C.L. Preliminary study on the biomass and net primary productivity of Quercus senescens forest near Kunming, Journal of Yunnan University (Natural Science Edition), 16(3), 235-239, 244, 1994 (in Chinese).

156 Wu, G., Feng, Z.W., Kong, H.M., and Qin, Y.Z. The biomass and productivity of agroforestry ecosystems in northern Henan Province, Research of Agricultural Modernization, 14(2), 99-102, 1993 (in Chinese).

157 Wu, M., Li, S.T., Qin, W.M., and Qin, X.L. The biomass and productivity of 11-year-old Michelia hedyosperma plantation, Journal of West China Forestry Science, 42(4), 46-51, 2013 (in Chinese).

158 Wu, P., Ding, F.J., Cui, Y.C., Zhu, J., and Li, C.R. The biomass and productivity of young Pinus massoniana forests of Pearl River Shelterbelt Construction Project in Qiannan City, Guizhou Agricultural Sciences, 40(6), 169-172, 2012a (in Chinese).

159 Wu, S.R., Yang, H.Q., Hong, R., Zhu, W., and Chen, X.Q. The biomass and its distribution of Pinus massoniana plantation, Journal of Fujian Forestry Science and Technology, 26(1), 18-21, 1999 (in Chinese).

160 Wu, Z.L., Dang, C.L., He, Z.R., and Wang, C.Y. Preliminary study on the biomass of Picea brachytla var. complanata forests in the northwest Yunnan Province, Journal of Yunnan University (Natural Science Edition), 16(3), 230-234, 1994b (in Chinese).

161 Wu, Z.L., Dang, C.L., Wang, C.Y., and He, Z.R. Preliminary study on the biomass of Pinus densata forests in the northwest Yunnan Province, Journal of Yunnan University (Natural Science Edition), 16(3), 220-224, 1994c (in Chinese).

162 Wu, Z.M., Sun, Q.X., and Chen, M.G. Biomass and nutrient accumulation of poplar plantation in the beach land of Yangtze River in Anhui Province, Chinese Journal of Applied Ecology, 12(6), 806-810, 2001 (in Chinese).

163 Xiao, Y. Comparative study on the biomass and productivity of Pinus tabuliformis plantations in different climatic zones, Shaanxi Province, Acta Phytoecologica et Geobotanica Sinica, 14(3), 237-246, 1990 (in Chinese).

164 Xiao, Y. Biomass and productivity of natural Pinus henryi forest, Acta Phytoecologica et Geobotanica Sinica, 16(3), 227-233, 1992 (in Chinese).

165 Xiao, Y.T., He, A.H., and Liang, J.Z. Community characteristics and the biomass of Pinus taiwanensis natural forest in Mufu Mountain of Hubei-Hunan border, Hunan Forestry Science and Technology, (4), 1-4, 1989 (in Chinese).

166 Xiao, Z.W., Wang, L.J., Mao, J.M., Zhu, X.Z., Wang, X.L., Zheng, L., and Tang, J.W. Carbon storage of different tree-tea agroforestry systems in Xishuangbanna, Yunnan Province of southwest China, Chinese Journal of Ecology, 31(7), 1617-1625, 2012 (in Chinese).

167 Xie, Z.S., Chen, B.G., Han, J.G., and Deng, Y.S. The biomass estimative model of two Eucalypts in Leizhou Peninsula, in: Studies on the Eucalypt Ecosystem of Short Rotation in Leizhou, Zeng, T.X., eds., China Forestry Publishing House, Beijing, China, 66-75, 1995 (in Chinese).

168 Xu, D.P., Zeng, Y.T., and Li, W.X. Aboveground biomass and nutrient cycling of young Eucalyptus urophylla plantation, Forest Research, 7(6), 600-605, 1994b (in Chinese).

169 Xu, D.P., He, Q.X., Yang, Z.J., Long, Y.S., and Jian, X.H. Aboveground primary productivity and nutrient cycling of Eucalyptus grandis × E. urophylla plantation, Forest Research, 10(4), 365-372, 1997 (in Chinese).

170 Xu, D.P., Bernie, D., Gong, M.Q., Nick, M., and Wang, Z.H. Effects of phosphorus fertilization and ectomycorrhizal fungal inoculation on the productivity and nutrient accumulation of Eucalyptus globulus plantation, Forest Research, 17(1), 26-35, 2004 (in Chinese).

171 Xu, H.Y., Zheng, S.K., and Lu, Y.N. The biomass of I-72/58 poplar plantation, Scientia Silvae Sinicae, 26(1), 22-29, 1990 (in Chinese).

172 Xu, J.W., Li, C.R., Wang, W.D., Qiao, Y.J., Cheng, H.Y., and Wang, Y.H. The biomass and productivity of Pinus thunbergii protective forests in sandy coastal area, Journal of Northeast Forestry University, 33(6), 29-32, 2005 (in Chinese).

173 Xu, W., Hu, H.B., and Zhou, C.H. Biomass structure and distribution characteristics of Pinus massoniana plantation in eastern Anhui Province, Journal of Central South University of Forestry and Technology, 31(6), 111-115, 2011b (in Chinese).

174 Xu, Z.B., Li, X., Dai, H.C., Tan, Z.X., Zhang, Y.P., Guo, X.F., Peng, Y.S., and Dai, L.M. Study on the biomass of the broadleaved Korean pine forest in Changbai Mountain, Research of Forest Ecosystem, 5, 33-47, 1985 (in Chinese).

175 Xu, Z.Q., Li, W.H., Liu, W.Z., and Wu, X.B. Study on the biomass and productivity of Mongolian oak forests in northeast region of China, Chinese Journal of Eco-Agriculture, 14(3), 21-24, 2006 (in Chinese).

176 Xu, Z.Z., Liu, G.Y., Wang, G.H., Zhao, G.H., and Ma, C.M. The biodiversity and biomass in Larix principis-rupprechtii plantation community in Yanshan Mountains, Forest Resources Management, (2), 43-49, 2010 (in Chinese).

177 Xuan, Z.L., Zhang, Q.C., Ge, L.L., He, H.J., Xu, M.M., and Xu, W.S. Biomass structure and distribution of Korean larch plantations, Forest Resources Management, (1), 53-57, 2013 (in Chinese).

178 Xue, X.K., and Sheng, W.T. Study on the biomass of Fokienia hodginsii plantation in Zhuting Town, Hunan Province, Forest Science and Technology, (4), 16-19, 1993 (in Chinese).

179 Xue, L., Jacobs, D.F., Zeng, S.C., Yang, Z.Y., Guo, S.H., and Liu, B.: Relationship between aboveground biomass and stand density index in Populus × euramericana stands, Forestry, 85, 611-619, 2012.

180 Yan, J.F., Guan, Q.W., Deng, S.Q., Yu, S.Q., and Shan, X.D. The biomass and productivity of Platycladus orientalis plantation in Yunlong Mountain of Xuzhou, China Forestry Science and Technology, 23(2), 48-50, 2009 (in Chinese).

181 Yang, X. The biomass and productivity of the intercropping ecosystem with paulownia (Paulownia elongata) and crops, Acta Agriculturae Universitatis Henanensis, 20(4), 485-509, 1986 (in Chinese).

182 Yang, X., Wu, G., Huang, D.M., and Yang, C.Q. Quantitative study on biomass accumulation of Paulownia elongate, Chinese Journal of Applied Ecology, 10(2), 143-146, 1999 (in Chinese).

183 Yang, Z.W., Tan, F.L., Xiao, X.X., Chen, L.S., and Zhuo, K.F. Study on the biomass of Fokienia hodginsii plantation, Scientia Silvae Sinicae, 36(S1), 120-124, 2000 (in Chinese).

184 Yao, Y.J., Kang, W.X., and Tian, D.L. Study on the biomass and productivity of Cinnamomum camphora plantation, Journal of Central South Forestry University, 23(1), 1-5, 2003 (in Chinese).

185 Ye, W.H. Study on Tree Architecture of Three Hardwoods. Heilongjiang Science and Technology Press, Harbin, China, 84-88, 1995 (in Chinese).

186 Ye, W.P. Structure dynamics and vegetation productivity in the processes of ecological restoration in red earth of Lijiang Valley, Thesis for Master’s Degree, Guangxi Normal University, Guilin, China, 41pp., 2005 (in Chinese).

187 Ye, W.P., Li, X.K., Lyu, S.H., Ou, Z.L., Pan, Z., Su, Z.M., and Xie, X. Dynamics of vegetation structure and biomass in the processes of ecological restoration in the red soil region of Lijiang Valley, Journal of Ecology and Rural Environment, 22(1), 5-10, 2006 (in Chinese).

188 Ye, J.Z., and Jiang, Z.L. Study on the biomass and its distribution of Cunninghamia lanceolata plantations in the hilly regions of the south Jiangsu Province, Acta Ecologica Sinica, 3(1), 7-14, 1983 (in Chinese).

189 Ye, J.Z., Jiang, Z.L., Zhou, B.L., Han, F.Q., and Chen, S.B. Annual dynamics of the biomass of Chinese fir forests in Yangkou Forest Farm, Fujian Province, Journal of Nanjing Institute of Forestry, (4), 1-9, 1984 (in Chinese).

190 You, W.Z., Huo, C.F., Xing, Z.K., Zhao, G., Zhang, H.D., Wei, W.J., and Yan, T.W. Biomass and net primary productivity of Larix olgensis plantation in Bingla Mountains, northeast China, Journal of Shenyang Agricultural University, 42(5), 565-569, 2011 (in Chinese).

191 Yu, B., Zhang, Q.L., Wang, L.M., and Wu, J Characteristics of biomass and productivity in Larix gmelinii natural forests with different stand structures, Journal of Zhejiang Agriculture and Forestry University, 28(1), 52-58, 2011 (in Chinese).

192 Yu, Y.F., Song, T.Q., Zeng, F.P., Peng, W.X., Wen, Y.G., Huang, C.B., Wu, Q.B., Zeng, S.X., and Yu, Y. Dynamic changes of biomass and its allocation in Cunninghamia lanceolata plantations of different stand ages, Chinese Journal of Ecology, 32(7):1660-1666, 2013 (in Chinese).

193 Zeng, X.P., Cai, X.A., Zhao, P., Rao, X.Q., Zou, B., Zhou, L.X., Lin, Y.B., and Fu, S.L. Biomass and net primary productivity of three plantation communities in hilly land of lower subtropical China, Journal of Beijing Forestry University, 30(6), 148-152, 2008 (in Chinese).

194 Zhang, B.L. Study on the biomass and productivity of Quercus liaotungensis stands in Ziwuling Forest Region, Shaanxi Province, Journal of Northwestern College of Forestry, 5(1), 1-7, 1990 (in Chinese).

195 Zhang, H.T. Preliminary study on the biomass of Pinus sylvestris var. sylvestriformis forest, Jilin Forestry Science and Technology, (3), 5-7, 1992 (in Chinese).

196 Zhang, Z.J. Study on spatial characteristics of Pinus massoniana biomass and root distribution in an acid rain area, Chongqing, Thesis for Master’s Degree, Agricultural University of Hebei, Baoding, China, 50pp., 2006 (in Chinese).

197 Zhang, Z.J., Wang, Y.H., Yuan, Y.X., Li, Z.Y., Cao, L., Zhang, G.Z., Yu, P.T., and Wang, Y. Study on the biomass and distribution of Pinus massoniana natural secondary forest, Journal of Agricultural University of Hebei, 29(5), 37-43, 2006 (in Chinese).

198 Zhang, B.L., and Chen, C.G. Biomass and productivity of Robinia pseudoacacia plantation in Hongxing Forest Farm of Changwu County, Shaanxi Province, Shaanxi Forest Science and Technology, (3), 13-17, 1992 (in Chinese).

199 Zhang, G.B., Li, X.Q., She, X.S., Hu, C.Q., and Hu, G.H. Biomass characteristics of dominant tree species (group) in Lingnan Forest Farm, Anhui Province, Scientia Silvae Sinicae, 48(5), 136-140, 2012 (in Chinese).

200 Zhang, Q., Fan, S.H., Liu, G.L., Feng, H.X., Zong, Y.C., and Fei, B.H. A study on biomass and productivity of Populus × euramericana cv. ‘San Martino’ (I-72/58) plantation on beach land of Yangtze River, Forest Research, 21(4), 542-547, 2008a (in Chinese).

201 Zhang, S.G., Liu, J., Huang, K.Y., Liang, R.L., and Lan, X. Biomass and distribution patterns of Pinus massoniana plantation in northwest Guangxi, Guangxi Forestry Science, 39(4), 189-192, 219, 2010 (in Chinese).

202 Zhang, W.Y., Zheng, Y.S., and You, X.Z. A study on the biomass models for organs of Tsoongiodenron odorum, Acta Agriculturae Universitatis Jiangxiensis, 21(3), 410-413, 1999b (in Chinese).

203 Zhang, Z.H., Li, Y., and Xie, R.G. A preliminary study on the growth and biomass of the mixed plantation of Magnolia officinalis and Cunninghamia lanceolata, Journal of Fujian Forestry Science and Technology, 23(3), 28-31, 1996b (in Chinese).

204 Zhang, Z.H., Wang, L.C., Luo, J.X., and Zheng, D.R. Study on tree biomass models of Pinus yunnanensis in northwest Yunnan Province, Shandong Forestry Science and Technology, (4), 4-6, 2011 (in Chinese).

205 Zhao, T.S., and Zhang, P.C. Comprehensive effects of tending and felling on Pinus taiwanensis plantation, Acta Agriculturae Universitatis Henanensis, 23(4), 409-421, 1989 (in Chinese).

206 Zhao, J.M., Wu, Z.W., and Xie, S.X. Individual growth and biomass characteristics of wild Idesia polycarpa in Guizhou, Guizhou Forestry Science and Technology, 40(4), 7-13, 23, 2012 (in Chinese).

207 Zheng, H., Ouyang, Z.Y., Xu, W.H., Wang, X.K., Miao, H., Li, X.Q., and Tian, Y.X. Variation of carbon storage by different reforestation types in the hilly red soil region of southern China, Forest Ecology and Management, 255, 1113-1121, 2008.

208 Zheng, Z., Feng, Z.L., Cao, M., Liu, H.M., and Liu, L.H. Biomass and net primary productivity of primary tropical wet seasonal rainforest in Xishuangbanna, Acta Phytoecologica Sinica, 24(2), 197-203, 2000 (in Chinese).

209 Zhou, S.Q., and Huang, J.Y. Biomass estimation models of Larix mastersiana plantation, Journal of Sichuan Forestry Science and Technology, 12(2), 67-69, 1991a (in Chinese).

210 Zhou, S.Q., and Huang, J.Y. A study on biomass and productivity of Larix mastersiana plantation in Sichuan, Acta Phytoecologica et Geobotanica Sinica, 15(1): 9-16, 1991b (in Chinese).

211 Zhou, G.M., Yao, J.X., Qiao, W.Y., Yang, Q.H., Zhu, G.J., and Xu, W.Y. Biomass of Chinese fir planted forest in Qingyuan County of Zhejiang Province, Journal of Zhejiang Forestry College, 13(3), 235-242, 1996 (in Chinese).

212 Zhou, G.Y., Zeng, Q.B., Lin, M.X., Chen, B.F., Li, Y.D., and Wu, Z.M. Study on the biomass and nutrient allocation in Manglietia hainanensis plantation ecosystem at Jianfengling, Hainan Province, Forest Research, 10(5), 453-457, 1997 (in Chinese).

213 Zhou, W.C., Mu, C.C., Liu, X., and Gu, W. Carbon sink in natural swamp forest ecosystems in Xiaoxing’an Mountains, Journal of Northeast Forestry University, 40(7), 71-75, 127, 2012 (in Chinese).

214 Zhou, Z.Z., Zheng, H.S., Yin, G.T., Yang, Z.J., and Chen, K.T. Biomass equations for rubber tree in southern China, Forest Research, 8(6), 624-629, 1995 (in Chinese).

215 Zhuang, H.L., Becuwe, X., Xiao, C.B., Wang, Y.H., Wang, H., Yin, B., and Liu, C.J. Allometric equation-based estimation of biomass carbon sequestration in Metasequoia glyptostroboides plantations in Chongming Island, Shanghai, Journal of Shanghai Jiaotong University (Agricultural Science Edition), 29(2), 48-55, 2012 (in Chinese).

216 Zou, C.J., Bu, J., and Xu, W.D. Biomass and productivity of Pinus sylvestriformis plantation, Chinese Journal of Applied Ecology, 6(2), 123-127, 1995 (in Chinese).

217 Yin, Y., Zeng, W., Tang, S. Modeling of Standing Tree Biomass for Larix in Northeast China[J]. Journal of Northeast Forestry University, 38(9), 23-24, 2010 (in Chinese).

218 Snell, J.A. Kendall and Little, S.N. Predicting crown weight and bole volume of live western hardwoods. U.S. For. Serv. Gen. Tech. Rep. PNW151, 37, 1983.

219 Young, H.E., Ribe, J.H. and Wainwright, K. Weight tables for tree and shrub species in Maine. Life Sciences and Agri- culture Experiment Station, University of Maine at Drone, Miscellaneous Report 230. 84 pp, 1980.

220 Perala, D.A. and Alban, D.H. Allometric biomass estima- tors for aspen-dominated ecosystems in the Upper Great Lakes. U.S. For. Serv. Res. Pap. NC-134, 38, 1994.

221 Brenneman, B.B.. Frederick, D.J.. Gardner, WE., Schoenhofen, L.H. and Marsh, P.L. Biomass of species and stands of West Virginia hardwoods. In: P.E. Pope (Editor). Proceedings of Central Hardwood Forest Conference II. West LaFayette, Purdue University, pp. 159-178, 1978.

222 Ker. M.F. Tree biomass equations for seven species in southwestern New Brunswick. Can. For. Serv. Marit. For. Res. Cent. Inf. Rep., M-X-114, 18, 1980a.

223 Freedman, B., Duinker, P.N., Barclay. H., Morash, R. and Pragcr, U. Forest biomass and nutrient studies in central Nova Scotia. Can. For. Serv. Marit. For. Res. Cent. Inf. Rep., M-X-134, 126, 1982.

224 Campbell, J.S., Lieffers, V.J. and Pielou, E.C. Regression equations for estimating single tree biomass of trembling aspen: assessing their applicability to more than one popuia- tion. For. Ecol. Manage., 11, 283-295, 1985.

225 Johnston, R.S. and Bartos, D.L. Summary of nutrient and biomass data from two aspen sites in western United States. U.S. For. Serv. Res. Note INT-227, 15, 1977.

226 Ker, M.F. Tree biomass equations for ten major species in Cumberland County, Nova Scotia. Can. For. Serv. Marit. For. Res. Cent. Inf. Rep., M-X-108, 26, 1980b.

227 Ker, M.F. Biomass equations for seven major maritimes tree species. Can. For. Serv. Marit. For. Res. Cent. Inf. Rep., M-X- 148, 54, 1984.

228 MacLean, D.A. and Wein, R.W. Biomass of jack pine and mixed hardwood stands in northeastern New Brunswick. Can. J. For. Res., 6, 441-447, 1976.

229 Pastor, J. and Bockheim, J.G. Biomass and production of an aspen-mixed hardwood-spodosol ecosystem in northern Wisconsin. Can. J. For. Res., 11, 132-138, 1981.

230 Peterson, E.B., Chan, Y.B. and Cragg, J.B. Aboveground standing crop, leaf area, and caloric value in an aspen clone near Calgary, Alberta. Can. J. Bot., 48, 1459-1469, 1970.

231 Whittaker, R.H., Bormann, F.H., Likens, G.E. and Siccama, T.G. The Hubbard Brook ecosystem: Forest biomass and production. Ecol. Monogr., 44, 233-252, 1974.

232 Baskerville, G.L. Dry-matter production in immature bal- sam fir stands. Forest Science Monograph, Society of Ameri- can Foresters, Washington, DC, 41 pp, 1965.

233 Krumlik, J.G. Biomass and nutrient distribution in two old growth forest ecosystems in south coastal British Columbia. MSc. Thesis, University of British Columbia, Vancouver, British Columbia, 180 pp, 1974.

234 Wiant, H.V., Jr.. Sheetz, C.E., Colaninno. A., DeMoss, J.C. and Castaneda, F. Tables and procedures for estimating weights of some Appalachian hardwoods. W. Va, Agric. Exp Stn. Bull., 659(T), 36, 1977.

235 Honer, T.G. Weight relationships in open- and forest-grown balsam fir trees. In: Forest Biomass Studies. IUFRO Section 25, Yield and Growth, Working Group on Forest Biomass Studies, Life Sciences and Agriculture, Experimental Station, University of Maine at Orono, Miscellaneous Publication No. 132, pp, 65-78, 1971.

236 Carpenter, E.M. Above-ground weights for tamarack m northeastern Minnesota. U.S. For. Serv. Res. Pap. NC-245, 249, 1983.

237 Bridge, J.A. Fuelwood production of mixed hardwoods on mesic sites in Rhode Island. M.S. Thesis. University of Rhode Island, Kingston, Rhode Island, 72 pp, 1979.

238 Crow, T.R. and Erdmann, G.G. Weight and volume equa- tions and tables for red maple in the Lake States U.S. For. Serv. Res. Pap. NC-242, 14, 1983.

239 Bickelhaupt, D.H., Leaf, A.L. and Richards, N.A. Effect of branching habit on aboveground dry weight estimates of Acer saccharum stands. In: H.E. Young (Editor), IUFRO Biomass Studies: Nancy, France, and Vancouver, B.C., Canada. Uni- versity of Maine, College of Life Sciences and Agriculture, Orono, Maine, pp. 219-230, 1973.

240 Whittaker, R.H. and Woodwell, G.M. Dimension and production relations of trees and shrubs in the Brookhaven Forest, New York. J. Ecol., 56, 1-25, 1968.

241 Crow, T.R. Estimation of biomass in even-aged stand-re- gression and “mean tree” techniques. In: Forest Biomass Studies, 17th IUFRO Congress. Gainsville, Florida, l-20 March 1971, pp. 35-50, 1971.

242 Hegyi, F. Dry matter distribution in jack pine stands in northern Ontario. For. Chron., 48, 193-197, 1972.

243 Grigal, D.F. and Kemik, L.K. Generality of black spruce biomass estimation equations. Can. J. For. Res., 14, 468-470, 1984a.

244 Moore, T.R. and Verspoor, E. Aboveground biomass of black spruce stands in subarctic Quebec. Can. I. For. Res., 3, 596-598, 1973.

245 Ouellet, D. Biomass equations for black spruce in Quebec. Can. For. Serv. Inf. Rep. LAU-X-60(E), 27, 1983.

246 Harding, R.B. and Grigal, D.F. Individual tree biomass estimations for plantation-grown white spruce in northern Minnesota. Can. J. For. Res., 15, 738-739, 1985.

247 Hochbichler, E. Vorläufige Ergebnisse von Biomasseninventuren in Buchen-und Mittelwaldbeständen. In: Dietrich, H.-P ., Raspe, S. & Preuhsler, T. (eds.): Inventur von Biomasse- und Nährstoffvorräten in Waldbeständen. Forstliche Forschungsberichte, München, 186, 37–46, 2002.

248 Johansson, T. Biomass equations for determining functions of common and grey alder growing on abandoned farmland and some practical implicatons. Biomass and Bioenergy, 18, 147–159, 2000.

249 Johansson, T. Dry matter amounts and increment in 1- to 91-year-old common alder and grey alder and some practical implications. canadian Journal of Forest Research, 9, 1679–1690, 1999d.

250 Johansson, T. Biomass equations for determining functions of pendula and pubescent birches growing on abandoned farmland and some practical implicatons. Biomass and Bioenergy, 16, 3–38, 1999a.

251 Menguzzato, G. Modelli di previsione del peso fresco, della biomassa e del volume per pino insigne ed eucalitti nell’Azienda Massanova (Salerno). Ann. Ist. Sper. Selvicoltura, 19, 323–354, 1988.

252 Zianis, D. and Mencuccini, M. Aboveground biomass relationship for beech (Fagus moesiaca cz.) trees in Vermio Mountain, Northern Greece, and generalised equations for Fagus sp. Annals of Forest Science, 60, 439–448, 2003.

253 Cienciala, E., Cerný, M., Alptauer, J. and Exnerová, Z. Biomass functions applicable to European beech. Journal of Forest Science, 51(4), 147–154, 2005.

254 Pretzsch, H. Die Regeln von Reineke, Yoda und das Gesetz der räumlichen Allometrie. Allgemeine Forst- und Jagd-Zeitung, 171, 205–210, 2000.

255 Baldini, S., Berti, S., Cutini, A., Mannucci, M., Mercurio, R. and Spinelli, R. Prove sperimentali di primo diradamento in un soprassuolo di pino marittimo (Pinus pinaster Ait.) originato da incendio: aspetti silvicolturali, di utilizzazione e caratteristiche della biomassa. Ann. Ist. Sper. Selvicoltura, 20, 385–436, 1989.

256 Chroust, L. Above-ground biomass of young pine forests (Pinus sylvestris) and its determination. Communicationes Instituti Forestalis cechosloveniae, 14, 127–145, 1985.

257 Mäkelä, A. and Vanninen, P. Impacts of size and competition on tree form and distribution of aboveground biomass in Scots pine. canadian Journal of Forest Research, 28: 216–227, 1998.

258 Oleksyn, J., Reich, P.B., chalupka, W. and Tjoelker, M.G. Differential above- and below-ground biomass accumulation of European Pinus sylvestris populations in a 1 -year-old provenance experiment. Scandinavian Journal of Forest Research, 14, 7–17, 1999.

259 Rock, J. Suitability of published equations for aspen in central Europe – results from a case study. Submitted manuscript, 2005.

260 Menguzzato, G. and Tabacchi, G. Prove di diradamento su Pseudotsuga menziesii in calabria. Ambiente, tavole di cubatura e della biomassa epigea. Ann. Ist. Sper. Selvicoltura, 17, 255–293, 1986.

261 Hees, A.F.M.v. Biomass development in unmanaged forests. Nederlands Bosbouwtijdschrift, 73(5), 2–5, 2001

262 Susmel, l., Viola, F. and Bassalo, G. Ecologie della lecceta del Supramonte di Orgosolo. (Sardegna centro-orientale). Analisi de centro di Economia Montana delle Venezie, 10, 1–216, 1976.

263 Leonardi, S. and Rapp, M. Phytomasse et mineralomase d’un taillis de chene Vert du Massif de l’Etna. Ecologia Mediterranea, 8, 125–138, 1982.

264 Zapata-Cuartas, M. Ecuaciones de Biomasa Aerea para los Bosques Primarios del Area de Influence de la Central Hidroelectric Porce II. B.Sc. Thesis, UniversidadNacional de Colombia, Sede Medellin, 2001.

265 Bratti, M. R., Wrann, J. H. and Vita, A. A. Efecto de la altura de corte en el rebrote de Acacia saligna (Labill.) H. Wendl. Ciencia e investigacion Forestall - Instituto Forestal, Chile, 12, 40-50, 1988.

266 Brown, S. Estimating biomass and biomass change of tropical forests: a primer, Rome, FAO, 1997.

267 Scatena, F. N., Silver, W., Siccama, T., Johnson, A. and Sanchez, M. J. Biomass and Nutrient Content of the Bisley Experimental Watersheds, Luquillo Experimental Forest, Puerto Rico, Before and After Hurricane Hugo, 1989. Biotropica, 25, 15-27, 1993.

268 Chave, J., Riera, B. and Dubois, M. A. Estimation of biomass in a neotropical forest of French Guiana: spatial and temporal variability. Journal of Tropical Ecology, 17, 79-96, 2001.

269 Overmann, J. P., Witte, H. G. and Saldarriaga, J. G. Evaluation of regression models for aboveground biomass determination in Amazon rainforest. J. Trop. Ecol., 10, 1994.

270 Alvarez, E. Composición florística, diversidad, estructura y biomasa de un bosque inundable en la Amazonia Colombiana. M.Sc. Thesis, Universidad de Antioquia, 1993.

271 Crow, T. R. and Blank, R. W. Distribution of Biomass and Production for Several Northern Woody Species. Research Note NC-239, North central Forest Experiment Station, 1-3, 1978.

272 Rai, S. N. and Proctor, J. Ecological studies on four rainforests in Kartnataka, India I. Environment, structure, floristics, and biomass. Journal of Ecology, 439-454, 1986.

273 Higuchi, N., Santos, J., Ribeiro, R. J., Miente, L. and Biot, Y. Biomassa da parte aerea de vegetacao de floresta tropical umida da Terra Firme de Amazonia Brasileira. Acta Amazonica, 28, 153-166, 1998.

274 Edwards, J. P. and Grub, J. P. Studies of mineral cycling in a montane rainforest in New Guinea, I. The distribution of organic matter in the vegetation and soil. Journal of Ecology, 65, 943-969, 1977.

275 Case, B. and Hall, R.J. Assessing prediction errors of generalized tree biomass and volume equations for the boreal forest region of west-central Canada. Can. J. For. Res., 38, 878-889, 2008.

276 Navar, J. Allometric equations for tree species and carbon stocks for forests of northwestern Mexico. Forest Ecology and Management, 257, 427-434, 2009.

277 Colorado, G. J. Ecuaciones de Biomasa Aerea de los Arboles de los Bosques Secundarios del Area de Influencia de la Central Hidroelectrica. Thesis: Departamento de Ciencias Forestales, Universidad Nacional de Colombia, Sede Medellin, 2001.

278 Acosta-Mireles, M., Vargas-Herbabdez, J., Velazquez-Martinez, A. and Etchevers-Barra, J. D. Estimacion de la Biomasa Aerea Mediante el Uso de Relaciones Alometricas en Seis Especies Arboreas en Oaxaca, Mexico. Agrociencia 36, 725-736, 2002.

279 Guerra, J. C., Ganoso, J. A., Schlatter, J. V. and Nespolo, R. R. Analisis e la biomasa de las raices en diferentes tipos de bosques. Avances de la evaluacion de Pinus radiata en Chile., Bosque (Valdivia), 2005.

280 Schlegel, B., Gayoso, J. and Guerra, J. Manual de Procedimentos para Inventarios de Carbono en Ecosistemas Forestales. Proyecto FONDEF D98I1076, Universidad Austral de Chile, 2001.

281 Rodriguez-Laguna, R., Jimenez-Perez, J., Aguirre-Calderon, O. and Jurado-Ibarra, E. Ecuaciones alometricas paraestimar biomasa aerea en especies de encino y pino en Irurbide, N.L. . Ciencia Forestal en Mexico, 32, 39-56, 2007.

282 Nogueira, E. M. , Fearnside, P. M. , Nelson, B. W. , Barbosa, R. I. and Keizer, E. W. Estimates of forest biomass in the Brazilian Amazon: New allometric equations and adjustments to biomass from wood-volume inventories. Forest Ecology and Management, 256, 1853-1867, 2008.

283 Montero, M. and Montagnini, F. Modelos alometricos para la estimacion de biomasa de diez especies nativas en plantaciones enlaregion de Atlantica de Costa Rica. Revista Forestal Centroamericana, 2004.

284 Salis, S. M., Assis, M. A., Mattos, P. P. and Piao, A. S. S. Estimating the aboveground biomass and wood volume of savanna woodlands in Brazils Patanal wetlands based on allometric correlations. Forest Ecology and Management, 228, 61-68, 2006.

285 Montero, M. and Kanninen, M. Indice de Sitio Para Terminanalia amazonia en Costa Rica. Agronomia Costarricense, 27, 29-35, 2003.

286 Nascimento, H. E. M. and Laurance, W. F. Total aboveground biomass in central Amazonian rainforests: a landscape-scale study. Forest Ecology and Management, 5793, 1-11, 2001.

287 Nelson, R. F., Kimes, D. S., Salas, W. A. and Routhier, M. Secondary Forest Age and Tropical Forest Biomass Estimation Using Thematic Mapper Imagery. BioScience, 50, 419-431, 2000.

288 Brown, S. Measuring carbon in forests: current status and future challenges. Environmental Pollution, 116, 363-372, 2002.

289 Burner, D. M., Pote, D. H. and Ares, A. Foliar and shoot allometry of pollarded black locust, Robinia pseudoacacia L. Agroforest Syst, 68, 37-42, 2006.

290 Diaz-Franco, R., Acosta-Mireles, M., Carrillo-Anzures, F., Buendia-Rodrigues, E., Flores-Ayala, E. and Etchevers-Barra, J. D. Determinacion de ecuaciones alometricas para estimar biomasa y carbono en Pinus patula Schl. et Cham. Madera y Bosques, 13, 25-34, 2007.

291 Lescure, J. P., Puig, H., Riera, B., Leclerc, D., Beekman, A. and Beneteau, A. La phytomasse epigee dune foret dense en Guyane francaise. Acta Oecologia 4, 237-251, 1983.

292 Arajuo, T. M., Higuchi, N. and Carvalho, J. A. Comparison of formulae for biomass content determination in a tropical rain forest site in the state of Para, Brazil. Forest Ecology and Management, 117, 43-52, 1999.

293 Ovington, J. D. and Olson, J. S. Biomass and checmical content of El Verde lower montane rain forest plants. In: ODUM, H. T. & PIGEON, R. F. (eds.) A tropical rain forest: a study of irradiation and ecology at El verde, Puerto Rico. Springfield, Virginia: U.S. A.E.C.National Technical Information Service, 1970.

294 Hase, H. and Folster, H. Bioelement inventory of tropical (semi) evergreen seasonal forest on eutrophic alluvial soils, western Llanos, Venezuela. Acta Oecologica-Oecologia Plantarum, 3, 331-346, 1982.

295 Bond-Lamberty, B., Wang, B. C. and Gower, S. T. Aboveground and belowground biomass and sapwood area allometric equations for six boreal tree species of northern Manitoba[J] . Can. J. For. Res. , 32: 1441- 1450, 2002.

296 Basuki, T. M., Vanlaake, P. E., Skidmore, A. K. and Hussin, Y. A. Allometric equations for estimating the above-ground biomass in tropical Dipterocarp forests. Forest Ecology and Management, 257, 1684-1694, 2009.

297 Barclay, H. J., Pang, P. C. and Pollard, D. F. W. Aboveground biomass distribution within trees and stands in thinned and fertilized Douglas-fir. Canadian Journal of Forest Research, 16, 438-442, 1986.

298 Makela, A. and Vanninen, P. Impacts of size and competition on tree form and distribution of aboveground biomass in Scots pine. Canadian Journal of Forest Research, 28, 216-228, 1998.

299 Oleksyn, J., Reich, P. B., Chalupka, W. and Tjoelker, M. G. Differential Above- and Below-ground Biomass Accumulation of European Pinus sylvestris Populations in a 12-year-old Provenance Experiment. Scand J For Res, 14, 7-17, 1999.

300 Baldini, S., Berti, S., Cutini, A., Mannucci, M., Mercurio, R. and Spinelli, R. Prove sperimentali di primo diradamento in un soprassuolo di pino marittimo (Pinus pinaster Ait.) originato da incendio: aspetti silvicolturali, di utilizzazione e caratteristiche della biomassa. . Ann 1st Sper. Selvicoltura., 20, 385-436, 1989.

301 Chuankuan, W. Biomass allometric equations for 10 co-occurring tree species in Chinese temperate forests. Forest Ecology and Management 222 ,9–16, 2006.

302 Dimittris, Z. and Maurizio, M. On simplifying allometric analyses of forest biomass Forest Ecology and Management 187 ,311-332, 2004.

303 Asomaning, G. Carbon estimation of teak (Tectona grandis) and socio-economic impact of reforestation on some Ghanaian rural communities. Kumasi, Ghana, 2006.

304 Manlay, R.J., Kairé, M., Masse, D., Chotte, J.-L., Ciornei, G. and Floret, C. Carbon, nitrogen and phosphorus allocation in agro-ecosystems of a West African savanna I. The plant component under semi-permanent cultivation. Agriculture, Ecosystems & Environment, 88, 215–232, 2002.

305 Kairé, M. La production ligneuse des jachères et son utilisation par l’homme au Sénégal, Université de Provence Marseille, France, 1999.

306 Neya, B., Kaboré, C., Kiboa, D. and Sedego, T. Production de bois, élaboration d’un tarif de cubage dans la forêt du Nazinon Ouest (Sobaka). Rapport annuel du projet “Recherche sur l’amélioration et la gestion de la jachère en Afrique de l’Ouest – Projet 7 ACP RPR 269. CORAF, UE (DG VIII), . Burkina Faso. p. 55–78, 1998.

307 Peltier, R., Njiti, C.F., Ntoupka, M., Manlay, R., Henry, M. and Morillon, V .Evaluation du stock de carbone et de la productivité en bois d’un parc à Karités du Nord-Cameroun. Bois et forêt des tropiques, 294, 39–50, 2007.

308 Hailu, Z. Ecological impact evaluation of Eucalyptus plantations in comparison with agricultural and grazing land-use types in the Highlands of Ethiopia. Vienna University of Agricultural Sciences, Vienna, 2002.

309 Zewdie, M. Temporal changes of biomass production, soil properties and ground flora in Eucalyptus globulus plantations in the central highlands of Ethiopia. Faculty of Natural Resources and Agricultural Sciences, Uppsala, Sweden, 2009.

310 Avendaño, D., Acosta, M., Carrillo, F. and Etchevers, J. Estimación de la biomasa y carbono en un bosque de Abies religiosa. Rev Fitotec Mex, 32, 233–238, 2009.

311 Rodríguez, V. Estimación dasométrica de carbono almacenado en un bosque de Abies religiosa (H.B.K.) Schl. Et Cham. del paraje El Cedral del Parque Nacional El Chico Hidalgo. Thesis, Universidad Nacional Autónoma de México, 2013.

312 Návar, J. Allometric equations and expansion factors for tropical dry forest trees of eastern Sinaloa, Mexico. Trop Subtrop Agroecosyt, 10, 45–52, 2009b.

313 Acosta, M., Carrillo, F. and Gómez, R. Estimación de biomasa y carbono en dos especies de bosque mesófilo de montaña. Rev Mex de Cienc Agric, 2, 529–543, 2011.

314 Juárez, B. Uso de ecuaciones alométricas para estimar de biomasa y carbono de Alnus jorullensis H.B.K. spp. jurullensis en bosques mezclados de Tequexquinahuac, Texcoco, México. Thesis, Universidad Autónoma Chapingo, 2008.

315 Rodríguez, R., Jiménez, J., Meza, J., Aguirre, O. and Razo, R. Carbono contenido en un bosque tropical subcaducifolio en la reserva de la biosfera el cielo, Tamaulipas, México. Rev Latinoam Rec Nat, 4, 215–222, 2008.

316 Rodríguez, R., Jiménez, J., Aguirre, O. and Treviño, E. Estimación de carbono almacenado en un bosque de niebla en Tamaulipas, México. Ciencia-UANL,9, 179–187, 2006.

317 Rodríguez, R., Jiménez, J., Aguirre, O., Treviño, E. and Razo, R. Estimación de carbono almacenado en el bosque de pino-encino en la Reserva de la Biosfera el Cielo, Tamaulipas, México. Ra Ximhai, 5, 317–327, 2009.

318 Vigil, N. Estimación de biomasa y contenido de carbono en Cupressus lindleyi Klotzsch ex Endl. en el campo forestal experimental Las Cruces, Texcoco, México. Thesis, Universidad Autónoma Chapingo, 2010.

319 Monroy, C. and Návar, J. Ecuaciones de aditividad para estimar componentes de biomasa de Hevea brasiliensis Muell. Arg., en V eracruz, México. Madera Bosques, 10, 29–43, 2004.

320 Foroughbakhch, R., Alvarado, M., Hernández, J., Rocha, A., Guzmán, M. and Treviño, E. Establishment, growth and biomass production of 10 tree woody species introduced for reforestation and ecological restoration in northeastern Mexico. For Ecol Manag, 235, 194–201, 2006.

321 Méndez, J., Luckie, L., Capó, M. and Nájera, J. Ecuaciones alométricas y estimación de incrementos en biomasa aérea y carbono en una plantación mixta de Pinus devoniana Lindl. y P . pseudostrobus Lindl., en Guanajuato, México. Agrosciencia, 45, 479–491, 2011.

322 Jiménez, C. Uso de ecuaciones alométricas para estimar biomasa y carbono en la parte aérea de Pinus hartwegii Lindl., en el Parque Nacional Izta-Popo. Thesis, Universidad Autónoma Chapingo, 2010.

323 González, M. Estimación de la biomasa aérea y la captura de carbono en regeneración natural de Pinus maximinoi H. E. Moore, Pinus oocarpa var. ochoterenai Mtz. Y Quercus sp. en el norte del Estado de Chiapas, México. Thesis, Centro Agronómico Tropical de investigación y Enseñanza, 2008.

324 Bonilla, E. Uso de ecuaciones alométricas para estimar biomasa y carbono en Pinus montezumae Lamb. Thesis, Universidad Autónoma Chapingo, 2009.

325 Pacheco, G. Ecuaciones alométricas para estimar biomasa aérea por compartimentos en reforestaciones de Pinus patula Schl. et Cham., en Xiacuí, Ixtlan, Oaxaca. Thesis, Universidad de la Sierra Juárez, 2011.

326 Díaz, R. Determinación de ecuaciones alométricas para estimar biomasa y carbono en el estrato aéreo en bosques de Pinus patula Schl. Cham. en Tlaxcala México. Thesis, Universidad Nacional Autónoma Chapingo, 2005.

327 Díaz, R., Acosta, M., Carrillo, F., Buendía, E., Flores, E. and Etchevers, J. Determinación de ecuaciones alométricas para estimar biomasa y carbono en Pinus patula Schl. Cham Madera Bosques, 13, 25–34, 2007.

328 Aguirre, O. and Jiménez, J. Evaluación del contenido de carbono en bosques del sur de Nuevo León. Rev Mex Cien For, 2, 73–83, 2011.

329 Domínguez, G. Evaluación del contenido de carbono en bosques del sur de Nuevo León. Thesis, Universidad Autónoma de Nuevo León, 2005.

330 Rodríguez, R., Jímenez, J., Aguirre, O. and Jurado, E. Ecuaciones alométricas para estimar biomasa aérea en especies de encino y pino en Iturbide, N.L. Rev Cien For Mex, 32, 39–56, 2007.

331 Meraz, J., Rojas-García, F., Galarza, J., Torres, J., Luna, J., Ponce, A. and Romo, J. Utilización de ecuaciones alométricas para la estimación de biomasa y carbono en huertos de guayabo. IV Congreso Mexicano de Ecología. Villahermosa, México, 18–22, 2013.

332 Tomas, C. Estimación de biomasa y carbono medinte ecuaciones alometricas en Quercus laurina Humb. y Quercus rugosa Née en predios bajo manejo del ejido San Pablo Ixayoc, Estado de México. Thesis, Universidad Autónoma Chapingo, 2013.

333 Gómez, J., Etchevers, J., Monterrosos, A., Campo, J. and Tinoco, J. Ecuaciones alométricas para estimar biomasa y carbono en Quercus magnoliaefolia. Rev Chapingo Ser Cienc For Am, 17, 261–272, 2011.

334 Manzano, F. Crecimiento, periodicidad y biomasa de Zanthoxylum kellermaniiP . Wilson en una selva perennifolia del norte de Oaxaca. Thesis, Colegio de Postgraduados, 2010.

335 Martin, J.G., Kloppel, B.D., Schaefer, T.L., Kimbler, D.L. and McNulty, S.G.,Aboveground biomass and nitrogen allocation of ten deciduous southern Appalachian tree species. Can. J. For. Res. 28, 1648–1659, 1998.

336 Canadell, J., Riba, M. and Andres, P . Biomass equations for Quercus ilex L. in the Montseny Massif, northeastern Spain. Forestry, 61, 137–147, 1988.

337 Wang, J.R., Letchford, T., Comeau, P . and Kimmins, J.P . Aboveand below-ground biomass and nutrient distribution of a paper birch and subalpine fir mixed-species stand in the sub-boreal spruce zone of British Columbia. For. Ecol. Manage. 130, 17–26, 2000.

338 Barney, R.J., V ancleve, K. and Schlentner, R. Biomass distribution and crown characteristics in two Alaskan Picea mariana ecosystems. Can. J. For. Res. 8, 36–41, 1978.

339 Zavitkovsi, J., Jeffers, R.M., Nienstaedt, H. and Strong, T.F. Biomass production of several jack pine provenances at three Lake States locations. Can. J. For. Res. 11, 441–447, 1981.

340 Son, Y ., Hwang, J.W., Kim, Z.S., Lee, W.K. and Kim, J.S. Allometry and biomass of Korean pine (Pinus koraiensis) i n central Korea. Biores. Techn. 78, 251–255, 2001.

341 Forrest, W.G. Variations in the accumulation, distribution and movement of mineral nutrients in radiata pine plantations. Ph.D. Thesis, Canberra, 1969.

342 Ovington, J.D. Dry-matter production by Pinus sylvestris L. Ann. Bot. N. S. 21, 287–314, 1957.

343 Santa Regina, I., Tarazona, T. and Calvo, R. Aboveground biomass in a beech forest and a Scots pine plantation in the Sierra de la Demanda area of northern Spain. Ann. Sci. For. 54, 261–269, 1997.

344 VanLear, D.H., Waide, J.B. and Teuke, M.J. Biomass and nutrient content of a 41-year-old loblolly pine (Pinus taeda L.) plantation on a poor site in South Carolina. For. Sci. 30, 395–404, 1984.

345 Bartelink, H.H. Allometric relationships on biomass and needle area of Douglas-fir. For. Ecol. Manage. 86, 193–203, 1996.

346 Clough, B.F. and Scott, K. Allometric relationships for estimating above-ground biomass in six mangroves species. For. Ecol. Manage. 27, 117–127, 1989.

347 Eamus, D., McGuinness, K. and Burrows, W. Review of allometric relationships for estimating woody biomass for Queensland, the Northern Territory and Western Australia. National Carbon Accounting System Technical Report 5b. Australian Greenhouse Office, Canberra, 2000.

348 Ward, S.C. and Pikersgill, G.E. Biomass and nutrient distribution in eucalypt plantations growing on rehabilitated bauxite mines. Aust. J. Ecol., 10, 111–124, 1985.

349 Grove, T.S. and Malajczuk, N. Biomass production by trees and understorey shrubs in an age-series of Eucalyptus diversicolor F. Muell. Stand. For. Ecol. Manage. 11, 59–74, 1985.

350 Bennett, L.T., Weston, C.J. and Atwill, P .M. Biomass, nutrient content and growth responses to fertilisers of six-year-old Eucalyptus globulus plantations at three contrasting sites in Gippsland, Victoria. Aust. J. Bot. 45, 102–121, 1997.

351 Keith, H., Barrett, D. and Keenan, R. Review of allometric relationships for estimating woody biomass for New South Wales, the Australian Capital Territory, Victoria, Tasmania, and South Australia. National Carbon Accounting System Technical Report 5b. Australian Greenhouse Office, Canberra, 2000.

352 Snowdon, P ., Eamus, D., Gibbons, P ., Khanna, P .K., Keith, H., Raison, R.J. and Kirschbaum, M.U.F. Synthesis of allometrics, review of root biomass and design of future woody biomass sampling strategies. National Carbon Accounting System Technical Report 17. Australian Greenhouse Office, Canberra, 2000.

353 Applegate, G.B. Biomass of Blackbutt (Eucalyptus pilularis Sm.) forests on Fraser Island. Master’s Thesis, Armidale, 1982.

354 Kumar, B.M., George, S.J., Jamaludheen, K. and Suresh, T.K. Comparison of biomass production, tree allometry and nutrient use efficiency of multipurpose trees grown in woodlot and silvipastoral experiments in Kerala. India For. Ecol. Manage. 112, 145–163, 1998.

355 Morrison, I.K. Organic matter and mineral distribution in an old-growth Acer saccharum forest near the northern limit of its range. Can. J. For. Res. 20, 1332–1342, 1990.

356 Hughes, M.K. Tree biocontent, net primary production and litter fall in a deciduous woodland. Oikos, 22, 62–73, 1971.

357 Westman, W.E. and Rogers, R.W. Biomass and structure of a subtropical Eucalypt forest, North Stradbroke Island. Aust. J. Bot. 25, 171–191,1977.

358 Hingston, F.J., Dimmock, G.M. and Turton, A.G. Nutrient distribution in a Jarrah (Eucalyptus marginata Donn Ex Sm.) ecosystem in south-west Western Australia. For. Ecol. Manage. 3, 183–207, 1981.

359 Grierson, P .F., Williams, K. and Adams, M.A. Review of unpublished biomass-related information: Western Australia, South Australia, New South Wales and Queensland. National Carbon Accounting System Technical Report No. 25. Australian Greenhouse Office, Canberra, 2000.

360 Glossop, B.L. Biomass of the northern Jarrah forest and nutrient dynamics following a hot autumn fire. Hons. Thesis. Botany Department, University of Western Australia, Nedlands, WA, 1978.

361 Grove, T.S. and Malajczuk, N. Biomass production by trees and understorey shrubs in an age-series of Eucalyptus diversicolor F. Muell. Stand. For. Ecol. Manage. 11, 59–74, 1985.

362 O’Brien, N.D. Nutritional physiology of Eucalyptus grandis and Pinus radiata irrigated with municipal effluent. Ph.D. Thesis. University of Melbourne, 1998.

363 Barrett, D.J. Ecophysiological bases for the distribution of rainforest and eucalypt forest in south-east Australia. Ph.D. Thesis, Canberra, 992.

364 Todd, M.C. The role of nutrient cycling in the sustainability of young plant communities on mined sites. Ph.D. Thesis. University of Western Australia, Perth, WA, 2000.

365 Ward, S.C. and Koch, J.M. Biomass and nutrient distribution in a 15.5 year old forest growing on a rehabilitated bauxite mine. Aust. J. Ecol. 21, 309–315, 1996.

366 Bartelink, H.H. Allometric relationships for biomass and leaf area of beech (Fagus sylvatica L.). Ann. Sci. For. 54, 39–50, 1997.

367 Santa Regina, I., Tarazona, T. and Calvo, R. Aboveground biomass in a beech forest and a Scots pine plantation in the Sierra de la Demanda area of northern Spain. Ann. Sci. For. 54, 261–269, 1997.

368 Grove, T.S. Growth responses of trees and understorey to applied nitrogen and phosphorus in Karri forest. For. Ecol. Manage. 23, 87–103, 1988.

369 Lieffers, V .J. and Campbell, J.S. Biomass and growth of Populus tremuloides in northeastern Alberta: estimates using hierarchy in tree size. Can. J. For. Res. 14, 610–614, 1984.

370 Leonardi, S. and Rapp, M. Phytomasse et mineralomase d’un taillis de Chene Vert du Massif de l’Etna. Ecol. Medit. 8, 125–138, 1982.

371 Ferres, L., Roda, F., V erdu, A.M.C. and Terradas, J. Estructura y funcionalismo de un encinar montano en el Montseny. II. Biomasa Aera. Medit. 4, 23–36 (cited in Canadell), 1980.

**Text 2.** Lists of references used to build the global database of allometric model (Variable: DBH and H) parameters.

**Previous published syntheses referred to build the global database**

1. Ai, X.R., and Shen, Z.K.: Growth and biomass of Larix kaempferi plantation, Journal of Hubei Institute for Nationalities (Natural Sciences), 19(2), 20-22, 2001 (in Chinese).
2. Ai, X.R., and Zhou, G.L.: The biomass of Chinese fir plantation in the north boundary of middle subtropical zone, Hubei Forestry Science and Technology, (2), 17-20, 1996 (in Chinese).
3. Ai, X.R., Shen, Z.K., and Yi, Y.M.: Effect of stand density on the biomass of Pinus massoniana plantation, Hubei Forestry Science and Technology, (3), 16-18, 1998 (in Chinese).
4. Ai, X.R., Yao, L., Yi, Y.M., and Shen, Z.K.: Carbon storage of Cryptomeria fortunei plantation in Enshi Autonomous Prefecture, Journal of Hubei Institute for Nationalities (Natural Sciences), 19(2), 20-22, 2011 (in Chinese).
5. An, H.P., Jin, X.L., and Yang, C.H.: Growth rhythm and biomass dynamics of major vegetation types in Banqiaohe Watershed, Guizhou Forestry Science and Technology, 19(4), 20-34, 1991 (in Chinese).
6. Bai, Y.Q., and Zhan, H.Z.: The biomass of Larix gmelinii plantation, Forest Investigation Design, (1), 21-25, 1980 (in Chinese).
7. Bao, C.S.: Nutrient cycling in a birch forest, in: Long-term Located Research on Forest Ecosystems, Volume 1, Zhou, X.F., Wang, Y.H., Zhao, H.X., eds., Northeast Forestry University Press, Harbin, China, 217-227, 1991 (in Chinese).
8. Bao, C.S., Bai, Y., Qing, M., Chen, G.W., Zhang, Q.L., and Wang, L.M.: Productivity and carbon storage of Larix gmelinii natural forest, Journal of Inner Mongolia Agricultural University, 31(2), 77-82, 2010 (in Chinese).
9. Bao, X.C., Chen, L.Z., Chen, Q.L., Ren, J.K., Hu, Y.H., and Li, Y.: The biomass of planted oriental oak (Quercus variabilis) forest, Acta Phytoecologica et Geobotanica Sinica, 8(4), 313-320, 1984 (in Chinese).
10. Chen, C.G.: Biomass equations of Korean pine plantation, Forest Investigation Design, (2), 19-23, 1981 (in Chinese).
11. Chen, C.G., and Zhu, J.F.: Manual on Biomass Equations of Major Tree Species in Northeast China, China Forestry Publishing House, Beijing, China, 1989 (in Chinese).
12. Chen, C.G.: Biomass and productivity of tree layers in Pinus armandii forests, Qinling Mountains, Journal of Northwestern College of Forestry, (1), 1-18, 1984 (in Chinese).
13. Chen, H.J.: Biomass and nutrient distribution in a Chinese-fir plantation chronosequence in Southwest Hunan, China, Forest Ecology and Management, 105, 209-216, 1998.
14. Chen, W.R.: Study on the dynamics of aboveground net productivity of Alniphyllum fortunei plantation, Journal of Fujian Forestry Science and Technology, 27(3), 31-34, 74, 2000 (in Chinese).
15. Chen, X.G.: The biomass and allometric equation of a 20-years-old Cunninghamia lanceolata plantation, Protection Forest Science and Technology, (4), 28-29, 40, 2007 (in Chinese).
16. Chen, C.G., and Guo, X.F.: The biomass of broadleaved Korean pine forest, Forest Investigation Design, (2), 10-19, 6, 1984 (in Chinese).
17. Chen, C.G., and Peng, H.: Standing crops and productivity of the major forest types at Huoditang Forest Region of Qinling Mountains, Journal of Northwest Forestry College, 11(S), 92-102, 1996 (in Chinese).
18. Chen, B.H., Li, H.Q., and Liu, J.G.: Biomass of Populus diversifolia natural forest in middle reach of Tarim River, Xinjiang, Xinjiang Forestry Science and Technology, (3), 8-16, 1984a (in Chinese).
19. Wang, S.J., Chen, B.H., and Li, H.Q.: Populus euphratica Forests, China Environmental Science Press, Beijing, China, 1995.
20. Chen, C.G., Gong, L.Q., Peng, H., and Liu, X.Z.: Biomass and productivity of Quercus aliena var. acuteserrata forests in Qinling Mountains, Journal of Northwest Forestry College, 11(S), 103-114, 1996 (in Chinese).
21. Chen, D.X., Li, Y.D., Luo, T.S., Lin, M.X., and Sun, Y.X.: Biomass and net primary productivity of Podocarpus imbricatus plantation in Jianfengling, Hainan Island, Forest Research, 17(5), 598-604, 2004 (in Chinese).
22. Chen, L.N., Xiao, Y., Gai, Q., and Ji, W.X.: Preliminary study on the biomass of Larix principis-rupprechtii forest in Pangquangou Nature Reserve: Community structure, biomass, and net primary productivity, Journal of Shanxi Agricultural University, 11(3), 240-247, 1991 (in Chinese).
23. Chen, L.Z., Ren, J.K., Bao, X.C., Chen, Q.L., Hu, Y.H., Miao, Y.G., and Li, Y.: Community characteristics and biomass of Chinese pine plantation in Xishan Region, Beijing, Acta Phytoecologica et Geobotanica Sinica, 8(3), 173-181, 1984b (in Chinese).
24. Chen, T., Wen, Y.G., Sun, Y.P., and Liang, H.W.: Preliminary study on the biomass and productivity of Eucalyptus urophylla × E. grandis plantations with successive rotations, Guangxi Forestry Science, 34(1), 8-12, 2005 (in Chinese).
25. Chen, Z.X., He, Y.J., Bai, F.M., Zhang, J.H., and Li, Z.H.: Effects of stand density on the biomass and productivity of Pinus massoniana air-seeding stands, Journal of Central South Forestry University, 21(1), 44-47, 2001 (in Chinese).
26. Cheng, Y.X., and Li, Z.X.: Preliminary study on the biomass of three Larix gmelinii forests, Inner Mongolia Forestry Investigation and Design, (4), 29-39, 1989 (in Chinese).
27. Cheng, T.R., Ma, Q.Y., Feng, Z.K., and Luo, X.: Study on forest biomass in Xiaolong Mountains, Gansu Province, Journal of Beijing Forestry University, 29(1), 31-36, 2007 (in Chinese).
28. Cheng, X.Q., Han, H.R., and Kang, F.F.: Biomass, carbon accumulation and their partitioning of a Pinus tabuliformis plantation ecosystem in Shanxi Province, China, Chinese Journal of Ecology, 31(10), 2455-2460, 2012 (in Chinese).
29. Cheng, Y., Hong, W., Wu, C.Z., and Qi, X.H.: Aboveground biomass and its productivity of Schima superba population, Chinese Journal of Applied and Environmental Biology, 15(3), 318-322, 2009 (in Chinese).
30. Dai, H.J., He, H.J., Zhao, X.H., Zhang, C.Y., Wang, J.S., and Yang, S.: Biomass allocation patterns and allometric models of two dominant tree species in broadleaved and Korean pine mixed forest, Chinese Journal of Applied and Environmental Biology, 19(4), 718-722, 2013 (in Chinese).
31. Dang, C.L., and Wu, Z.L.: Study on the biomass of Pinus yunnanensis forest. Acta Botanica Yunnanica, 13(1), 59-64, 1991 (in Chinese).
32. Ding, B.Y., and Sun, J.H.: Study on biological productivity and nutrient cycling of artificial Korean pine forest ecosystem, Journal of Northeast Forestry University, 17(S), 1-98, 1989 (in Chinese).
33. Ding, G.J., Wang, P.C., and Yan, R.F.: Study on the biomass dynamics and modeling of Masson pine pulpwood stands, Scientia Silvae Sinicae, 34(1), 33-41, 1998 (in Chinese).
34. Dong, L.H., Li, F.R., and Jia, W.W.: Development of tree biomass model for Pinus koraiensis plantation, Journal of Beijing Forestry University, 34(6), 16-22, 2012 (in Chinese).
35. Du, W.Z.: The individual biomass of Pinus tabuliformis plantation in Xiaolong Mountains, Gansu Province, Gansu Science and Technology, 28(23), 153-154, 70, 2012 (in Chinese).
36. Fan, J.J.: Biomass model of Larix olgensis plantation in Dongzhelenghe Nature Reserve, Shanxi Forestry Science and Technology, 41(4), 26-28, 2012 (in Chinese).
37. Fang, W., and Wang, G.Q.: Biomass and productivity of farmland shelterbelt network, Forest Science and Technology, (6), 11-13, 1989.
38. Fan, S.H., Liu, G.L., Zhang, Q., Feng, H.X., Zong, Y.C., and Ren, H.Q.: Biomass and productivity of Populus xiaohei plantation on sandy land in north China, Forest Research, 23(1), 71-76, 2010 (in Chinese).
39. Fan, Z.F., An, Y.T., and Zhao, X.H.: Biomass and productivity of Pinus tabuliformis plantation, Journal of Beijing Forestry University, 19(S2), 93-98, 1997 (in Chinese).
40. Fang, X., and Tian, D.L.: Dynamics of carbon stock and sequestration in Chinese fir plantation, Guihaia, 26(5), 516-522, 2006 (in Chinese).
41. Fang, C.L., Zhu, X.W., and Zhang, H.C.: Preliminary study on the biomass and productivity of Picea crassifolia natural secondary forests, Journal of Qinghai University, 9(1), 71-77, 1991 (in Chinese).
42. Fang, H.B., Tian, D.L., and Kang, W.X.: Biomass dynamics of a thinned Chinese fir plantation ecosystem, Journal of Central South Forestry University, 19(1), 16-19, 1999 (in Chinese).
43. Feng, L., and Yang, Y.G.: Biomass and productivity of three Larix gmelinii virgin forests, Scientia Silvae Sinicae, 21(1), 86-92, 1985 (in Chinese).
44. Feng, Z.L., Tang, J.W., Zheng, Z., Song, Q.S., Cao, M., Zhang, J.H., and Xie, J.W.: Biomass dynamics of the pioneer Trema orientalis community in the early stages of secondary succession of tropical forest in Xishuangbanna, Chinese Journal of Ecology, 18(5), 1-6, 1999 (in Chinese).
45. Feng, Z.L., Zheng, Z., Tang, J.W., Song, Q.S., and Zhang, J.H.: Biomass of tropical secondary Mallotus paniculatus forest in Xishuangbanna, Chinese Journal of Ecology, 24(3), 238-242, 2005 (in Chinese).
46. Feng, Z.W., Zhang, J.W., Chen, C.Y., Wang, K.P., Zhao, J.L., Zeng, S.Y., and Ma, J.X.: Biological productivity and nutrient distribution in artificial Michelia macclurei stand, Journal of Northeastern Forestry Institute, 11(2), 13-20, 1983 (in Chinese).
47. Fu, Z.J.: Study on synecological features and biomass of Larix chinensis forest in Taibai Mountain, Journal of Hanzhong Teachers College (Natural Sciences), (2), 69-72, 1994 (in Chinese).
48. Gao, C.J., Tang, G.Y., Sun, Y.Y., Zhang, C.H., Xie, Q.H., and Li, K.: Biomass and allocation of young Azadirachta indica and Acacia auriculiformis for different restoration patterns in dry-hot valley, Journal of Zhejiang A&F University, 29(4), 482-490, 2012 (in Chinese).
49. Gao, H.Y., Zhou, G.Y., Zhou, Z.P., Zhao, H.B., and Qiu, Z.J., Aboveground biomass of 27-year-old Cunninghamia lanceolata plantation in Tianjingshan Forest Farm, Guangdong Province, Guangdong Forestry Science and Technology, 29(4), 1-6, 2013 (in Chinese).
50. Gao, H.Z., You, L.Q., and Wang, C.: Individual biomass and productivity of Pinus tabuliformis plantation in Yanshan Mountain, Journal of Hebei Forestry Science and Technology, (4), 7-9, 2009 (in Chinese).
51. Gao, S.C., Tian, D.L., Yan, W.D., Zhu, F., Fang, X., and Liang, X.C., Pattern characteristics of stand biomass of urban forest in Changsha City, Journal of Central South University of Forestry and Technology, 30(12), 56-65, 2010 (in Chinese).
52. Gu, Y.K., Chen, B.G., and Feng, Y.H.: An investigation on the aboveground biomass and its productivity of artificial Cunninghamia lanceolata stands in Xijiang Region, Guangdong Province, Journal of South China Agricultural University, 8(1), 41-50, 1987 (in Chinese).
53. Guan, D.S.: The biomass and productivity of four stands in the forest area of Liuxihe Reservoir, Ecological Science, (2), 45-52, 1986 (in Chinese).
54. Han, M.Z.: The biomass and production of Larix gmelinii plantation, Acta Agriculturae Boreali-Sinica, 2(4), 134-138, 1987 (in Chinese).
55. Han, M.Z.: The biomass and net primary productivity of a Dahurian larch-birch forest ecosystem, in: Long-term Research on China's Forest Ecosystems, Department of Science and Technology of Ministry of Forestry, eds., Northeast Forestry University Press, Harbin, China, 451-458, 1994 (in Chinese).
56. Han, Y.Z., and Liang, S.F.: Study on the tree root system and its biomass of Larix principis-rupprechtii plantation, Shanxi Forestry Science and Technology, (3), 36-40, 1997 (in Chinese).
57. Han, Y.Z., Li, Y.E., Liang, S.F., and Li, H.Y.: Study on the tree biomass of Larix principis-rupprechtii plantation, Journal of Shanxi Agricultural University, 17(3), 278-283, 1997 (in Chinese).
58. Han, F.Y., Zhou, Q.Y., Chen, S.X., Chen, W.P., Li, T.H., Wu, Z.H., and Jian, M.: The biomass and energy of two different aged Eucalyptus stands, Forest Research, 23(5), 690-696, 2010 (in Chinese).
59. He, F., Wang, Y.Q., Tan, X.F., and Wang, C.N.: The biomass and nutrient cycle in Vernicia fordii plantations, Non-wood Forest Research, 8(2), 6-20, 1990 (in Chinese).
60. He, H.Z., Huang, L.H., Duan, X., and He, R.K.: Study on the biomass in main afforestation tree species of the second-ring forest belt of Guiyang City, Guizhou Science, 25(3), 33-39, 2007 (in Chinese).
61. He, H.Z., Song, J.X., Liu, Y.Y., Zhang, Y.W., and Huang, L.H.: The biomass and allocation of Chinese fir forest in southeast Guizhou, Guangdong Agricultural Sciences, (21), 58-60, 2013 (in Chinese).
62. He, Y.J., Qin. L., Li. Z.Y., Shao. M.X., Liang. X.Y., and Tan, L.: Carbon storage capacity of a Betula alnoides stand and a mixed Betula alnoides × Castanopsis hystrix stand in southern subtropical China: A comparison study, Acta Ecologica Sinica, 32(23), 7586-7594, 2012 (in Chinese).
63. Hong, T., Wu, C.Z., Lin, Y.M., Chen, C., Li, J., and Lin, H.: Biomass characteristics in the arbor layer of Vernicia montana plantation, Journal of Mountain Science, 30(6), 648-654, 2012 (in Chinese).
64. Hong, Y.C., Xu, W.Q., Ye, G.F., and Zhang, L.H.: Model for estimating biomass of Casuarina equisetifolia planation in coastal region of the southeastern China, Journal of Zhejiang Forestry Science and Technology, 30(4), 66-69, 2010 (in Chinese).
65. Wu, S.D., Ye, G.F., Pan, H.Z., Xu, J.S., Long, X.W., Zheng, R., and Huang, C.Y.: Biomass and its distribution of Casuarina equisetifolia plantation, Protection Forest Science and Technology, (S1), 21-24, 1996 (in Chinese).
66. Hu, Y.H., and Pang, Q.L.: The biomass and distribution pattern of Cunninghamia lanceolata plantation in western Hubei, Hubei Forestry Science and Technology, (3), 6-9, 2012 (in Chinese).
67. Hu, D.L., Li, Z.H., and Xie, X.D.: The biomass and productivity of Pinus taiwanensis plantation, Journal of Central South Forestry University, 18(1), 60-64, 1998 (in Chinese).
68. Hu, J.R., Huang, R.K., Zeng, H.D., Li, P.Y., and Wang, Z.X.: Individual biomass model of mature Pinus massoniana plantation, Forestry Prospect and Design, (1), 9-12, 2011 (in Chinese).
69. Hu, S.S., Zhang, Y.T., Li, J.M., Lu, J.J., Li, X., Wang, Q.J., and Wang, X.K.: Biomass distribution of Populus alba var. pyramidalis plantation, Xinjiang Agricultural Sciences, 49(6), 1059-1065, 2012 (in Chinese).
70. Huang, D.C.: The biomass of Cryptomeria fortunei plantation, Journal of Southwest Forestry College, (1), 23-28, 1986 (in Chinese).
71. Huang, Z.Z., and Bi, J.: Study on the biomass of Robinia pseudoacacia stands in Taihang Mountains, Journal of Hebei Forestry Science and Technology, (2), 48-52, 1992 (in Chinese).
72. Huang, L.M., Xue, L., Wang, X.E., Xie, T.F., Ren, X.R., and Cao, H.: Growth and biomass allocation of young Acacia auriculiformis stands under different densities, Journal of South China Agricultural University, 29(3), 52-55, 2008 (in Chinese).
73. Huang, T., Zhong, Q.P., and Peng, X.Y.: Study on the biomass and productivity of Liriodendron chinense plantation, Jiangxi Forestry Science and Technology, (5), 4-9, 2000 (in Chinese).
74. Huang, X.S., Wu, C.Z., Hong, W., Li, Z.K., and Cheng, Z.P.: The relationship between stand density and biomass of two rotation Chinese fir plantations, Journal of Fujian College of Forestry, 31(2), 102-105, 2011 (in Chinese).
75. Hui, G.Y., Luo, Y.W., and Zhang, X.L.: The productivity of Chinese fir (Cunninghamia lanceolata) plantation at hilly area in Dagang Mountain, Jiangxi Province, Scientia Silvae Sinicae, 25(6), 564-569, 1989 (in Chinese).
76. Ji, Y.H., Zhang, J.L., and Kang, L.X.: A study on biomass equations for Metasequoia glyptostroboides shelterbelt in the coastal agroforestry, Journal of Jiangsu Forestry Science and Technology, 24(2), 1-5, 1997 (in Chinese).
77. Jia, Y., and Qi, L.X.: The relationship between Lophodermium maximum and the biomass of Pinus koraiensis plantation, Journal of Northeast Forestry University, 16(5), 7-14, 1988 (in Chinese).
78. Jia, Y., and Zhang, F.: The biomass of Pinus koraiensis plantation in Caohekou Forest Region, Liaoning Province, Journal of Liaoning Forestry Science and Technology, (5), 18-23, 1985 (in Chinese).
79. Jia, K.X., Zheng, Z., and Zhang, Y.P.: Changes in the aboveground biomass of rubber plantations along an elevation gradient in Xishuangbanna, Chinese Journal of Ecology, 25(9), 1028-1032, 2006 (in Chinese).
80. Jiang, H.: A study on the biomass and productivity of Picea purpurea natural forest, Acta Phytoecologica et Geobotanica Sinica, 10(2), 146-152, 1986 (in Chinese).
81. Jiang, H.: Study on the biomass of Quercus liaotungensis and Betula dahurica forest in Dongling Mountain, in: Studies on Structures and Functions of Warm-temperate Forest Ecosystems, Chen, L.Z., Huang, J.H., eds., Science Press, Beijing, China, 104-115, 1997 (in Chinese).
82. Jiang, Z.L., and Zhao, S.: Study on the biomass of Loblolly pine plantation, in: Proceedings of forest ecosystems on Xiashu Ecological Station, Jiang, Z.L., eds., China Forestry Publishing House, Beijing, China, 10-15, 1992 (in Chinese).
83. Jiang, H., and Zhu, J.J.: Study on the biomass and productivity of Picea asperata natural forest, Journal of Sichuan Forestry Science and Technology, 7(2), 5-13, 1986 (in Chinese).
84. Jiang, T., Zhao, M., Zhang, S.Z., Yuan, M.L., and Huang, X.R.: Individual biomass and its allocation pattern of Pinus tabuliformis in Hebei, Hebei Journal of Forestry and Orchard Research, 27(3), 239-244, 2012 (in Chinese).
85. Jie, J.L., Zhan, Y.S., Huang, W.C., Long, W., Luo, Y.C., Hu, H.Y., and Xie, Z.R.: Study on the biomass of Pinus elliottii stand near Jinggangshan Line of Jing-Jiu Railway, Jiangxi Forestry Science and Technology, (2), 17-20, 2002 (in Chinese).
86. Jin, A.L.: Study on the biomass of main tree species in Bayingzhuang Forest Farm, Hebei Province, Thesis for Master’s Degree, Beijing Forestry University, Beijing, China, 53pp., 2012 (in Chinese).
87. Jin, A.L., Rao, L.Y., Li, J., and Zhang, T.: The biomass of Larix principis-rupprechtii plantation, Guangdong Agricultural Sciences, (12), 165-168, 2012 (in Chinese).
88. Kong, F.B., and Fang, H.: Comparative study on the biomass of Pinus taeda plantations with different densities and ages, Forestry Science and Technology, 28(3), 6-9, 2003 (in Chinese).
89. Li, B.T.: Preliminary study on biomass investigation method of Chinese fir plantation, Forest Resources Management, (6), 57-60, 1988 (in Chinese).
90. Li, H.L.: Carbon storage and carbon budget of poplar-crop intercropping ecosystem in the agricultural region of northern Jiangsu Plain, PhD Dissertation, Nanjing Forestry University, Nanjing, China, 125pp., 2010a (in Chinese).
91. Li, H.L., Chen, L.B., Fang, S.Z., and Sun, Q.X.: Comparison of carbon storage and distribution in different poplar-crop intercropping patterns, Scientia Silvae Sinicae, 45(11), 9-14, 2009b (in Chinese).
92. Li, Z.: Study on the biomass of Platyclatdus orientalis plantation in Xuzhou City, Thesis for Master’s Degree, Nanjing Forestry University, Nanjing, China, 36pp., 2010b (in Chinese).
93. Li, J.Z., Qin, W.M., Qin, Y., Qin, J., Tang, K., and Duan, W.W.: The biomass and productivity of Manglietia glauca plantation, Journal of Fujian Forestry Science and Technology, 38(1), 1-5, 2011c (in Chinese).
94. Li, Z.H.: Effect of thinning on Cunninghamia lanceolata plantation in the eastern Hunan Province, Scientia Silvae Sinicae, 36(S1), 131-136, 2000 (in Chinese).
95. Li, S.L., and Hou, J.Z.: The biomass of Populus tomentosa clonal plantation, Acta Agriculturae Universitatis Henanensis, 29(2), 134-140, 1995 (in Chinese).
96. Li, H., Li, C.Y., Zha, T.S., Liu, J.L., Jia, X., Wang, X.P., Chen, W.J., and He, G.M.: Patterns of biomass allocation in an age-sequence of secondary Pinus bungeana forests in China, The Forestry Chronicle, 90(2), 169-176, 2014.
97. Li, D.L., Jiang, P., and Wang, Y.F.: Individual biomass and productivity of Larix principis-rupprechtii plantation in Yanshan Mountain, Hebei Journal of Forestry and Orchard Research, 26(4), 334-339, 2011a (in Chinese).
98. Li, G.X., Meng, G.T., Fang, X.J., Lang, N.J., Yuan, C.M., and Wen, S.L.: Community characteristics and the biomass of Alnus cremastogyne plantation in central Yunnan Plateau, Journal of Zhejiang Forestry College, 23(4), 362-366, 2006a (in Chinese).
99. Li, J.H., Li, C.J., and Peng, S.K.: Study on the biomass expansion factor of poplar plantation, Journal of Nanjing Forestry University (Natural Sciences Edition), 31(4), 37-40, 2007a (in Chinese).
100. Li, J.L., Liang, S.C., and Cheng, S.Z.: A preliminary study on the community characteristics of Keteleeria davidiana var. chien-peii forest in Guizhou, Guizhou Science, 11(4), 7-11, 1993 (in Chinese).
101. Li, J.L., Liang, S.C., and Cheng, S.Z.: Preliminary study on the biomass of Keteleeria davidiana var. chien-peii population in Qingyan Town, Guizhou Province, Journal of Guizhou Normal University (Natural Sciences), 15(1), 7-12, 1997 (in Chinese).
102. Li, S.H., Shi, J.N., and Lei, P.: The biomass and its vertical distribution of the second-generation Chinese fir plantation, Journal of Sichuan Forestry Science and Technology, 28(1), 64-67, 2007b (in Chinese).
103. Li, W.B., Bao, W.K., He, B.H., Wu, W.Y., and Li, F.L.: Biomass distribution and its influencing factors of Pinus tabuliformis plantations in the Dagou Valley of the upper Minjiang River, Journal of Mountain Science, 25(2), 236-244, 2007c (in Chinese).
104. Li, X.C., Huang, L.B., Gui, G.R., Li, Z.J., Fan, K.S., Lu, H.L., Du, X.X., Ma, W.M., Yang, J.M., and Li, Y.L.: Effects of initially planted density on young Chinese fir plantation for construction timber, Journal of Jiangsu Forestry Science and Technology, 23(3), 1-6, 1996a (in Chinese).
105. Li, X.G., Xu, J.Y., and Zhai, C.Y.: Study on the biomass of Gordonia acumenata community in Jinyun Mountains, in: Ecological Research on Evergreen Broadleaved Forests, Zhong, Z.C., eds., Southwest Normal University Press, Chongqing, China, 234-250, 1988 (in Chinese).
106. Li, X.R., Liu, Q.J., Chen, Y.R., Hu, L.L., and Yang, F.T.: Aboveground biomass of three coniferous plantations in Qianyanzhou Research Station, Chinese Journal of Applied Ecology, 17(8), 1382-1388, 2006b (in Chinese).
107. Li, Y., Zhang, J.G., Duan, A.G., and Xiang, C.W.: Selection of biomass estimation models for Chinese fir plantation, Chinese Journal of Applied Ecology, 21(12), 3036-3046, 2010a (in Chinese).
108. Li, Z., Wang, Z.Y., Wang, Y., Guan, Q.W., Wei, W., Dong, P., and Zhang, H.N.: Effect of thinning on the biomass of Platyclatdus orientalis plantation, China Forestry Science and Technology, 24(1), 68-71, 2010b (in Chinese).
109. Li, Z.H., He, L.X., Zhou, Y.P., and Zhou, Q.K.: The biomass and productivity of Metasequoia glyphostrobodes plantation, Journal of Central South Forestry University, 16(2), 47-51, 1996b (in Chinese).
110. Liang, H.W., Wen, Y.G., Wu, G.X., Huang, X.Z., Zhou, G.F., and Chen, D.L.: Effects of successive rotations on growth and productivity of Eucalyptus urophylla × E. grandis short-rotation plantation, Journal of Fujian Forestry Science and Technology, 35(3): 14-18, 2008 (in Chinese).
111. Liang, K.N., Zhou, W.L., and Li, Y.Q.: Effects of fertilization on biomass and nutrient contents of young Eucalyptus urophylla cl. MLA plantation, Forest Research, 17(3), 327-333, 2004 (in Chinese).
112. Liang, N., Wang, W.B., and Tian, K.: Biomass distribution characteristics of 4- and 13-year-old Betula alnoides plantations, Journal of West China Forestry Science, 35(4), 188-192, 2006 (in Chinese).
113. Liao, H.Z., Zheng, Y.M., Zhang, C.N., and Chen, D.Y.: Study on the biomass of Cinnamomum camphora plantation, Forest Science and Technology, (9), 15-18, 1986 (in Chinese).
114. Lin, W.F., and Lin, D.X.: Biomass and productivity of Pinus massoniana plantation, Journal of Heilongjiang Vocational Institute of Ecological Engineering, 23(5), 19-21, 2010 (in Chinese).
115. Lin, S.M., Xu, T.G., and Zhou, G.M.: Study on the biomass of Chinese fir plantation, Journal of Zhejiang Forestry College, 8(3), 288-294, 1991 (in Chinese).
116. Lin, W.H., Chen, K.M., and Liu, Z.G.: Biomass and nutrient element content of Eucalyptus camaldulensis plantations of dry-hot valley in southwest Sichuan, Mountain Research, 12(4), 251-255, 1994 (in Chinese).
117. Ling, L.: Growth rules of individual biomass of Populus wenxianica forest, Protection Forest Science and Technology, (3), 9-11, 2011 (in Chinese).
118. Liu, B.: Study on individual biomass and allocation pattern of Pinus tabuliformis natural forest in Helan Mountains, China, Thesis for Master’s Degree, Northwest Agricultural and Forestry University, Yangling, China, 46pp., 2010 (in Chinese).
119. Liu, Q.: Biomass and productivity of different age-group Pinus massoniana plantations, Journal of Central South Forestry University, 16(4), 47-51, 1996 (in Chinese).
120. Liu, T.T.: Calculating biomass and carbon storage of poplar plantation based on tree structure, Thesis for Master’s Degree, Beijing Forestry University, Beijing, China, 52pp., 2009.
121. Liu, W.Y.: Study on the biomass and productivity of Acacia dealbata plantation in the protected district of water sources in north Kunming, Guihaia, 15(4), 327-334, 1995b (in Chinese).
122. Liu, K., and Chen, Y.E.: Biological productivity of Robinia pseudoacacia plantation in the Loess Plateau Area of the north Weihe River, Acta Botanica Boreali-Occidentalia Sinica, 9(3), 197-201, 1989 (in Chinese).
123. Liu, X.Z., and Cai, B.Y.: Comparative study between the biomass of Pinus massoniana plantation and P. elliottii plantation, Forest Resources Management, (5), 28-31, 1993 (in Chinese).
124. Liu, X.Z., and Kang, W.X.: The biomass of Eucommia ulmoides plantation, Phellodendron chinense plantation, and Magnolia officinalis var. biloba plantation, in: Long-term Located Research on Forest Ecosystems, Liu, X.Z., Kang, W.X., Chen, X.Y., Wen, S.Z., eds., China Forestry Publishing House, Beijing, China, 49-52, 1993 (in Chinese).
125. Liu, Z.G., and Ma, Q.Y.: An approach to methods for estimating biomass of Larix principis-rupprechtii artificial forests, Journal of Beijing Forestry University, 14(S), 105-113, 1992 (in Chinese).
126. Liu, G.Y., Zhao, G.H., Wang, G.H., Ma, C.M., and Li, J.: Biomass allocation pattern of Larix principis-rupprechtii plantation, Hebei Journal of Forestry and Orchard Research, 26(3), 222-226, 2011a (in Chinese).
127. Liu, S.R., Chai, Y.X., Cai, T.J., and Peng, C.H.: Study on the biomass and net primary productivity of Dahurian larch plantation, Journal of Northeast Forestry University, 18(2), 40-45, 1990 (in Chinese).
128. Liu, X.Z., Xiang, W.H., and Kang, W.X.: Biomass dynamical pattern in a Chinese fir plantation ecosystem, in: Long-term Located Research on Forest Ecosystems, Liu, X.Z., Kang, W.X., Chen, X.Y., Wen, S.Z., eds., China Forestry Publishing House, Beijing, China, 8-22, 1993 (in Chinese).
129. Liu, X.Z., Wen, S.Z., and Xiang, W.H.: Effect of thinning on the biomass of Pinus massoniana plantation, Forest Resources Management, (5), 43-48, 1995a (in Chinese).
130. Liu, X.Z., Tian, D.L., Kang, W.X., and Fang, H.B.: The biomass of a young second-generation Chinese fir plantation, Scientia Silvae Sinicae, 33(2), 61-66, 1997 (in Chinese).
131. Liu, Y.B., Zhang, Y.L., Zhao, T.S., Li, Z.L., Shi, Z.X., Li, Z.A., Zhang, Y.L., Meng, X.T., Hao, Z.Y., Mao, X.Q., Zhang, Y.S., and Dong, C.Y.: Biomass and productivity of the agroforestry ecosystems, Journal of Henan Agricultural College, (1), 13-20, 1984 (in Chinese).
132. Liu, Y.C., Jiang, Y.B., Chen, H.W., and Li, J.: Regression equations for individual tree of Betula alnoides plantation, Journal of Fujian Forestry Science and Technology, 35(2), 42-46, 2008 (in Chinese).
133. Liu, Z.Q., Chen, G.H., Meng, Y.Q., Li, J.G., and Liu, M.R.: Biomass and nutrient storage of Larix principis-rupprechtii plantation, Forest Research, 8(1), 88-93, 1995b (in Chinese).
134. Lu, S.W., Cao, Y.S., Li, F.S., Chen, F.J., Liu, T., and Yang, Z.: The volume and biomass of Larix principis-rupprechtii plantation in mountainous areas of northwest Hebei Province, Forest Resources Management, (1), 33-36, 2012 (in Chinese).
135. Lu, Y.S., Liang, Z.H., Wu, Z.X., Cai, Y.P., Zhou, K.M., Yang, G.F., and Yin, Z.X.: Biomass and productivity of main afforestation tree species on the seawall of northern Jiangsu, Journal of Jiangsu Forestry Science and Technology, 27(2), 12-15, 2000 (in Chinese).
136. Luan, K.Z., and Liu, Z.G.: Biomass estimation models for Pinus sylvestris var. mongolica plantation, Forestry Science and Technology, 37(3), 35-38, 2012 (in Chinese).
137. Luo, R.: Effect of intermediate cutting on biological productivity of Pinus massoniana plantation, Journal of Sichuan Forestry Science and Technology, 13(2), 29-34, 1992 (in Chinese).
138. Luo, W.X., Liu, G.Q., Tang, D.R., and Ma, S.T.: Growth increment and biomass of Eucommia ulmoides plantation in Weibei Loess Plateau, Journal of Northwest Forestry University, 9(4), 22-26, 1985 (in Chinese).
139. Luo, Y.J., Zhang, X.Q., Wang, X.K., Zhu, J.H., Zhang, Z.J., Sun, G.S., and Gao, F.: Biomass and its distribution patterns of Larix principis-rupprechtii plantations in northern China, Journal of Beijing Forestry University, 31(1), 13-18, 2009 (in Chinese).
140. Ma, Q.Y.: A study on the biomass and primary productivity of Chinese pine (Pinus tabuliformis Carr.) forests, PhD dissertation, Beijing Forestry University, Beijing, China, 178pp., 1988 (in Chinese).
141. Ma, Q.Y.: A study on the biomass of Chinese pine forests, Journal of Beijing Forestry University, 11(4), 1-10, 1989 (in Chinese).
142. Ma, W.: Measurement and estimation of ecosystem carbon density for Larix olgensis plantation based on FIM and FFE-FVS, PhD Dissertation, Beijing Forestry University, Beijing, China, 206pp., 2012 (in Chinese).
143. Ma, W., Sun, Y.J., Guo, X.Y., Ju, W.Z., and Mu, J.S.: Carbon storage of Larix olgensis plantation at different stand ages, Acta Ecologica Sinica, 30(17), 4659-4667, 2010 (in Chinese).
144. Mei, L.: Fine root turnover and carbon allocation in Manchurian ash and Dahurian larch plantations, PhD Dissertation, Northeast Forestry University, Harbin, China, 102pp., 2006 (in Chinese).
145. Mei, L., Zhang, Z.W., Gu, J.C., Quan, X.K., Yang, L.J., and Huang, D.: Carbon and nitrogen storages and allocation in tree layers of Fraxinus mandshurica and Larix gmelinii plantations, Chinese Journal of Applied Ecology, 20(8), 1791-1796, 2009 (in Chinese).
146. Meng, L., Cheng, J.M., Yang, X.M., Han, J.J., Fan, W.J., and Hu, X.J.: Carbon storage and density of Pinus tabuliformis plantation in Ziwuling Forest Region of the Loess Plateau, Bulletin of Soil and Water Conservation, 30(2), 133-137, 2010 (in Chinese).
147. Ming, A.G., Tang, J.X., Yu, H.L., Shi, Z.M., Lu, L.H., Jia, H.Y., and Cai, D.X.: Individual biomass regression model of Mytilaria laosensis in southwest Guangxi, Forest Resources Management, (6), 83-87, 93, 2011 (in Chinese).
148. Ming, A.G., Zhang, Z.J., Chen, H.H., Zhang, X.Q., Tao, Y., and Su, Y.: Effect of thinning on the biomass and carbon storage in Pinus massoniana plantation, Scientia Silvae Sinicae, 49(10), 1-6, 2013 (in Chinese).
149. Mo, D.X.: Study on carbon and nitrogen pattern and ecosystem biomass of Cryptomeria fortunei plantation of different density in southeastern Guangxi Province, Thesis for Master’s Degree, Guangxi University, Nanjing, China, 52pp., 2013 (in Chinese).
150. Qin, S.J., Li, K., Mo, D.X., and Wu, Q.B.: Biomass regression model of Cryptomeria fortunei plantation in southeast Guangxi, Journal of Southern Agriculture, 44(2), 261-265, 2013 (in Chinese).
151. Mu, T.M.: Study on the biomass of Picea crassifolia forest in Helan Mountains, Inner Mongolia Forestry Science and Technology, (1), 34-45, 1981 (in Chinese).
152. Ning, X.B., and Liu, Q.: Biological productivity of young Pinus massoniana plantation in different regions, Forest Resources Management, (1), 48-51, 1996 (in Chinese).
153. Ouyang, S.L., Dai, C.D., Hou, Y.N., Xu, Y.X., and Luo, J.: Biomass models for main constructive tree species of the protection forest system around Dongting Lake, Hunan Forestry Science and Technology, 37(5), 22-24, 2010 (in Chinese).
154. Pan, K.W., and Liu, Z.G.: The biomass of 10-year-old Cercidiphyllum japonicum plantation, Chinese Journal of Applied and Environmental Biology, 5(2), 121-130, 1999 (in Chinese).
155. Pan, F.J., Zhang, Z.F., Huang, Y.Q., and Mo, L.: Aboveground biomass of Cyclobalanopsis glauca analyzed by tree-ring method in karst region, Guihaia, 32(4), 464-467, 2012 (in Chinese).
156. Pan, P., Li, R.W., Xiang, C.H., Zhu, Z.F., and Yin, X.M.: The biomass and productivity of Cupressus lusitanica plantation, Resources and Environment in the Yangtze Basin, 11(2), 133-136, 2002 (in Chinese).
157. Pan, W.C., Li, L.C., Gao, Z.H., Zhang, X.Q., and Tang, D.Y.: The biomass and productivity of Chinese fir plantations, Hunan Forestry Science and Technology, (5), 1-12, 1978 (in Chinese).
158. Pan, W.C., Tian, D.L., Li, L.C., and Gao, Z.H.: Study on the nutrient cycling in Chinese fir plantation: (1) Biomass allocation and nutrient dynamics of Chinese fir plantations with different stand ages, Journal of Central South Forestry Institute, (1), 1-21, 1981 (in Chinese).
159. Pan, W.C., Tian, D.L., Lei, Z.X., and Kang, W.X.: Study on the nutrient cycling in Chinese fir plantation: (2) Content, accumulation rate and biological cycling of nutrient elements in the fast-growing Chinese fir forest in the hilly regions, Journal of Central South Forestry Institute, 3(1), 1-17, 1983 (in Chinese).
160. Pan, Y.J., Wang, B., Chen, B.F., and Peng, Q.Z.: Carbon sink of Chinese fir plantation ecosystem in Dagang Mountain, Jiangxi Province, Journal of Central South University of Forestry and Technology, 33(10), 120-125, 2013 (in Chinese).
161. Pang, J.P.: Carbon storage and its allocation of rubber plantation in Xishuangbanna, southwest China, Thesis for Master’s Degree, Xishuangbanna Tropical Botanical Garden, Chinese Academy of Sciences, Xishuangbanna, China, 54pp., 2009 (in Chinese).
162. Tang, J.W., Pang, J.P., Chen, M.Y., Guo, X.M., and Zeng, R.: Biomass and its estimation model of rubber plantations in Xishuangbanna, southwest China, Chinese Journal of Ecology, 28(10), 1942-1948, 2009 (in Chinese).
163. Peng, S.L., and Zhang, Z.P.: Biomass and primary productivity of two dominant species, Cryptocarya concinna and C. chinensis, of forest vegetation in Dinghu Mountain, Acta Phytoecologica et Geobotanica Sinica, 14(1), 23-32, 1990 (in Chinese).
164. Peng, S.L., and Zhang, Z.P.: Biomass and primary productivity of dominant species Aporosa yunnanensis and Blastus cochinchinensis of forest vegetation in Dinghu Mountain, Chinese Journal of Applied Ecology, 3(3), 202-206, 1992 (in Chinese).
165. Peng, P.H., Peng, J.S., Wang, C.S., and Wang, J.X.: The biomass and productivity of Populus schneideri var. tibetica plantation, Forestry Science and Technology, 28(4), 14-18, 2003 (in Chinese).
166. Peng, S.L., Li, M.G., and Lu, Y.: A preliminary study on the biomass and productivity of Pinus massoniana population in Dinghushan Biosphere Reserve, in: Tropical and Subtropical Forest Ecosystem, Volume 5, Dinghushan Forest Ecosystem Station, eds., Science Press, Beijing, China, 75-82, 1989 (in Chinese).
167. Peng, S.L., Yu, Z.Y., Zhang, W.Q., and Zeng, X.P.: Coenological analysis of five man-made forests on the downland of Heshan, Guangdong Province, Acta Phytoecologica et Geobotanica Sinica, 18(1), 1-10, 1992 (in Chinese).
168. Peng, S.L., Fang, W., Cao, H.L., Yu, Z.Y., and Ren, H.: Influence of human disturbance on tropical artificial Eucalyptus forest ecosystem, Acta Ecologica Sinica, 15(S1), 31-37, 1995 (in Chinese).
169. Qi, L.H.: Study on the comprehensive managing technologies of aerially seeded Pinus massoniana stands in Hunan Province, Thesis for Master’s Degree, Central South Forestry University, Zhuzhou, China, 59pp., 2003 (in Chinese).
170. Qi, L.H., Zhang, X.D., Zhou, J.X., Li, Z.H., Huang, L.L., and Yang, M.H.: Changing regularity and structural characteristics of the biomass and productivity of aerially seeded Pinus massoniana plantation, Forest Research, 20(3), 344-349, 2007 (in Chinese).
171. Qian, G.Q.: Dynamics of the net primary productivity of Liquidambar formosana plantation, Acta Agriculturae Universitatis Jiangxiensis, 22(3), 399-404, 2000 (in Chinese).
172. Qin, Z.G.: Community features and the biomass of Alnus cremastogyne fuelwood forest, Journal of Sichuan Forestry Science and Technology, 13(1), 24-28, 33, 1992 (in Chinese).
173. Qin, J., Meng, H.S., Qin, W.M., Yu, J.M., and Qin, D.W.: The biomass and productivity of Tsoongiodenron odorum plantation, China Forestry Science and Technology, 25(6), 65-68, 2011a (in Chinese).
174. Qin, L., He, Y.J., Li, Z.Y., Shao, M.X., Liang, X.Y., and Tan, L.: Allocation pattern of biomass and productivity for three plantations of Castanopsis hystrix, Pinus massoniana and their mixture in south subtropical area of Guangxi, China, Scientia Silvae Sinicae, 47(12), 17-21, 2011b (in Chinese).
175. Shen, Y.: Biomass and nutrient cycling of natural secondary mixed forest in subtropical area, Thesis for Master’s Degree, Central South University of Forestry and Technology, Changsha, China, 116pp., 2011 (in Chinese).
176. Shen, Y., Tian, D.L., Yan, W.D., and Xiao, Y.: Biomass and its distribution of natural secondary Quercus fabri + Sassafras tsumu + Cunninghamia lanceolata community in Yuanling County, Hunan Province, Journal of Central South University of Forestry and Technology, 31(5), 44-51, 2011a (in Chinese).
177. Shen, Y.Z., Sun, X.M., Zhang, J.T., Du, Y.C., and Ma, J.W.: Individual tree biomass of Larix kaempferi plantation in Xiaolong Mountain, Gansu Province, Forest Research, 24(4), 517-522, 2011b (in Chinese).
178. Shen, Z.K., Lu, S.P., and Ai, X.R.: The biomass and productivity of Larix kaempferi plantation, Journal of Hubei Institute for Nationalities (Natural Science Edition), 23(3), 289-292, 2005 (in Chinese).
179. Shi, Y.L.: Study on the ecosystem biomass of the artificial Cunninghamia lanceolata forest in Changling (Wanli District), Nanchang City, Acta Agriculturae Universitatis Jiangxiensis, 11(4), 32-45, 1989 (in Chinese).
180. Shi, P.L., Zhong, Z.C., and Li, X.G.: A study on the biomass of alder-cypress artificial mixed forest in Sichuan, Acta Phytoecologica Sinica, 20(6), 524-533, 1996 (in Chinese).
181. Shi, P.L., Yang, X., and Zhong, Z.C.: Dynamics of population biomass and its density-dependent regulation in alder and cypress mixed forest, Chinese Journal of Applied Ecology, 8(4), 341-346, 1997 (in Chinese).
182. Shi, Z., Liu, J., Lan, X., Liang, D., Lu, M.B., and Deng, M.Y.: Individual biomass regression models of Pinus massoniana plantation in Tianlin County, Guangxi Province, Guangxi Forestry Science, 38(3), 167-170, 2009 (in Chinese).
183. Si, J.P., Yao, R.M., Chen, D.B., and Wu, C.H.: The biomass of Magnolia officinalis plantation, Journal of Zhejiang Forestry College, 10(2), 162-168, 1993 (in Chinese).
184. Su, Y.M.: Study on the biomass and productivity of Larix kaempferi plantation, Journal of Sichuan Forestry Science and Technology, 16(3), 36-42, 1995 (in Chinese).
185. Su, Y.M., Liu, X.L., Xiang, C.H.: Study on the biomass and net primary productivity of Abies fabri plantation, Journal of Sichuan Forestry Science and Technology, 21(2), 31-35, 2000 (in Chinese).
186. Sun, B.G., Chen, F., Wang, J.M., Chen, X.M., Yang, Z.X., Cai, X.Y., and Li, B.: Biomass distribution pattern of Pinus yunnanensis with different diameter classes, Forest Research, 25(1), 71-76, 2012a (in Chinese).
187. Sun, Q.X., Yu, F.A., and Peng, Z.H.: The biomass of poplar plantation in the beach land of Yangtze River, Forest Science and Technology, (3), 4-6, 1998 (in Chinese).
188. Wu, Z.M., Sun, Q.X., and Duan, W.X.: Relationship between flooded situation and poplar growth on beach land of Yangtze river in Anhui, Chinese Journal of Applied Ecology, 11(1), 25-29, 2000 (in Chinese).
189. Tang, W., and Xu, R.Q.: Study on the biomass of Abies fabri plantation, Sichuan Forestry Exploration and Design, (2), 27-32, 1993 (in Chinese).
190. Tang, J.W., Zhang, J.H., Song, Q.S., Cao, M., Feng, Z.L., Dang, C.L., and Wu, Z.L.: A preliminary study on the biomass of secondary tropical forest in Xishuangbanna, Acta Phytoecologica Sinica, 22(6), 489-498, 1998 (in Chinese).
191. Tang, L.Z., Yu, M.K., Yan, C.F., Liu, Z.L., and Fang, S.Z.: Effects of site condition and cultivation on the growth of sawtooth oak plantations, Journal of Fujian College of Forestry, 28(2), 130-135, 2008 (in Chinese).
192. Tang, W.P., Wang, Y.R., and Zheng, L.Y.: Study on the biomass and productivity of southern type poplar plantation, Hubei Forestry Science and Technology, (S), 43-47, 2004b (in Chinese).
193. Tian, D.L., Pan, W.C., Lei, Z.X., Long, E.H., and Cai, B.Y.: A preliminary study on the biomass and density effects of Pinus massoniana plantation, Journal of Central South Forestry Institute, 2(1), 41-50, 1982 (in Chinese).
194. Tian, D.L., Pan, H.H., Kang, W.X., and Fang, H.B.: The biomass of a second-generation Chinese fir plantation, Journal of Central South Forestry University, 18(3), 11-16, 1998 (in Chinese).
195. Tian, D.L., Xiang, W.H., Yan, W.D., and Kang, W.X.: Effect of successive rotation on the biomass and productivity of Chinese fir plantation at fast-growing stage, Scientia Silvae Sinicae, 38(4), 14-18, 2002 (in Chinese).
196. Tian, D.L., Yin, G.Q., Fang, X., Xiang, W.H., and Yan, W.D.: Carbon density, storage and spatial distribution under different ‘Grain for Green’ patterns in Huitong, Hunan Province, Acta Ecologica Sinica, 30(22), 6297-6308, 2010 (in Chinese).
197. Tian, Q.F., Du, L.H., and Li, X.J.: Study on the biomass of Robinia pseudoacacia plantation in Xishan National Forest Park, Beijing, Journal of Beijing Forestry University, 19(S2), 104-107, 1997a (in Chinese).
198. Tian, Q.F., Yan, H.P., and Wang, P.X.: Comparison of the productivity between three deciduous broadleaved plantations, Journal of Beijing Forestry University, 19(S2), 118-122, 1997b (in Chinese).
199. Tian, Q.F., Zhou, R.W., and Zhang, J.S.: Study on the biomass of Quercus variabilis plantation in Xishan National Forest Park, Beijing, Journal of Beijing Forestry University, 19(S2), 113-117, 1997c (in Chinese).
200. Yan, H.P., Li, H., and Zhang, J.S.: Study on the biomass of Acer truncatum forest, Journal of Beijing Forestry University, 19(S2), 108-112, 1997 (in Chinese).
201. Tong, J.Q.: Study on the productivity and growth of Chinese fir plantations with different sites and densities, Journal of Fujian Agriculture and Forestry University (Natural Science Edition), 37(4), 369-373, 2008 (in Chinese).
202. Wan, S.C.: The biomass allocation patterns of constructive tree species in forest-swamp ecotone, Journal of Mudanjiang University, 22(8), 124-127, 2013 (in Chinese).
203. Wan, M., Tian, D.L., Fan, W., and Li, Q.Y.: Biomass production and carbon sequestration in poplar-crop agroforestry ecosystems in eastern Henan Plain, Scientia Silvae Sinicae, 45(8), 27-33, 2009 (in Chinese).
204. Wang, M.B.: The biomass and afforestation prospect of Pinus tabuliformis plantation in the northwest Shanxi Province, Journal of Shanxi University (Natural Science Edition), (4), 98-102, 1988 (in Chinese).
205. Wang, M.B.: The biomass of Populus hopeiensis forest, Journal of Shanxi University (Natural Science Edition), 14(1), 103-107, 1991 (in Chinese).
206. Wang, W.C.: The aboveground biomass of Pinus tabuliformis plantation, Shanxi Forestry Science and Technology, (2), 10-15, 1985 (in Chinese).
207. Wang, X.Y.: Carbon storage distribution of Larix olgensis plantation at different stand ages, PhD Dissertation, Beijing Forestry University, Beijing, China, 96pp., 2011 (in Chinese).
208. Wang, X.Y., Sun, Y.J., and Ma, W.: Biomass and carbon storage distribution of Larix olgensis plantations with different density, Journal of Fujian College of Forestry, 31(3), 221-226, 2011b (in Chinese).
209. Wang, J., and Wen, Z.W.: Aboveground biomass distribution and predictive model of secondary Betula luminifera forest in the northwest Guizhou Province, Guizhou Forestry Science and Technology, 39(2), 18-21, 2011 (in Chinese).
210. Wang, Y.M., and Liu, B.Z.: Ecological Characteristics of Protection Forests in the Loess Plateau, China Forestry Publishing House, Beijing, China, 42-48, 1994 (in Chinese).
211. Wang, Z.F., and Feng, Z.K.: The parameter estimation of tree biomass using the nonlinear least square method, Journal of Jilin Agricultural University, 28(3), 261-264, 2006 (in Chinese).
212. Wang, B., Nie, D.P., Guo, Q.S., and Xia, L.F.: Studies on Forest Ecosystems in Dagang Mountains of Jiangxi Province, China Science and Technology Press, Beijing, China, 183-185, 2003a (in Chinese).
213. Wang, C., Gao, H.Z., and Zang, Y.Q.: Study on the individual biomass of Betula platyphylla natural secondary forest, Forestry Science and Technology, 35(1), 7-9, 13, 2010 (in Chinese).
214. Wang, D.Y., Li, D.Y., and Feng, X.Q.: Forest Ecosystems on Warm Temperate Zone, China Forestry Publishing House, Beijing, China, 190-205, 2003b (in Chinese).
215. Wang, H.M., Chen, Z.J., and Li, X.W.: Biomass and nutrient content of different forest-grass ecosystems converted from farmlands, Research of Soil and Water Conservation, 12(2), 125-128, 2005 (in Chinese).
216. Wang, H.Y., Wang, W.J., Qiu, L., Su, D.X., An, J., Zheng, G.Y., and Zu, Y.G.: Differences in biomass, litter layer mass and SOC storage changing with tree growth in Larix gmelinii plantations in northeast China, Acta Ecologica Sinica, 32(3), 833-843, 2012a (in Chinese).
217. Wang, J.S., Zhang, C.Y., Fan, X.H., and Zhao, Y.Z.: Biomass allocation patterns and allometric models of Abies nephrolepis, Acta Ecologica Sinica, 31(14), 3918-3927, 2011a (in Chinese).
218. Wang, J.S., Fan, X.H., Fan, J., Zhang, C.Y., and Xia, F.C.: Effect of aboveground competition on biomass partitioning of understory Korean pine (Pinus koraiensis), Acta Ecologica Sinica, 32(8), 2447-2457, 2012b (in Chinese).
219. Wang, J.Y., Che, K.J., Fu, H.E., Chang, X.X., Song, C.F., and He, H.Y.: Study on the biomass of water conservation forest on the north slope of Qilian Mountains, Journal of Fujian College of Forestry, 18(4), 319-323, 1998 (in Chinese).
220. Wang, Q.C., Wang, Q.L., Li, D., Wu, N.S., and Cheng, Z.C.: Suitable management density of multi-benefit management pattern in Pinus elliottii plantation on hilly region of Jiangxi Province, Chinese Journal of Applied Ecology, 12(5), 663-666, 2001 (in Chinese).
221. Wang, X.W., Li, X.Y., Guan, X.D., and Bai, G.X.: Preliminary study on the biomass of Larix olgensis plantation, Journal of Liaoning Forestry Science and Technology, (6), 30-34, 1993 (in Chinese).
222. Wang, X.Y., Hu, D., and He, J.S.: Study on the biomass of Fagus engleriana forest and Quercus aliena var. acuteserrata forest in Shennongjia Region, Journal of Capital Normal University (Natural Science Edition), 28(2), 62-67, 2007a (in Chinese).
223. Wang, Y., Li, Q., Zhang, P.Y., Wu, X.X., Jiang, Y.Z., and Jiang, J.T.: The biomass and nutritive element content of the fast-growing poplar plantations, Journal of Shandong Forestry Science and Technology, (2), 1-7, 1990 (in Chinese).
224. Wang, Y.T., Ma, Q.Y., Hou, G.W., Kan, Z.G., and Chen, Y.: Dynamics of the biomass and productivity of the naturally-regenerated Pinus densata forest in the burned areas of the western Sichuan Province, Forestry Science and Technology, 32(1), 37-40, 2007b (in Chinese).
225. Wen, D.C., Deng, Z.R., and Xiang, H.H.: Study on the productivity of Ussuri poplar plantation, Journal of Northeast Forestry University, 15(2), 42-49, 1987 (in Chinese).
226. Wen, S.Z., Pan, W.C., Tian, D.L., and Kang, W.X.: Hydroecological benefits from Chinese fir plantation ecosystem: Preliminary report of comprehensive trial of small forested watershed, Journal of Central South Forestry College, 9(S), 11-22, 1989 (in Chinese).
227. Wen, Y.G., Liang, H.W., and Jiang, H.P.: The biomass and its distribution of Chinese fir plantations in Guangxi, Journal of Guangxi Agricultural University, 14(1), 55-64, 1995 (in Chinese).
228. Wen, Y.G., He, T.P., Li, X.X., Liang, H.W., Zhou, J.Y., and Su, Y.J.: The biomass and productivity of Eucalyptus exserta coastal protection plantation in Hepu County, Guangxi, Journal of Guangxi Agricultural and Biological Science, 19(1), 1-5, 2000a (in Chinese).
229. Wen, Y.G., Liang, H.W., Zhao, L.J., Zhou, M.Y., He, B., Wang, L.H., Wei, S.H., Zheng, B., Liu, D.J., and Tang, Z.S.: Biomass and productivity of Eucalyptus urophylla plantation, Journal of Tropical and Subtropical Botany, 8(2), 123-127, 2000b (in Chinese).
230. Wu, Q.Z.: Study on the biomass of Mytilaria laosensis plantation, Journal of Fujian Forestry Science and Technology, 32(3), 125-129, 2005 (in Chinese).
231. Wu, H.X., Shi, Y.G., Zhang, H.Z., and Zhang, S.C.: Study on the biomass of Pinus tabuliformis plantation in Badaling Forest Farm, Hebei Journal of Forestry and Orchard Research, 21(3), 240-242, 2006 (in Chinese).
232. Wu, P., Ding, F.J., Cui, Y.C., Zhu, J., and Li, C.R.: The biomass and productivity of young Pinus massoniana forests of Pearl River Shelterbelt Construction Project in Qiannan City, Guizhou Agricultural Sciences, 40(6), 169-172, 2012a (in Chinese).
233. Wu, S.R., Yang, H.Q., Hong, R., Zhu, W., and Chen, X.Q.: The biomass and its distribution of Pinus massoniana plantation, Journal of Fujian Forestry Science and Technology, 26(1), 18-21, 1999 (in Chinese).
234. Wu, W.Y., Huang, Q.F., and Huang, C.L.: The biomass and growth increment of poplar plantations in the Nangeng marshlands of Yangtze River, Anhui Forestry Science and Technology, 38(2), 53-57, 72, 2012c (in Chinese).
235. Wu, Z.M., Sun, Q.X., and Chen, M.G.: Biomass and nutrient accumulation of poplar plantation in the beach land of Yangtze River in Anhui Province, Chinese Journal of Applied Ecology, 12(6), 806-810, 2001 (in Chinese).
236. Xiang, W.H., Chen, X.Y., and Cai, B.Y.: The time-lapse characteristics of the biomass in slash pine plantation, in: Long-term Located Research on Forest Ecosystem, Liu, X.Z., Kang, W.X., Chen, X.Y., Wen, S.Z., eds., China Forestry Publishing House, Beijing, China, 60-64, 1993 (in Chinese).
237. Xiao, Y.: Study on the productivity of Betula platyphylla natural secondary forest, Acta Biologica Plateau Sinica, (8), 147-157, 1988 (in Chinese).
238. Xiao, Y.: Comparative study on the biomass and productivity of Pinus tabuliformis plantations in different climatic zones, Shaanxi Province, Acta Phytoecologica et Geobotanica Sinica, 14(3), 237-246, 1990 (in Chinese).
239. Xiao, X.C., Li, Z.H., Tang, Z.J., Sheng, J.M., Gong, X.J., and Zhu, N.: Effects of stand density on the biomass and productivity of Pinus elliottii plantation, Journal of Central South University of Forestry and Technology, 31(3), 123-129, 2011 (in Chinese).
240. Xiao, Y., Wu, B.S., Chen, B.Q., Zhang, J.C., Wang, M.H., and Wang, L.G.: A preliminary study on the aboveground biomass of Pinus tabuliformis plantation, Shanxi Forestry Science and Technology, (2), 5-14, 1983 (in Chinese).
241. Xiao, Y.T., He, A.H., and Liang, J.Z.: Community characteristics and the biomass of Pinus taiwanensis natural forest in Mufu Mountain of Hubei-Hunan border, Hunan Forestry Science and Technology, (4), 1-4, 1989 (in Chinese).
242. Xie, W.D., Wen, Y.G., Zhou, M.Y., Liang, H.W., Liu, S.R., and Chen, F.: Biomass and productivity of Eucalyptus urophylla plantation in new cultivated areas, Journal of Central South University of Forestry and Technology, 27(5), 13-18, 2007 (in Chinese).
243. Xie, W.D., Wen, Y.G., Liang, H.W., Li, X.X., He, B., Zhong, J.B., Liao, G.S., and Liu, J.: The biomass and productivity of Casuarina equisetifolia shelterbelt in the coastal sandy land of Guangxi, Protection Forest Science and Technology, (5), 6-8, 2008 (in Chinese).
244. Xie, W.D., Ye, S.M., Yang, M., and Zhao, L.J.: Biomass and distribution pattern of Pinus massoniana plantation in the southeast Guangxi, Journal of Beihua University (Natural Science Edition), 10(1), 68-71, 2009 (in Chinese).
245. Xie, Z.S., Chen, B.G., Han, J.G., and Deng, Y.S.: The biomass estimative model of two Eucalypts in Leizhou Peninsula, in: Studies on the Eucalypt Ecosystem of Short Rotation in Leizhou, Zeng, T.X., eds., China Forestry Publishing House, Beijing, China, 66-75, 1995 (in Chinese).
246. Xu, J.H., Wang, M.L., Huang, Q.F., and Gong, S.F.: Aboveground biomass model of Quercus acutissima natural forest, Anhui Forestry Science and Technology, 37(4), 3-6, 2011a (in Chinese).
247. Xu, J.W., Li, C.R., Wang, W.D., Qiao, Y.J., Cheng, H.Y., and Wang, Y.H.: The biomass and productivity of Pinus thunbergii protective forests in sandy coastal area, Journal of Northeast Forestry University, 33(6), 29-32, 2005 (in Chinese).
248. Xu, X.N., and Li, H.K.: Growth characteristics of a mixed plantation of Chinese fir and Pinus taiwanensis, Forest Research, 9(3), 278-283, 1996 (in Chinese).
249. Xue, P.: Growth and biomass of 6-year-old Eucalyptus urophylla plantation in Leizhou Forestry Bureau, Eucalypt Science and Technology, 26(1), 18-21, 2009 (in Chinese).
250. Xue, X.K., and Sheng, W.T.: Study on the biomass of Fokienia hodginsii plantation in Zhuting Town, Hunan Province, Forest Science and Technology, (4), 16-19, 1993 (in Chinese).
251. Yan, R.F., and Zhu, S.Q.: Comparison of biomass estimates of Pinus massoniana plantation, Collection of Guizhou Agricultural College, (4), 101-105, 1984 (in Chinese).
252. Yang, L.: The biomass model of Populus tomentosa plantation in Daxing District, Beijing, Hebei Journal of Forestry and Orchard Research, 26(4), 345-348, 2011 (in Chinese).
253. Zhang, J.F.: Determination of the biomass of Chinese white poplar plantation in Daxing District, Beijing, Contemporary Eco-Agriculture, (3-4), 54-57, 2010a (in Chinese).
254. Yang, T.: The biomass and root distribution of Pinus massoniana and Quercus acutissima natural secondary mixed forest, Journal of Xinyang Agricultural College, 14(4), 4-6, 9, 2004 (in Chinese).
255. Yang, X.: The biomass and productivity of the intercropping ecosystem with paulownia (Paulownia elongata) and crops, Acta Agriculturae Universitatis Henanensis, 20(4), 485-509, 1986 (in Chinese).
256. Yang, D., and Yang, X.Q.: Study on the biomass and productivity of Pinus tabuliformis plantation in Wufeng Mountain, Wudu County, Gansu Province, Journal of Northwest Normal University (Natural Science Edition), 40(1), 70-75, 2004 (in Chinese).
257. Yang, Q.P., Li, M.G., Wang, B.S., Li, R.W., and Wang, C.W.: Dynamics of the biomass and net primary productivity in succession of south subtropical forests in Southwest Guangdong, Chinese Journal of Applied Ecology, 14(12), 2136-2140, 2003a (in Chinese).
258. Yang, Q.P., Wang, B., Guo, Q.R., Zhao, G.D., Fang, K., and Liu, Y.Q.: Effects of Phyllostachys edulis expansion on carbon storage of evergreen broadleaved forest in Dagang Mountain, Jiangxi, Acta Agriculturae Universitatis Jiangxiensis, 33(3), 529-536, 2011 (in Chinese).
259. Yang, R., Deng, Z.J., Qin, M.C., and Dai, P.Y.: Study on the biomass of Cupressus funebris plantation in the hilly region, the middle of Sichuan Province, Sichuan Forestry Science and Technology, 8(1), 21-24, 1987 (in Chinese).
260. Yang, Y.L., Gao, J.B., Cao, F., Lu, D.B., Zhao, Q.X., Wu, Y.X., and Lu, Z.M.: Effect of thinning on the growth of Larix gmelinii, Journal of Jilin Forestry Science and Technology, 32(5), 21-24, 2003b (in Chinese).
261. Yang, Z.W., Tan, F.L., Xiao, X.X., Chen, L.S., and Zhuo, K.F.: Study on the biomass of Fokienia hodginsii plantation, Scientia Silvae Sinicae, 36(S1), 120-124, 2000 (in Chinese).
262. Yao, D.H., and He, Y.J.: Study on the biomass and productivity of Pinus massoniana air-seeding forests, Journal of Hunan Environmental-Biological Polytechnic, 7(1), 23-27, 2001 (in Chinese).
263. Yao, D.H., and Li, Z.H.: Study on the biomass dynamics of Cryptomeria japonica plantation, Scientia Silvae Sinicae, 33(S2), 203-207, 1997 (in Chinese).
264. Yao, D.H., Yang, M.S., and Li, Z.H.: Effect of stand density on the biomass and productivity of Eucalyptus grandis × E. urophylla plantation, Journal of Central South Forestry University, 20(3), 20-23, 2000 (in Chinese).
265. Yao, Y.J., Kang, W.X., and Tian, D.L.: Study on the biomass and productivity of Cinnamomum camphora plantation, Journal of Central South Forestry University, 23(1), 1-5, 2003 (in Chinese).
266. Ye, J.Z., and Jiang, Z.L.: Study on the biomass and its distribution of Cunninghamia lanceolata plantations in the hilly regions of the south Jiangsu Province, Acta Ecologica Sinica, 3(1), 7-14, 1983 (in Chinese).
267. Ye, J.Z., Jiang, Z.L., Zhou, B.L., Han, F.Q., and Chen, S.B.: Annual dynamics of the biomass of Chinese fir forests in Yangkou Forest Farm, Fujian Province, Journal of Nanjing Institute of Forestry, (4), 1-9, 1984 (in Chinese).
268. Ye, S.M., Zheng, X.X., Xie, W.D., and Zhao, L.J.: Sprout regeneration and plant regeneration influences on the yield of Eucalyptus urophylla × E. grandis plantation, Journal of Nanjing Forestry University (Natural Science Edition), 31(3), 43-46, 2007 (in Chinese).
269. Ye, S.M., Zheng, X.X., Yang, M., Xie, W.D., Zhao, L.J., and Liang, H.W.: Biomass and productivity of stratified mixed stands of Eucalyptus urophylla and Acacia mangium, Journal of Beijing Forestry University, 30(3), 37-43, 2008 (in Chinese).
270. Yi, A.Y.: Biomass and productivity of mixed plantation of Cryptomeria japonica and Cunninghamia lanceolata, Sichuan Forestry Exploration and Design, (3), 50-52, 59, 1998 (in Chinese).
271. Yi, W.M., Zhang, Z.P., Ding, M.M., and Wang, B.S.: Biomass and efficiency of radiation utilization in Erythrophleum fordii community, Acta Ecologica Sinica, 20(2), 397-403, 2000 (in Chinese).
272. Yu, B., Zhang, Q.L., Wang, L.M., and Wu, J: Characteristics of biomass and productivity in Larix gmelinii natural forests with different stand structures, Journal of Zhejiang Agriculture and Forestry University, 28(1), 52-58, 2011 (in Chinese).
273. Yuan, C.M., Lang, N.J., Meng, G.T., Fang, X.J., Li, G.X., and Wen, S.L.: Community structure characteristics and biological productivity of Acacia dealbata protective plantation in Toutang mountainous area, Yunnan Forestry Science and Technology, (2), 24-27, 1998 (in Chinese).
274. Yuan, C.M., Lang, N.J., Meng, G.T., Fang, X.J., Li, G.X., and Wen, S.L.: The structural feature and biomass of soil-water conservative plantation of Armand pine in the upper reach of the Yangtze River, Journal of Northeast Forestry University, 30(3), 5-7, 2002 (in Chinese).
275. Zeng, X.P., Peng, S.L., and Zhao, P.: Measurement of respiration amount in artificial Acacia mangium froest in a low subtropical hill forest region of Guangdong, Acta Phytoecologica Sinica, 24(4), 420-424, 2000 (in Chinese).
276. Zhai, B.G., Song, C.H., Zhang, H.D., and Wang, W.X.: Study on the biomass and productivity of Pinus tabuliformis plantation at a permanent plot in Taiyue Forest Region, Shanxi Province, Journal of Beijing Forestry University, 14(S1), 156-163, 1992 (in Chinese).
277. Zhang, B.L.: Study on the biomass and productivity of Quercus liaotungensis stands in Ziwuling Forest Region, Shaanxi Province, Journal of Northwestern College of Forestry, 5(1), 1-7, 1990 (in Chinese).
278. Zhang, W.Z.: The biomass and productivity of Pinus elliottii plantation in Pingnan County, Forest Investigation Design, (2), 11-16, 2010b (in Chinese).
279. Zhang, Z.J.: Study on spatial characteristics of Pinus massoniana biomass and root distribution in an acid rain area, Chongqing, Thesis for Master’s Degree, Agricultural University of Hebei, Baoding, China, 50pp., 2006 (in Chinese).
280. Zhang, Z.J., Wang, Y.H., Yuan, Y.X., Li, Z.Y., Cao, L., Zhang, G.Z., Yu, P.T., and Wang, Y.: Study on the biomass and distribution of Pinus massoniana natural secondary forest, Journal of Agricultural University of Hebei, 29(5), 37-43, 2006 (in Chinese).
281. Zhang, B.L., and Chen, C.G.: Biomass and productivity of Robinia pseudoacacia plantation in Hongxing Forest Farm of Changwu County, Shaanxi Province, Shaanxi Forest Science and Technology, (3), 13-17, 1992 (in Chinese).
282. Zhang, C.L., and Zhou, X.F.: Biomass of a natural secondary birch stand, in: Long-term Located Research on Forest Ecosystems, Volume 1, Zhou, X.F., Wang, Y.H., Zhao, H.X., eds., Northeast Forestry University Press, Harbin, China, 428-435, 1991 (in Chinese).
283. Zhang, F., and Shangguan, T.L.: Synecological features and biomass of Larix principis-rupprechtii forest in Guandi Mountain, Shanxi Province, Journal of Shanxi University (Natural Science Edition), 15(1), 72-77, 1992 (in Chinese).
284. Zhang, S.Y., and Pan, C.D.: The compatible biomass model of Picea schrenkiana young plantation, Journal of Fujian College of Forestry, 22(3), 201-204, 2002 (in Chinese).
285. Zhang, G.B., Li, X.Q., She, X.S., Hu, C.Q., and Hu, G.H.: Biomass characteristics of dominant tree species (group) in Lingnan Forest Farm, Anhui Province, Scientia Silvae Sinicae, 48(5), 136-140, 2012 (in Chinese).
286. Zhang, J.W., Chen, C.Y., Deng, S.J., and Feng, Z.W.: Comparison of the mathematical patterns for estimating the standing crop of Cunninghamia lanceolata plantation, Journal of Northeastern Forestry Institute, 12(4), 1-6, 1984 (in Chinese).
287. Zhang, J.X., Zhou, W., Li, Y., Luo, W., and Xie, C.M.: The biomass of Chinese corktree plantations, Non-wood Forest Research, 8(1), 35-40, 1990 (in Chinese).
288. Zhang, L., Huang, Y., Luo, T.X., Dai, Q., and Deng, K.M.: Age effects on stand biomass allocations to different components: A case study of Cunninghamia lanceolata plantations and Pinus massoniana plantations, Journal of the Graduate School of the Chinese Academy of Sciences, 22(2), 170-178, 2005 (in Chinese).
289. Zhang, Q., Fan, S.H., Liu, G.L., Feng, H.X., Zong, Y.C., and Fei, B.H.: A study on biomass and productivity of Populus × euramericana cv. ‘San Martino’ (I-72/58) plantation on beach land of Yangtze River, Forest Research, 21(4), 542-547, 2008a (in Chinese).
290. Zhang, S.G., Liu, J., Huang, K.Y., Liang, R.L., and Lan, X.: Biomass and distribution patterns of Pinus massoniana plantation in northwest Guangxi, Guangxi Forestry Science, 39(4), 189-192, 219, 2010 (in Chinese).
291. Zhang, W.H., Wang, Y.P., Kang, Y.X., and Liu, X.J.: Study on the relationship between Larix chinensis population’s structure and environment factors, Acta Ecologica Sinica, 24(2), 41-47, 2004b (in Chinese).
292. Zhang, W.Q., Peng, S.L., Ren, H., and Peng, Z.W.: The allocation of biomass and energy in Acacia mangium forest, Acta Ecologica Sinica, 15(S1): 44-48, 1995 (in Chinese).
293. Zhang, Z.H., Li, Y., and Xie, R.G.: A preliminary study on the growth and biomass of the mixed plantation of Magnolia officinalis and Cunninghamia lanceolata, Journal of Fujian Forestry Science and Technology, 23(3), 28-31, 1996b (in Chinese).
294. Zhao, L.: Study on the biomass and its allocation of Schima superba planted forests, Jiangxi Forestry Science and Technology, (4), 5-7, 59, 2006 (in Chinese).
295. Zhao, K., and Tian, D.L.: Study on the biomass and productivity of mature Chinese fir stand in Huitong County, Journal of Central South Forestry University, 20(1), 7-13, 2000 (in Chinese).
296. Zhao, T.S., and Zhang, P.C.: Comprehensive effects of tending and felling on Pinus taiwanensis plantation, Acta Agriculturae Universitatis Henanensis, 23(4), 409-421, 1989 (in Chinese).
297. Zhao, G.L., Wang, J.X., Wang, X.Z., Shen, Y.B., and Zhou, J.C.: Nutrient element cycling and density effects of Pinus tabuliformis plantations, Journal of Beijing Forestry University, 28(4), 39-44, 2006 (in Chinese).
298. Zhao, J.M., Wu, Z.W., and Xie, S.X.: Individual growth and biomass characteristics of wild Idesia polycarpa in Guizhou, Guizhou Forestry Science and Technology, 40(4), 7-13, 23, 2012 (in Chinese).
299. Zhong, Q.L., Zhang, Z.Y., Zhang, C.H., Zhou, H.L., and Huang, Z.Q.: The dynamic analysis of the biomass and its structure of Machilus pauhoi, Acta Agriculturae Universitatis Jiangxiensis, 23(4), 533-536, 2001 (in Chinese).
300. Zhou, S.Q., and Huang, J.Y.: Biomass estimation models of Larix mastersiana plantation, Journal of Sichuan Forestry Science and Technology, 12(2), 67-69, 1991a (in Chinese).
301. Zhou, S.Q., and Huang, J.Y.: A study on biomass and productivity of Larix mastersiana plantation in Sichuan, Acta Phytoecologica et Geobotanica Sinica, 15(1): 9-16, 1991b (in Chinese).
302. Zhou, G.M., Yao, J.X., Qiao, W.Y., Yang, Q.H., Zhu, G.J., and Xu, W.Y.: Biomass of Chinese fir planted forest in Qingyuan County of Zhejiang Province, Journal of Zhejiang Forestry College, 13(3), 235-242, 1996 (in Chinese).
303. Zhou, G.Y., Zeng, Q.B., Lin, M.X., Chen, B.F., Li, Y.D., and Wu, Z.M.: Study on the biomass and nutrient allocation in Manglietia hainanensis plantation ecosystem at Jianfengling, Hainan Province, Forest Research, 10(5), 453-457, 1997 (in Chinese).
304. Zhou, Z.Z., Zheng, H.S., Yin, G.T., Yang, Z.J., and Chen, K.T.: Biomass equations for rubber tree in southern China, Forest Research, 8(6), 624-629, 1995 (in Chinese).
305. Zhu, B.L., Li, Z.H., and Chen, S.X.: Effect of stand density on the biomass and productivity of Eucalyptus urophylla × E. grandis plantation, Journal of Hunan Environmental-Biological Polytechnic, 13(4), 11-14, 2007 (in Chinese).
306. Zhu, X.W., Xiao, Y., and Cai, W.C.: A preliminary study on the biomass of Populus davidiana natural forest, Science and Technology of Qinghai Agriculture and Forestry, (1), 30-34, 1988 (in Chinese).
307. Zhuang, H.L., Becuwe, X., Xiao, C.B., Wang, Y.H., Wang, H., Yin, B., and Liu, C.J.: Allometric equation-based estimation of biomass carbon sequestration in Metasequoia glyptostroboides plantations in Chongming Island, Shanghai, Journal of Shanghai Jiaotong University (Agricultural Science Edition), 29(2), 48-55, 2012 (in Chinese).
308. Ayala R (1998) Ecuaciones para estimar biomasa de pinos y encinos en la meseta central de Chiapas. Thesis, Universidad Autónoma de Chapingo.
309. Hytönen, J., A. Saarsalmi & P. Rossi (1995). Biomass production and nutrient uptake of short-rotation plantations. Silva Fennica 29:2, 117-139.
310. Cerný, M. 1990. Biomass of Picea abies (L.) Karst. in midwestern Bohemia. Scandinavian Journal of Forest Research 5: 83-95.
311. Matis, K. & Alifragis, D. 1983-1984. Aboveground biomass of oaks (Quercus conferta Kit.) in Taxiarchis Greece. Scientific Annals of the Depart. of Forestry and Natural Envir. KA 15.
312. STANGL, R., HOCHBICHLER, E., BELLOS, P. N. & FLORINETH, F. 2009. Allometric estimation of the above-ground biomass components of Alnus incana (L.) Moench used for landslide stabilisation at Bad Goisern (Austria). Plant Soil, 324, 115-129.
313. BPKH Wilayah XI Jawa-Madura &MFP II. (2009) Alometrik berbagai jenis pohon untuk menaksir kandungan biomassa dan karbon di Hutan Rakyat. Laporan BPKH Wilayah XI Jawa-Madura &MFPII, Yogyakarta.
314. Ismail AY (2005) Dampak Kebakaran Hutan Terhadap Potensi Kandungan Karbon Pada Tanaman Acacia mangium Willd Di Hutan Tanaman Industri (HTI). Unpublished Postgraduate thesis, Institut Pertanian Bogor.
315. Dharmawan IWS, Siregar CA (2008) Karbon Tanah dan Pendugaan Karbon Tegakan Avicennia marina (Forsk.) Vierh. Di Ciasem, Purwakarta. Jurnal Penelitian Hutan dan Konservasi Alam 5(4):317-328.
316. Kusumana C, Sabiham S, Abe K, Watanabe H (1992) An estimation of above ground tree biomass of a mangrove forest in East Sumatra, Indonesia. Tropics 1(4): 243-257.
317. Yulyana R (2005) Potensi Kandungan Karbon Pada Pertanaman Karet (Hevea brasiliensis) yang Disadap. Studi kasus di Perkebunan Inti Rakyat Kecamatan Pondok Kelapa, Kabupaten Bengkulu Utara. Unpublished Postgraduate thesis, Institut Pertanian Bogor.
318. Silalahi H (2007) Penaksiran Kandungan Karbon Hutan Berdasarkan Ukuran Diameter Batang dan Tinggi Pohon (Studi Kasus Pada Hutan Rakyat Jenis Sengon di Dusun Kebondalem, Desa Sukorejo, Kecamatan Mojotengah, Kabupaten Wonosobo). Unpublished Undergraduate Thesis, Universitas Gadjah Mada.
319. Azhim MF (2007) Penaksiran Potensi Kandungan Karbon Pada Hutan Rakyat Jenis Sengon (Studi Kasus di Desa Bateh, Kecamatan Candimulyo, Magelang).Unpublished Undergraduate Thesis, Universitas Gadjah Mada.
320. Siregar CA (2007b) Formulasi Allometri Biomassa dan Konservasi Karbon Tanah Hutan Tanaman Sengon (Paraserianthes falcataria (L.) Nielsen) di Kediri. Jurnal Penelitian Hutan dan Konservasi Alam 4(2): 169-181.
321. Siringoringo HH, Siregar CA (2006) Model Persamaan Allometri Biomasa Total Untuk Estimasi Akumulasi Karbon Pada Tanaman Paraserianthes falcataria (L.) Nielsen Jurnal Penelitian Hutan dan Konservasi Alam 3 (5): 541-553.
322. Siregar CA (2007a) Pendugaan Biomassa Pada Hutan Tanaman Pinus (Pinus merkusii Jungh et de Vriese) dan Konservasi Karbon Tanah di Cianten, Jawa Barat. Jurnal Penelitian Hutan dan Konservasi Alam 4(3):251-266.
323. Heriyanto NM, Siringoringo HH, Miyakuni K, Yoshiyuki K (2005) Allometric Equations and Other Parameters for Estimating the Amount of Biomass in Pinus merkusii Forests. In FORDA & JICA. Proceedings of the 2nd Workshop on Demonstration Study on Carbon Fixing Forest Management in Indonesia: How to increase the welfare of local people through the sustainable forest management. Bogor, January 11, 2005.
324. Anonim (2005) Allometric Equations and Other Parameters for Estimating Biomass Amount of Pinus merkusii Jungh. Et de Vr. In FORDA and JICA. Terminal Report on Carbon Fixing Forest Management Project in Indonesia. December, 2005.
325. Salim (2005) Profil Kandungan Karbon Pada tegakan Puspa (Schima wallichii Korth.) Unpublished Postgraduate thesis, Institut Pertanian Bogor (Bogor Agriculture University).
326. Handayani K (2003) Model Pendugaan Biomassa Shorea leprosula MIQ di Kebun Percobaan Carita. Unpublished Undergraduate thesis, Institut Pertanian Bogor.
327. Adinugroho WC, Sidiyasa K (2006) Model Pendugaan Biomassa Pohon Mahoni (Swietenia macrophylla King) Di Atas Permukaan Tanah. Jurnal Penelitian Hutan dan Konservasi Alam 3(1): 103 - 117.
328. Aminudin S (2008) Kajian Potensi Cadangan Karbon Pada Pengusahaan Hutan Rakyat. Studi kasus Hutan Rakyat Desa Dengok, Kecamatan Playen, Kabupaten Gunungkidul. Unpublished Postgraduate thesis, Institut Pertanian Bogor.
329. Budiadi, Sabarnurdin MS (2001) Struktur Biomassa di Atas dan Bawah Permukaan Tanah Tanaman Jati Dengan Modifikasi Pola Tanam. In Buletin Kehutanan No. 47/ 2001: 49-60.
330. Heriansyah I, Miyakuni K, Kato T, Kiyono Y, Kanazawa Y (2007) Growth characteristics and biomass accumulations of Acacia mangium under different management practices in Indonesia. J Trop For Sci. 19(4): 226-235.
331. Hozumi, K., Yoda, K., Kokawa, S., Kira, T., 1969. Production ecology of tropical rain forests in southwestern Cambodia. I. Plant biomass. In: Kira, T., Iwata, K. (Eds.), Nature and Life in Southeast Asia. Japan Society for the Promotion of Science, Tokyo, pp. 1-51.
332. Kangkuso, A., Jamili, J., Septiana, A., Raya, R., Sahidin, I., Rianse, U., Rahim, S., Alfirman, A., Sharma, S., Nadaoka, K., 2015. Allometric models and aboveground biomass of Lumnitzera racemosa Willd. forest in Rawa Aopa Watumohai National Park, Southeast Sulawesi, Indonesia. Forest Science and Technology xx, 1-8.
333. Kridiborworn, P., Chidthaisong, A., Yuttitham, M., Tripetchkul, S., 2012. Carbon sequestration by mangrove forest planted specifically for charcoal production in Yeesarn, Samut Songkram. Journal of Sustainable Energy & Environment 3, 87-92.
334. Nipithwittaya, S., Bualert, S., 2012. Above ground carbon sequestration in mangrove forest filtration system. Journal of Applied Sciences 12, 1537-1546.
335. Poungparn, S., Komiyama, A., Patanaponpaipoon, P., Jintana, V., Sangtiean, T., Tanapermpool, P., Piriyayota, S., Maknual, C., Kato, S., 2003. Site-independent allometric relationships for estimating above-ground weights of mangroves. Tropics 12, 147-158.
336. Komiyama, A., Havanond, S., Srisawatt, W., Mochida, Y., Fujimoto, K., Ohnishi, T., Ishihara, S., Miyagi, T., 2000. Top/root biomass ratio of a secondary mangrove (Ceriops tagal (Perr.) C.B. Rob.) forest. Forest Ecology and Management 139, 127-134.
337. Nipithwittaya, S., Bualert, S., 2012. Above ground carbon sequestration in mangrove forest filtration system. Journal of Applied Sciences 12, 1537-1546.
338. Nguyen, T.K., Ninomiya, I., 2007. Allometric relations for young Kandelia candel (L.) Blanco plantation in Northern Vietnam. Journal of Biological Sciences 7, 539-543.
339. Toma, T., Ishida, A., Matius, P., 2005. Long-term monitoring of post-fire aboveground biomass recovery in a lowland dipterocarp forest in East Kalimantan, Indonesia. Nutrient Cycling in Agroecosystems 71, 63-72.
340. Purwanto, R.D., Shiba, M., 2005. Allometric equations for estimating above ground biomass and leaf area of planted teak (Tectona grandis) forests under agroforestry management in East Java, Indonesia. Forest Research Kyoto 76, 1-8.
341. Krisnawati, H., Adinugroho, W.C., Imanuddin, R., 2012. Monograph: Allometric models for estimating tree biomass at various forest ecosystem types in Indonesia. Research and Development Center for Conservation and Rehabilitation, Forestry Research and Development Agency, Bogor, Indonesia.
342. Kueh, R.J., Lim, M.T., 1999. An estimate of forest biomass in Ayer Hitam Forest Reserve. Pertanika Journal of Tropical Agricultural Science 22, 117-123.
343. Mahat, M.N., Fauzi, M.A.F.A., Azam, F.A., Zolkifle, M.F., 2014. Growth and biomass accumulation of 5 years old Hopea odorata plantation in three different soil series of ultisols. Journal of Agricultural Research and Development 4, 15-22.
344. Jundang, W., Puangchit, L., Diloksumpun, S., 2010. Carbon storage of dry dipterocarp forest and eucalypt plantation at Mancha Khiri plantation, Khon Kaen province. Thai Journal of Forestry 29, 36-44 (in Thai with English abstract).
345. Kamo, K., Vacharangkura, T., Tiyanon, S., Viriyabuncha, C., Nimpila, S., Duangsrisen, B., Thaingam, R., Sakai, M., 2008. Biomass and dry matter production in planted forests and an adjacent secondary forest in the grassland area of Sakaerat, northeastern Thailand. Tropics 17, 209-224.
346. Nongnuang, S., 2012. Carbon sinks and nutrient accumulation in ecosystems of series of Pinus Kesiya plantations and fragmented forests in Boakaew highland watershed, Chiang Mai province. PhD thesis. Chiang Mai University, Chiang Mai.
347. Diloksumpun, S., Staporn, D., 2009. Carbon storage of Eucalypts planted on paddy bunds in Chachoengsao province. Thai Journal of Forestry 28, 72-84 (in Thai with English abstract).
348. Khuncharoensri, J., Amkamphon, K., Poolsiri, R., 2013. Growth, biomass and carbon storage of Jatropha curcas L. Thai Journal of Forestry 32, 1-11 (in Thai with English abstract).
349. Birk E.M., Walker C. Ryan P. Briggs G. and Harrison J. Stand growth and productivity. Ryan P.J. editor. Factors affecting the establishment and management of tree stands on rehabilitated coal mines in the Hunter Valley, NSW. Beecroft, Australia: Research Division, State Forests of NSW; 1995; pp. 80-110.
350. Madgwick H.A.I., Oliver G. R. Frederick D. J. and Thompson Tew D. Estimating the dry weights of Eucalyptus trees - central North Island, New Zealand. Bioresource Technology. 1991; 37:111-114.
351. Baker T.G., Attiwill P. M. and Stewart H. T. L. Biomass equations for Pinus radiata in Gippsland, Victoria. NZ J. For. Sci. 1984; 14:89-9
352. Mälkönen, E. & Saarsalmi, A. 1982. Hieskoivikon biomassatuotos ja ravinteiden menetys kokopuun korjuussa. Folia Forestalia 534: 1–17.
353. Cerný, M. 1990. Biomass of Picea abies (L.) Karst. in midwestern Bohemia. Scandinavian Journal of Forest Research 5: 83–95.
354. Ingerslev, M. & Hallbäcken, L. 1999. Above ground biomass and nutrient distribution in a limed and fertilized Norway spruce (Picea abies) plantation. Part II. Accumulation of biomass and nutrients. Forest Ecology and Management 119: 21–38.
355. Carvalho, J.P. & Parresol, B.R. 2003. Additivity in tree biomass components of Pyrenean oak (Quercus pyrenaica Willd.). Forest Ecology and Management 179: 269–276.
356. Cairns M, Olmsted I, Granados J, Argaez J (2003) Composition and aboveground tree biomass of a dry semi-evergreen forest on Mexico’s Yucatan Peninsula. For Ecol Manage 186:125–132.
357. Brown S., Gillespie A.J., and Lugo A.E., 1989. Biomass estimation methods for tropical forests with aplications to forest inventory data. For. Sci. 35: 881–902.
358. Cole T.G. and Ewel J.J., 2006. Allometric equations for tour valuable tropical tree species. For. Ecol. Manage. 229: 351–360.
359. Tuskan, G.A., Rensema, T.R., 1992. Clonal differences in biomass characteristics, coppice ability, and biomass prediction equations among four Populus clones grown in North Dakota. Can. J. For. Res. 22, 348–354.
360. Wiant, H.V.,Jr.;Castaneda,F.;Sheetz,C.E.;Colaninno,A.;De-Moss,J.C.Equations for predicting weights of some appalachian hardwoods.1979;W.Va.For.Notes 7:21-26.
361. Schlaegel,B.E.Yields of four 40-year-old conifers and aspen in adjacent stands.Can.J.For.Res.5:278-280;1975.
362. Clark,A.,Ⅲ；Phillips,D.R.;Hitchcock,H.C.Predicted weights and volumes of scarlet oak trees on the Tennessee Cumberland plateau.1980a;USDA For.Serv.Res.Pap.SE-214.23P
363. Zavitkovsi, J., Jeffers, R.M., Nienstaedt, H., Strong, T.F., 1981.Biomass production of several jack pine provenances at three Lake States locations. Can. J. For. Res. 11, 441–447.
