# Supplementary material for: Global patterns of allometric model parameters prediction
Source: Sci Rep. 2023 Jan 27;13:1550. doi: 10.1038/s41598-023-28843-2 (PMC9883259; doi:10.1038/s41598-023-28843-2)

**Global patterns of allometric model parameters prediction**

Zixuan Wang^1^ Xingzhao Huang^1*^ Fangbing Li^1^ Dongsheng Cheng^2^ Xiaoniu Xu^1^

^1^School of Forestry & Landscape of Architecture, Anhui Agricultural University, Hefei 230036, China

^2^Key Laboratory of Tree Breeding and Cultivation, State Forestry Administration, Research Institute of Forestry, Chinese Academy of Forestry, Beijing 100091, China

* Corresponding author: xingzhaoh@163.com

**Authorship**

X.Z. designed the study. X.Z. and Z.X. collected the data. Z.X. and F.B. analyzed the data. All authors contributed significantly to the writing of the manuscript.

**Supplementary materials**

**Table S1 Variables selection (LnW=a+b*Ln(D))**

| Number | Combination of variables | Variables importance | | InCMSE(Mean Decrease Accuracy)(%) | | Var explained(%) | | RSS(Mean of squared residuals) | |
| --- | --- | --- | --- | --- | --- | --- | --- | --- | --- |
|  |  | a | b | a | b | a | b | a | b |
| 1 | family | 18.51 | 1.52 | 0.069 | 0.006 | 66.21 | 49.96 | 0.1835 | 0.0151 |
|  | genus | 16.80 | 1.13 | 0.075 | 0.005 |  |  |  |  |
|  | species | 20.61 | 1.27 | 0.070 | 0.004 |  |  |  |  |
|  | MAT | 29.85 | 1.26 | 0.209 | 0.004 |  |  |  |  |
|  | MAP | 24.45 | 1.49 | 0.157 | 0.005 |  |  |  |  |
|  | altitude | 27.86 | 1.71 | 0.204 | 0.006 |  |  |  |  |
|  | aspect | 19.92 | 1.22 | 0.117 | 0.004 |  |  |  |  |
|  | SOC | 29.25 | 1.41 | 0.185 | 0.004 |  |  |  |  |
|  | slope | 27.00 | 1.01 | 0.166 | 0.004 |  |  |  |  |
|  | clay | 20.34 | 1.05 | 0.097 | 0.003 |  |  |  |  |
|  | soil type | 15.19 | 0.51 | 0.111 | 0.001 |  |  |  |  |
| 2 | species | 27.06 | 2.06 | 0.066 | 0.005 | 65.05 | 47.21 | 0.1897 | 0.0153 |
|  | MAT | 34.61 | 1.34 | 0.238 | 0.005 |  |  |  |  |
|  | MAP | 29.68 | 1.61 | 0.188 | 0.006 |  |  |  |  |
|  | altitude | 31.92 | 1.88 | 0.227 | 0.007 |  |  |  |  |
|  | aspect | 23.76 | 1.31 | 0.146 | 0.005 |  |  |  |  |
|  | SOC | 31.01 | 1.53 | 0.207 | 0.004 |  |  |  |  |
|  | slope | 29.54 | 1.06 | 0.184 | 0.005 |  |  |  |  |
|  | clay | 21.54 | 1.08 | 0.117 | 0.004 |  |  |  |  |
|  | soil type | 17.91 | 0.53 | 0.131 | 0.002 |  |  |  |  |
| 3 | MAT | 38.71 | 1.39 | 0.279 | 0.005 | 66.18 | 48.04 | 0.1836 | 0.0160 |
|  | MAP | 33.91 | 1.71 | 0.218 | 0.006 |  |  |  |  |
|  | altitude | 34.01 | 1.99 | 0.261 | 0.008 |  |  |  |  |
|  | aspect | 26.41 | 1.41 | 0.163 | 0.006 |  |  |  |  |
|  | SOC | 33.81 | 1.11 | 0.219 | 0.005 |  |  |  |  |
|  | slope | 32.84 | 1.63 | 0.207 | 0.006 |  |  |  |  |
|  | clay | 23.91 | 1.14 | 0.131 | 0.004 |  |  |  |  |
|  | soil type | 19.66 | 0.56 | 0.147 | 0.002 |  |  |  |  |
| 4 | MAT | 43.23 | 1.50 | 0.303 | 0.006 | 66.10 | 47.01 | 0.1841 | 0.0160 |
|  | MAP | 35.90 | 1.80 | 0.228 | 0.006 |  |  |  |  |
|  | altitude | 37.78 | 2.11 | 0.271 | 0.008 |  |  |  |  |
|  | aspect | 25.99 | 1.46 | 0.157 | 0.006 |  |  |  |  |
|  | SOC | 38.23 | 1.22 | 0.234 | 0.005 |  |  |  |  |
|  | slope | 37.74 | 1.72 | 0.247 | 0.006 |  |  |  |  |
|  | soil type | 22.68 | 0.66 | 0.157 | 0.002 |  |  |  |  |
| 5 | MAT | 51.34 | 1.68 | 0.354 | 0.006 | 64.3 | 47.5 | 0.1983 | 0.0158 |
|  | MAP | 43.84 | 1.89 | 0.252 | 0.006 |  |  |  |  |
|  | altitude | 43.00 | 2.21 | 0.285 | 0.008 |  |  |  |  |
|  | aspect | 30.41 | 1.55 | 0.187 | 0.006 |  |  |  |  |
|  | slope | 44.97 | 1.80 | 0.263 | 0.006 |  |  |  |  |
|  | soil type | 26.47 | 0.71 | 0.195 | 0.002 |  |  |  |  |

**Table S2 Random forest parameter optimization (LnW=a+b*Ln(D))**

| **Predictors** | **ntree** | **mtry** | **nodesize** | **Var explained(%)** | |
| --- | --- | --- | --- | --- | --- |
|  |  |  |  | **Parameter a** | **Parameter b** |
| Family  Genus  Species  MAT  MAP  Altitude  Aspect  SOC  Slope  Clay  Soil type  Parameter a | 1000 | 2 | 3 | 65.96 | 45.90 |
|  |  |  | 5 | 64.26 | 45.39 |
|  |  |  | 7 | 61.63 | 44.77 |
|  |  | 3 | 3 | 66.05 | 48.78 |
|  |  |  | 5 | 64.72 | 48.77 |
|  |  |  | 7 | 63.02 | 48.74 |
|  |  | 4 | 3 | 65.69 | 50.03 |
|  |  |  | 5 | 64.69 | 49.55 |
|  |  |  | 7 | 63.10 | 49.51 |
|  | 2000 | 2 | 3 | 65.98 | 45.96 |
|  |  |  | 5 | 64.08 | 45.91 |
|  |  |  | 7 | 61.47 | 44.76 |
|  |  | 3 | 3 | 66.16 | 49.09 |
|  |  |  | 5 | 64.93 | 48.75 |
|  |  |  | 7 | 63.18 | 48.21 |
|  |  | 4 | 3 | 65.66 | 50.22 |
|  |  |  | 5 | 64.63 | 49.74 |
|  |  |  | 7 | 63.13 | 49.37 |
|  | 3000 | 2 | 3 | 65.84 | 46.19 |
|  |  |  | 5 | 63.99 | 45.58 |
|  |  |  | 7 | 61.45 | 44.97 |
|  |  | 3 | 3 | 66.21 | 49.96 |
|  |  |  | 5 | 64.83 | 48.63 |
|  |  |  | 7 | 63.25 | 48.44 |
|  |  | 4 | 3 | 65.67 | 50.11 |
|  |  |  | 5 | 64.77 | 50.00 |
|  |  |  | 7 | 62.90 | 49.60 |

**Table S3** **The distribution of model (LnW=a+b*Ln(D)) parameters in different zones among six continents.** The division of climate zones: tropics:-23°26’~23°26’; temperate zone:23°26’~ 66°34’,-23°26’~ -66°34’; cold zone:66°34’~90°00’,-66°34’~-90°00’ .

| Continent | Zone | Parameter a  （mean±stand error） | Parameter b  （mean±stand error） |
| --- | --- | --- | --- |
| Africa | tropics | -2.26 ± 0.0059 | 2.41 ± 0.0018 |
| Asia | tropics | -2.26 ± 0.0060a | 2.40 ± 0.0022a |
|  | temperate zone | -2.46 ± 0.0170b | 2.37 ± 0.0059a |
| South America | tropics | -2.10 ± 0.0086 | 2.39 ± 0.0025 |
| North America | tropics | -2.28 ± 0.0271a | 2.39 ± 0.0087a |
|  | temperate zone | -2.49 ± 0.0066b | 2.38 ± 0.0014a |
|  | cold zone | -2.63 ± 0.0111c | 2.42 ± 0.0029b |
| Europe | temperate zone | -2.67 ± 0.0052a | 2.41 ± 0.0010a |
|  | cold zone | -3.51 ± 0.0488b | 2.39 ± 0.0053a |
| Oceania | temperate zone | -2.19 ± 0.0320 | 2.33 ± 0.0100 |

**Figure S1. The process of articles acquisition and filtering.**


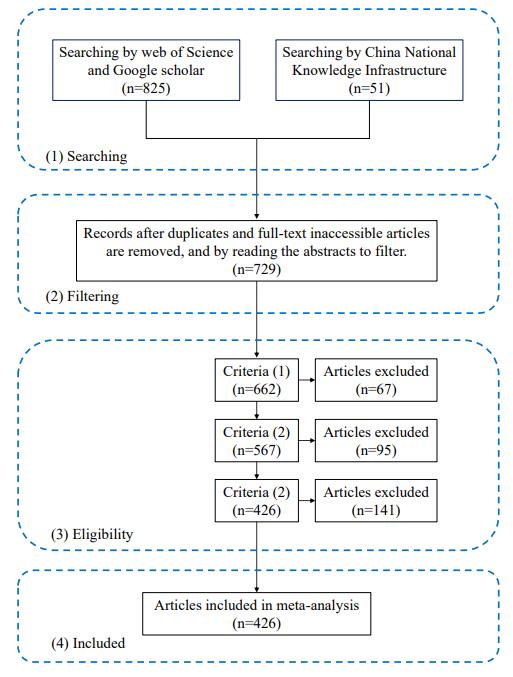


**Figure S2. Correlation of allometric model (LnW=a+b*Ln(D)) parameters and other nine factors.** (MAT indicates mean annual temperature, MAP indicates mean annual precipitation, SOC indicates soil organic carbon)


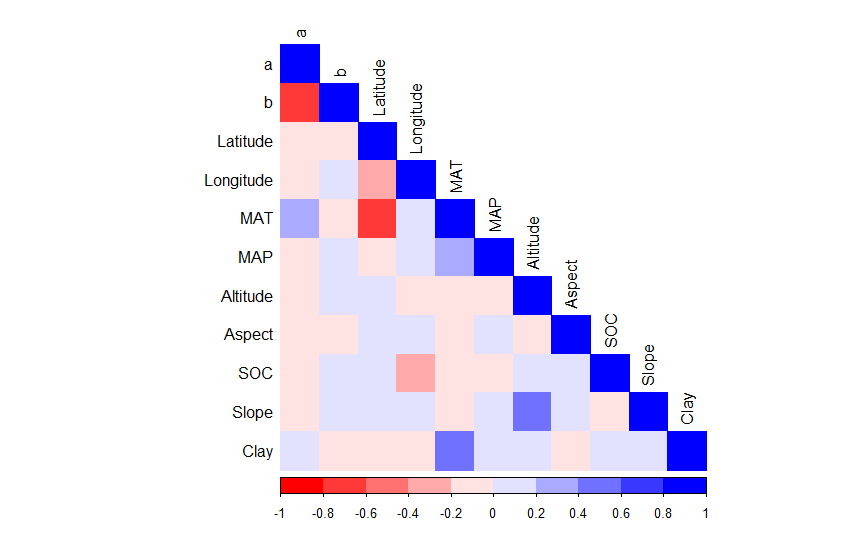


**Figure S3. Correlation of allometric model (LnW=a+b*Ln(D^2^H)) parameters and other nine factors.** (MAT indicates mean annual temperature, MAP indicates mean annual precipitation, SOC indicates soil organic carbon)


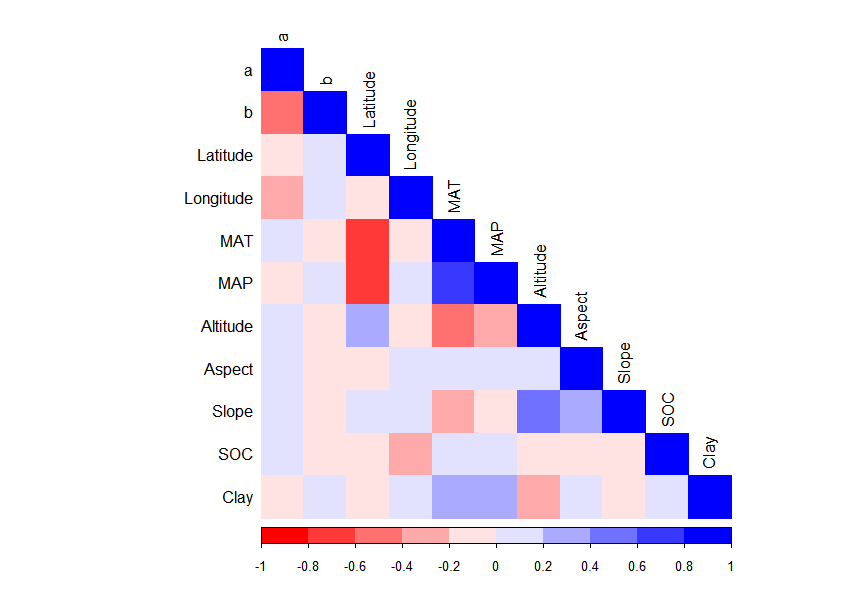

Supplement: Supplementary file 1 — Supplementary Information. [file 41598_2023_28843_MOESM1_ESM.zip › Supplement materials/Supplementary materials.docx]
